# Supplementary material for: A cyclic peptide toolkit reveals mechanistic principles of peptidylarginine deiminase IV regulation
Source: Nat Commun. 2024 Nov 11;15:9746. doi: 10.1038/s41467-024-53554-1 (PMC11555231; doi:10.1038/s41467-024-53554-1)
Supplement: Supplementary file 1 — Supplementary Information [file 41467_2024_53554_MOESM1_ESM.pdf]

A cyclic peptide toolkit reveals mechanistic principles of peptidylarginine deiminase IV regulation

M. Teresa Bertran [a#], Robert Walmsley [b#], Thomas Cummings [b], Iker Valle Aramburu [c], Donald J. Benton [d], Rocio Mora Molina[b], Jayalini Assalaarachchi [b], Maria Chasampalioti [b], Tessa Swanton [c], Dhira Joshi [e], Stefania Federico [e], Hanneke Okkenhaug [f], Lu Yu [g], David Oxley [g], Simon Walker [f], Venizelos Papayannopoulos [c], Hiroaki Suga [h], Maria A. Christophorou [b*], Louise J. Walport [a,h,i *]

[a] Protein-Protein Interaction Laboratory, The Francis Crick Institute, London, NW1 1AT, UK
[b] Epigenetics, The Babraham Institute, Cambridge, CB22 3AT, UK
[c] Antimicrobial Defense Laboratory, The Francis Crick Institute, London, NW1 1AT, UK
[d] Structural Biology, The Francis Crick Institute, London, NW1 1AT, UK
[e] Chemical Biology, The Francis Crick Institute, London, NW1 1AT, UK
[f] Imaging, The Babraham Institute, Cambridge, CB22 3AT, UK
[g] Proteomics, The Babraham Institute, Cambridge, CB22 3AT, UK
[h] The University of Tokyo, Hongo, Bunkyo-ku, Tokyo, 113-0033, Japan
[i] Imperial College London, Department of Chemistry, London, W12 0BZ, UK

[#] These authors contributed equally to this study
*To whom correspondence should be addressed: l.walport@imperial.ac.uk or maria.christophorou@babraham.ac.uk

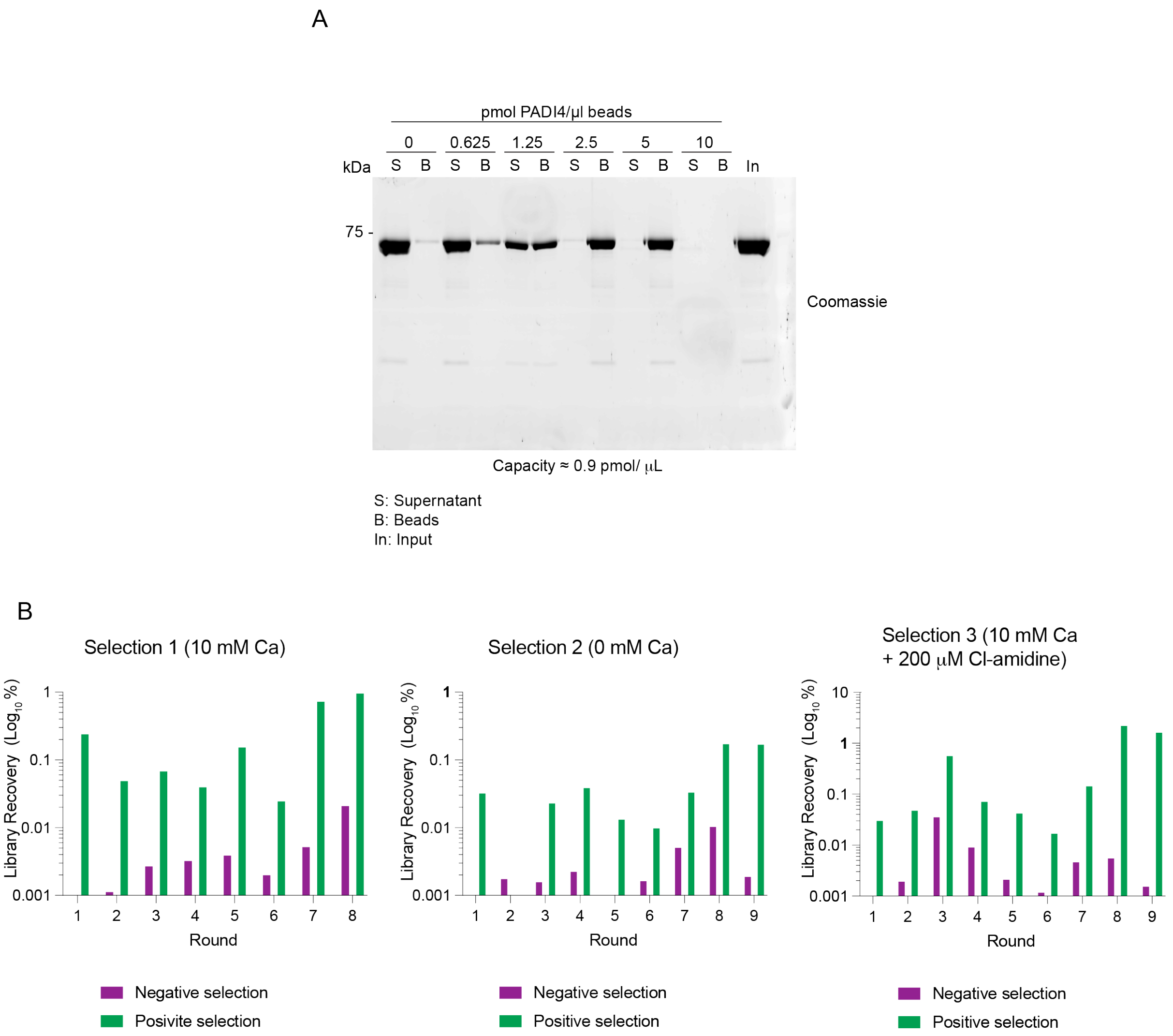

Supplementary Figure 1. **RaPID selections with hPADI4. A.** Determination of Avi-His-PADI4 loading level on streptavidin beads. **B.** RaPID selection recoveries. Violet bars and green bars represent the percentage of the input RaPID library recovered after affinity panning against streptavidin beads or streptavidin beads coated with biotinylated PADI4 respectively.

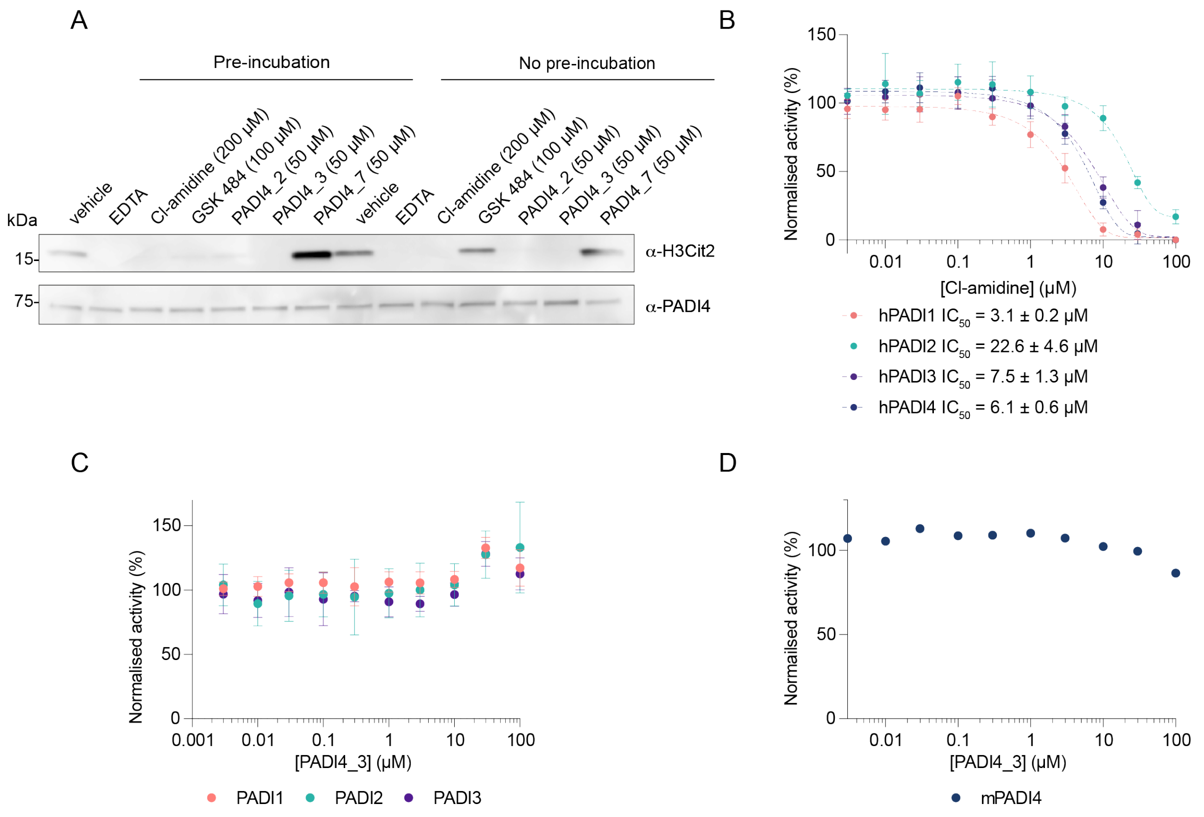

Supplementary Figure 2. PADI4_3 is a potent and selective hPADI4 inhibitor. **A.** Immunoblot analysis for citrullinated R2 on histone H3 (H3Cit2), as a measure of PADI4 activity, in hPADI4-stable mES cells. Cell extracts were incubated with indicated PADI inhibitors either 20 mins prior (pre-incubation) or concurrent with the addition of calcium (no pre-incubation). Cellular PADI4 serves as a loading control. **B.** Inhibition of hPADIs by Cl-amidine. COLDER assays of PADI family members with different concentrations of Cl-amidine and 10 mM $CaCl_2$. Data is normalised to activity of each PADI in the presence of 0.1% DMSO vehicle. Data shows mean ± SEM of three independent replicates. Each independent replicate was performed in triplicate. **C.** PADI4_**3** does not inhibit PADI1, PADI2 or PADI3. COLDER assays with PADI family members with different concentrations of PADI4_**3** and 10 mM $CaCl_2$. Data is normalised to activity of each PADI in the presence of 0.1% DMSO vehicle. Data shows mean ± SEM of three independent replicates. Each independent replicate was performed in triplicate. **D.** COLDER assay of mPADI4 with different concentrations of PADI4_**3** and 10 mM $CaCl_2$. Data shows mean ± SEM of three independent replicates. Each independent replicate was performed in triplicate.

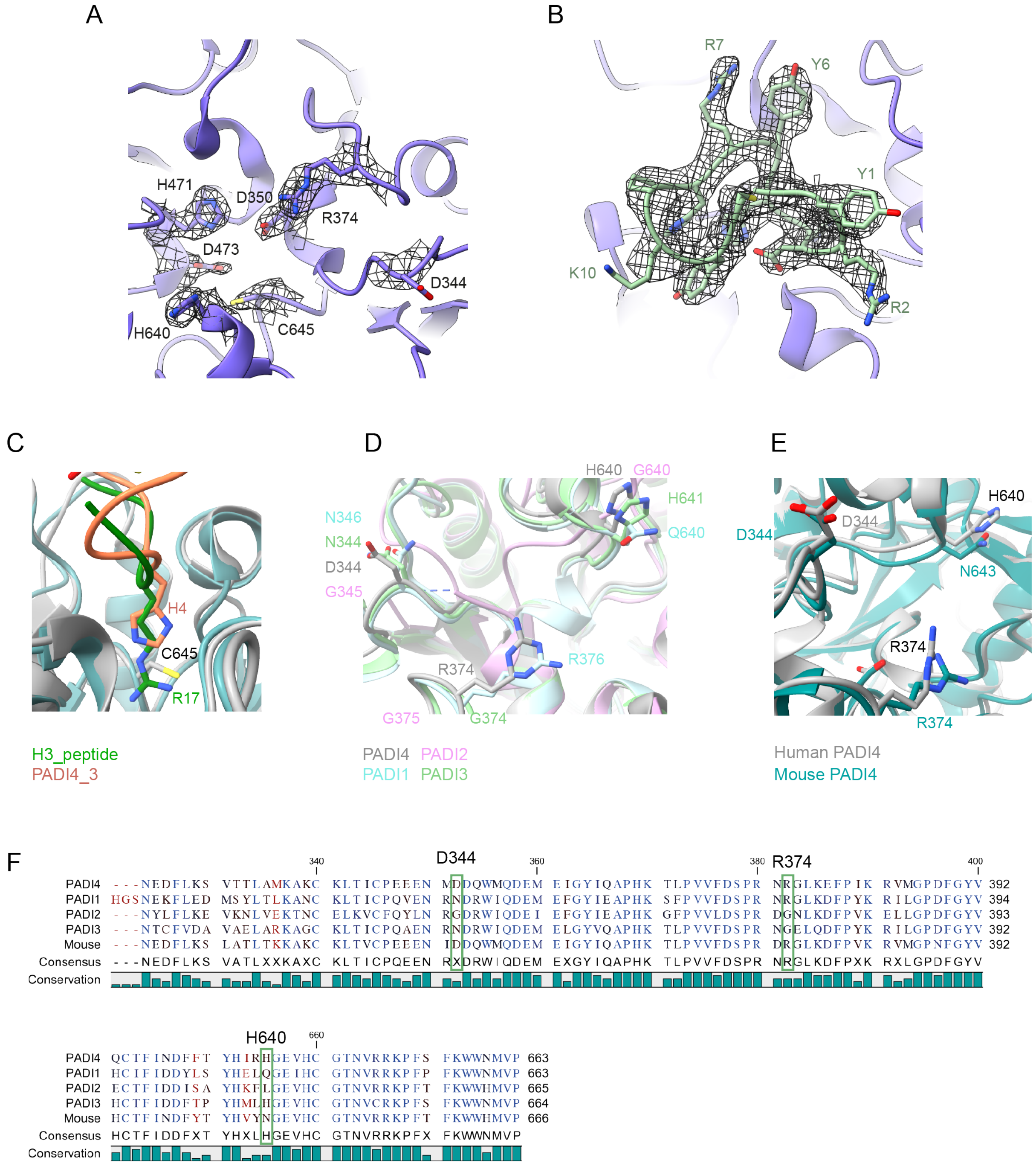

Supplementary Figure 3. **PADI4_3 binds to the active site of PADI4 and is selective for human PADI4. A.** PADI4 map showing electron density of significant amino acids involved in PADI4_**3** binding. **B.** Electron density of PADI4_**3**. **C.** Alignment of cryoEM structure of PADI4 bound to PADI4_**3** with X-ray crystal structure of PADI4 bound to an H3 peptide with R17 occupying the active site of PADI4 (PDB: 2DEX). PADI4_**3** H4 binds in the same pocket as R17. **D.** Alignment of human PADI4 (grey) with other human PADI isozymes. PADI1: AlphaFold model (cyan), PADI2 PDB: 4N2B (pink), PADI3: AlphaFold model (green). Side chains of residues important for interactions with PADI4_**3** are shown as sticks. **E.** Alignment

of human PADI4 (grey) with its mouse ortholog (teal) (mouse PADI4: AlphaFold model). Side chains of residues important for interactions with PADI4_**3** are shown as sticks. **F.** Sequence alignment of human PADI4 with other human PADI enzymes and mouse PADI4. Residues highlighted in green boxes are involved in the binding of PADI4_**3** peptide and are not conserved.

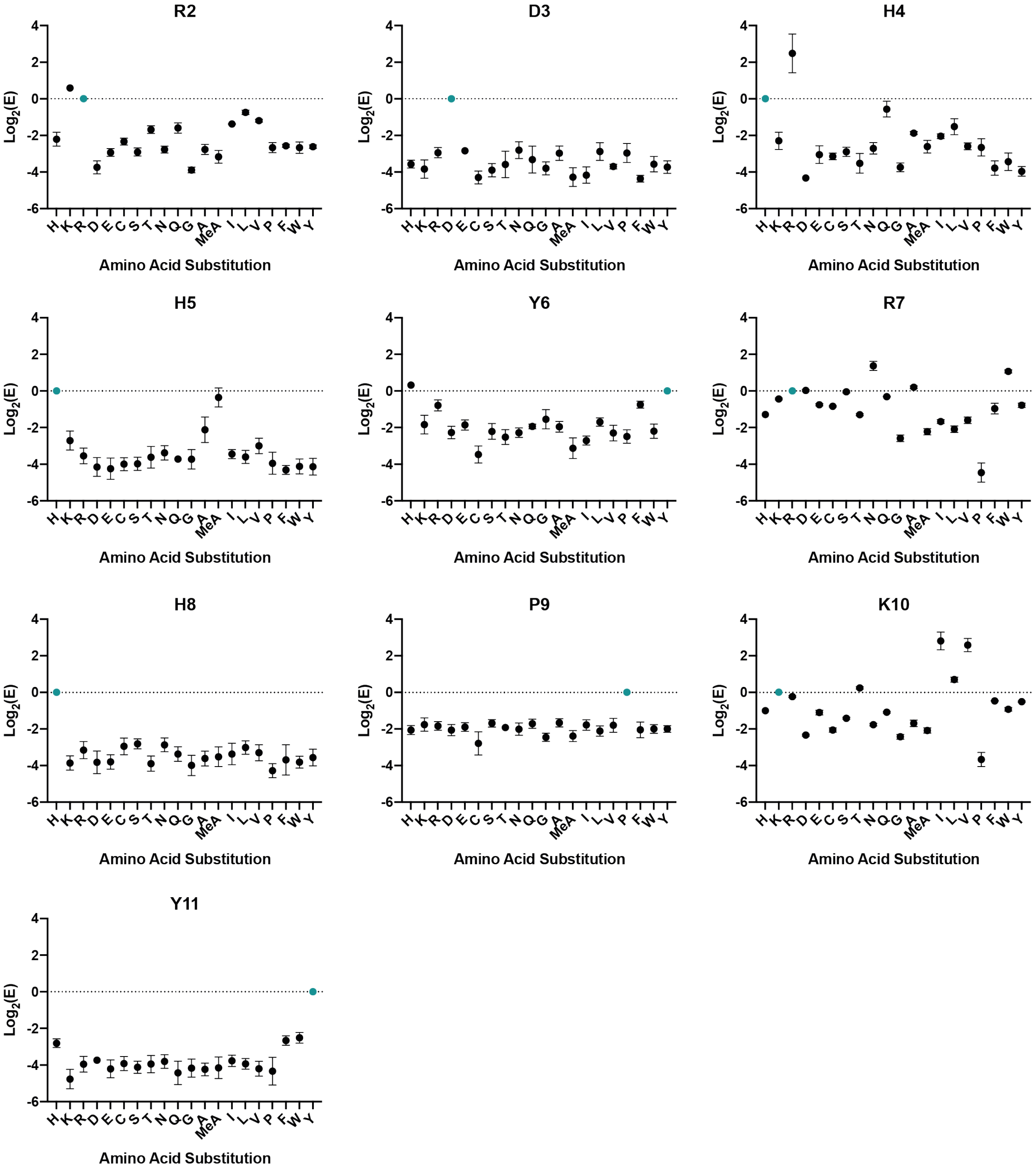

Supplementary Figure 4. Deep mutational scanning of PADI4_3. $Log_2(E)$ scores are plotted for each amino acid substitution, for every position varied in the PADI4_**3** sequence. Data are plotted as the mean ± standard deviation of three independent single round selections against immobilised PADI4.

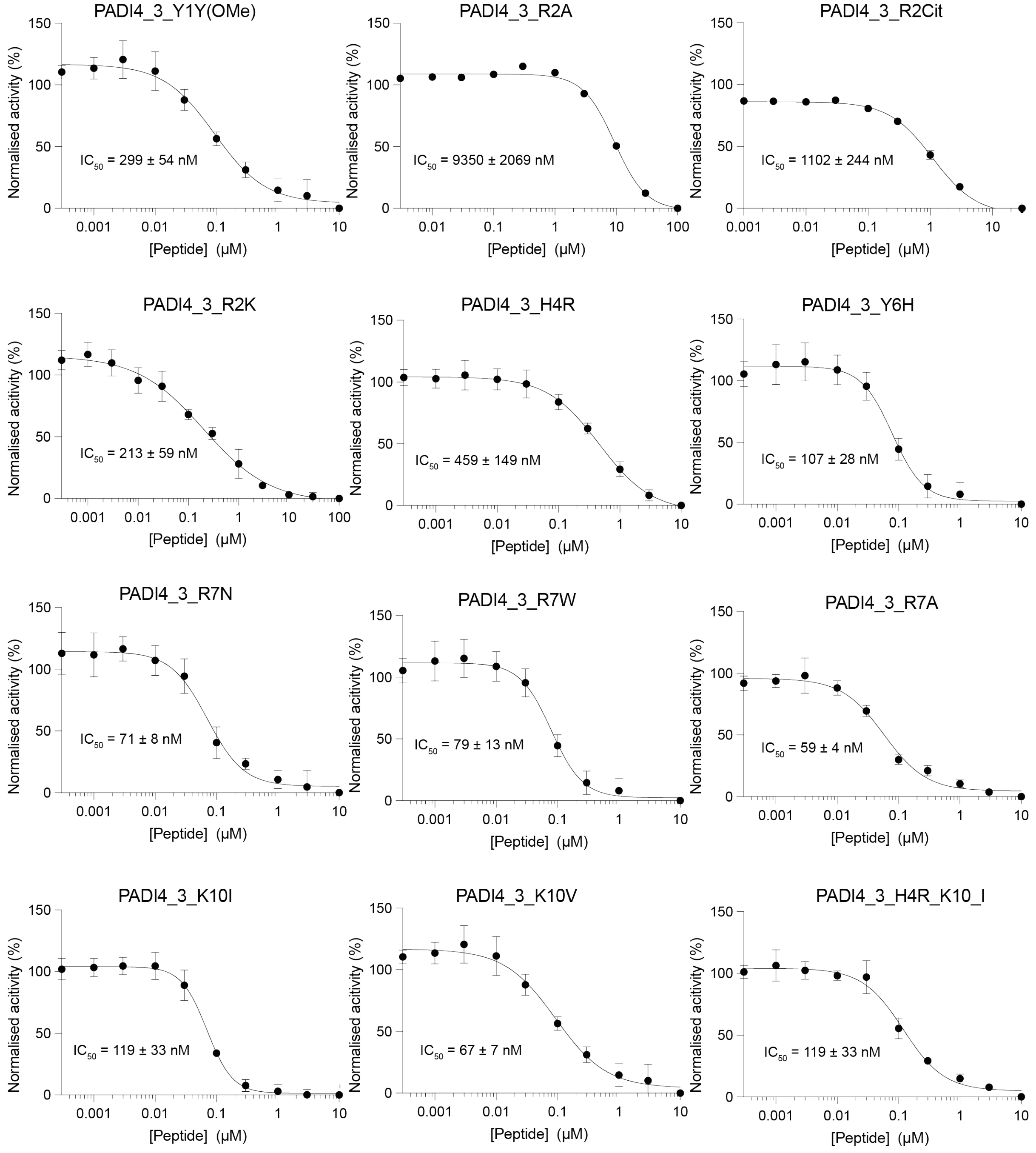

Supplementary Figure 5. **Inhibition of hPADI4 by PADI4_3 analogues.** hPADI4 inhibition by different concentrations of peptides (10-0.0003 μM) and 10 mM $CaCl_2$ measured by COLDER assay. Data is normalised to activity of PADI4 in the presence of 0.1% DMSO vehicle. Data shows mean ± SEM of three independent replicates. Each independent replicate was performed in triplicate.

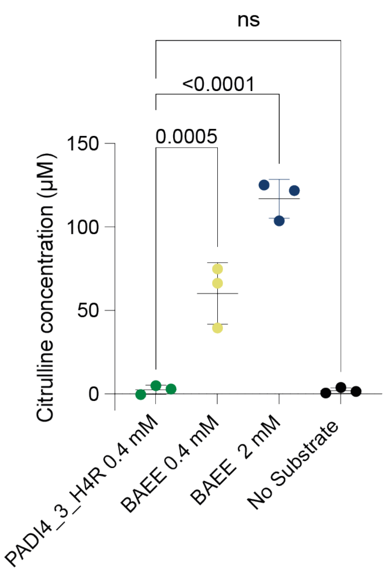

Supplementary Figure 6. PADI4_3_H4R is not a PADI4 substrate. COLDER assay performed with 0.4 mM PADI4_**3_H4R**, BAEE was used as positive control. Data represents mean ± SEM of three independent replicates. Each replicate was done in triplicate. A two-way ANOVA was carried out to determine if the differences among means were significant.

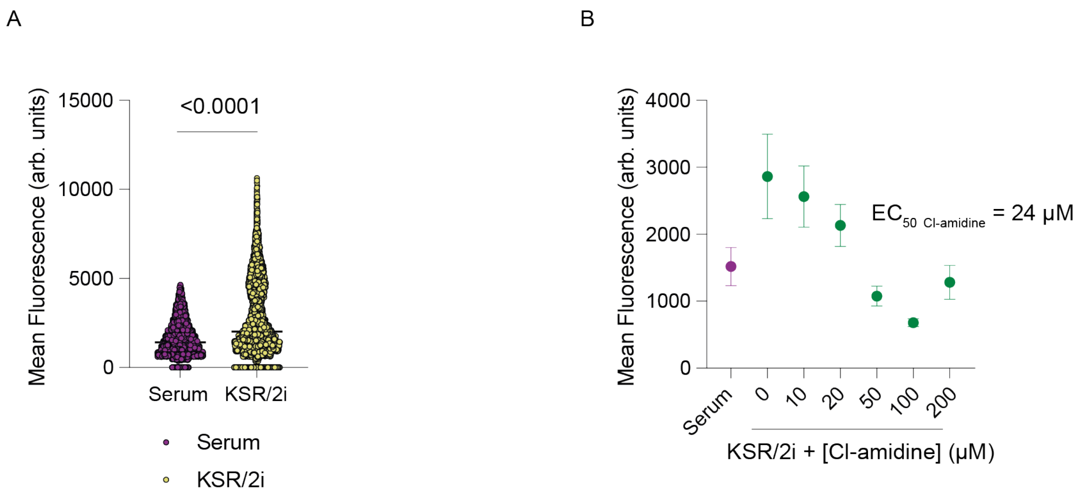

Supplementary Figure 7. Validation of high content microscopy method. A. High content imaging-based quantification of mean H3Cit immunofluorescence intensity in hPADI4-stable mES cells grown in Serum or KSR/2i for 3 h. Each data point represents the mean H3Cit intensity per cell. Cells from three technical replicates and three biological replicates are included. A two-tailed t-test was carried out to determine if the differences among means were significantly different. **B.** High content imaging-based quantification of mean H3Cit immunofluorescence intensity in hPADI4-stable mES cells stimulated with KSR/2i for 3 h, in the presence of increasing concentrations of Cl-amidine. Each data point represents the mean H3Cit intensity per conditions from three technical replicates.

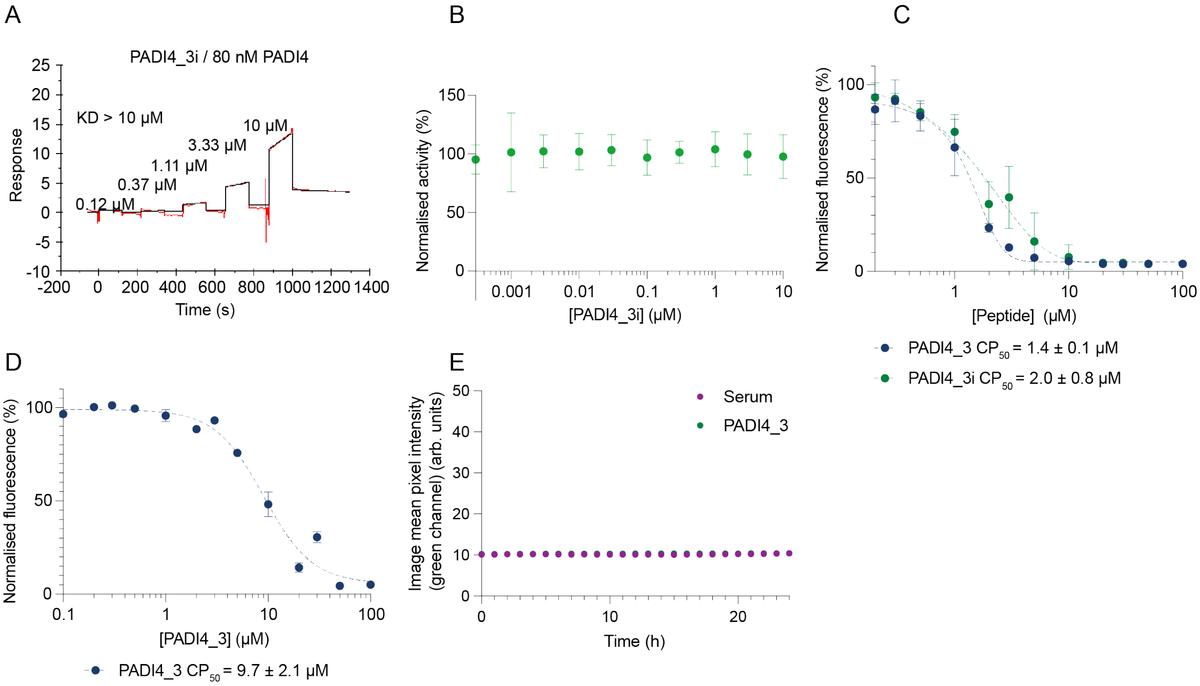

Supplementary Figure 8. PADI4_3i does not inhibit PADI4 and PADI4_3 and PADI4_3i are cell permeable and non-toxic. A. PADI4_**3i** does not bind to PADI4. Binding kinetics between PADI4 and PADI4_**3i** measured by SPR. A representative experiment is shown. This experiment was performed three times with similar results. **B.** PADI4_**3i** does not inhibit PADI4. COLDER assay with PADI4_**3i** at different concentrations (10 - 0.0003 µM) and 10 mM $CaCl_2$. Data are normalised to activity of PADI4 in the presence of 0.1% DMSO vehicle. Data show mean ± SEM of three independent replicates. Each replicate was performed in triplicate. **C.** PADI4_3 and PADI4_**3i** enter cells. CAPA assay with PADI4_**3** and PADI4_**3i**. Data show mean ± SEM of three different experiments. Each replicate was performed in triplicate. Data are normalised to cells with no peptide treated with TMR dye (Promega) (100 %) and cells with no peptide and no dye (0 %). **D.** CAPA assay with PADI4_**3** at 4 °C demonstrates that PADI4_**3** enters the cells via active cellular transport. Data show mean ± SEM of two independent experiments. Each replicate was performed in triplicate. Data are normalised as in **C**. **E.** Assessment of cytotoxicity by live cell imaging with Incucyte® Cytotox Green Dye, as a measure for cell death. hPADI4 expressing mES cells treated with 1 µM PADI4_**3** or DMSO vehicle (0.1%), and imaged for 24h.

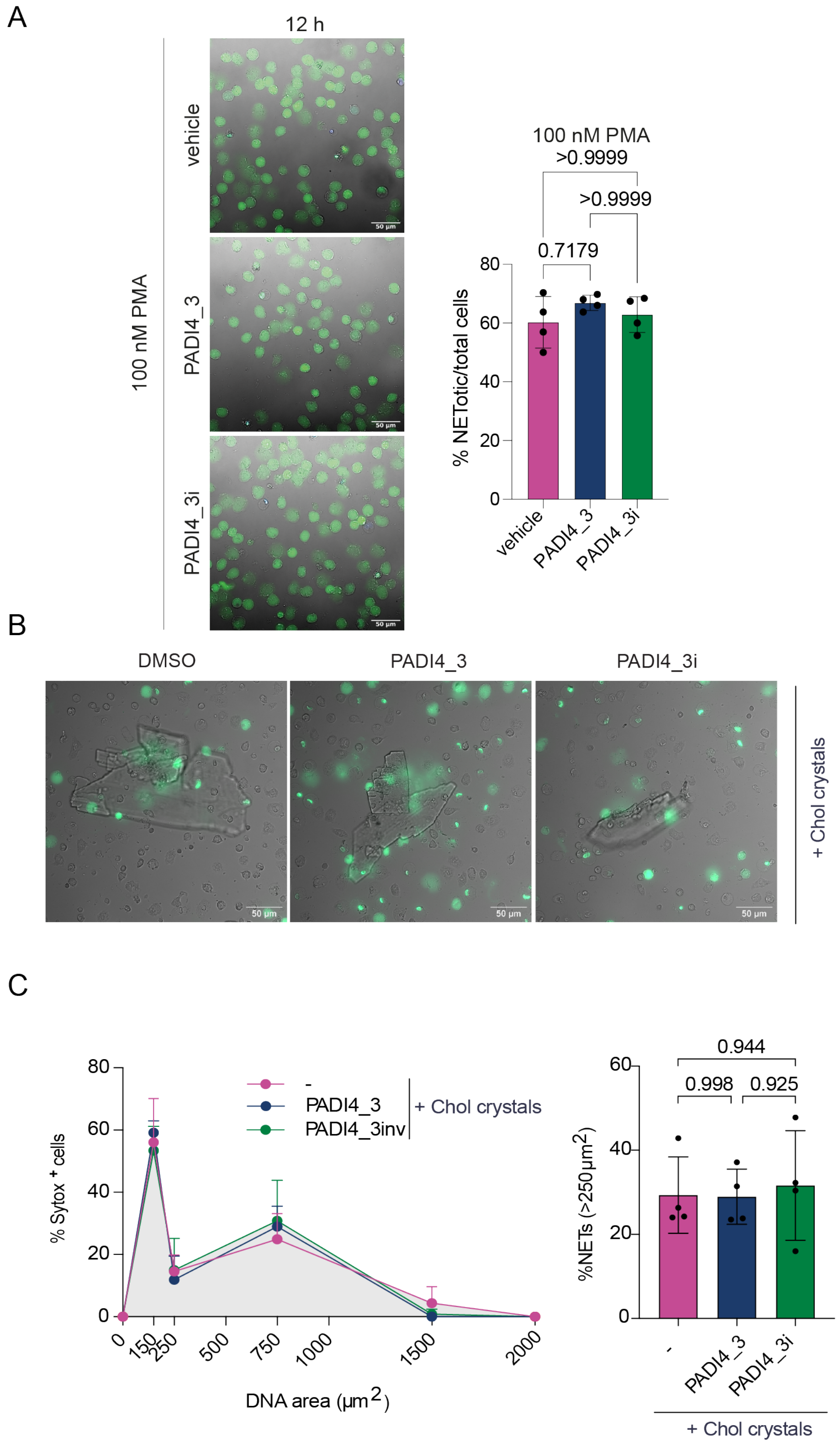

Supplementary Figure 9. PADI4_3 does not affect NETosis in human neutrophils stimulated with PMA or cholesterol crystals. A. Bright field micrograph of human neutrophils pre-incubated with vehicle, PAD4_**3** or PAD4_**3i** peptides (50 µM), imaged 12h post-stimulation with PMA (left) and the corresponding quantification of the percentage of

NETotic cells over total cells (right). Data represents mean ± SD of four replicates. A Kruskal-Wallis test was carried out to determine if the differences in means are significantly different. p-values were adjusted using the Dunn’s correction **B.** Human neutrophils were pre-incubated with DMSO vehicle (0.5%) or 50 µM PAD4_**3** or PAD4_**3i** and the membrane-impermeable dye Sytox-green before stimulation with 0.1 mg/ml cholesterol crystals. Images were acquired by time-lapse fluoresence microscopy, with images at 8h shown. **C.** Quantification of (B). (left) DNA area was measured by Sytox-green signal and areas were distributed into bins of increasing area sizes and plotted as a percentage of Sytox-green positive (dead) cells. Data shown are means +/- SD from one representative experiment. (right) Percentage of NETotic cells in each treatment condition.

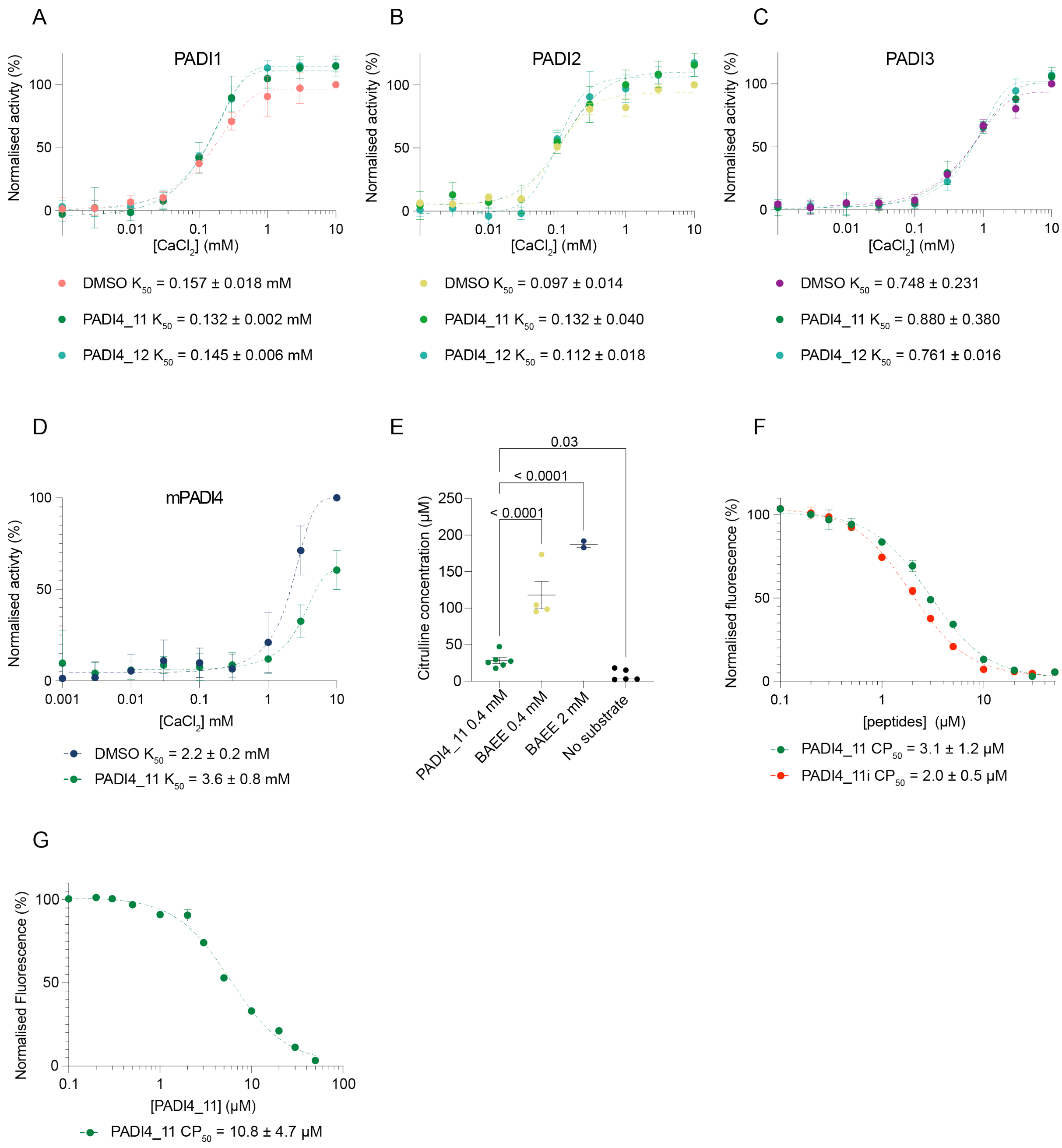

Supplementary Figure 10. **PADI4_11 is selective for hPADI4, may be a very poor PADI4 substrate and is cell permeable. A-D.** PADI4_**11** and PADI4_**12** do not activate hPADI1 (A), hPADI2 (B), hPADI3 (C) or mPADI4 (D). COLDER assays were performed in presence or absence of PADI4_**11** or PADI4_**12** at 30 µM and different concentrations of $CaCl_2$. K_{50Ca2+} is the concentration of $CaCl_2$ that yields half maximal PADI activity. Data represent mean ± SEM of three independent replicates. Each replicate was performed in triplicate. Data were normalised against the activity of each PADI in the presence of 0.1% DMSO vehicle and 10 mM $CaCl_2$. **E.** PADI4_**11** may be a very poor PADI4 substrate. COLDER assay performed with 0.4 mM PADI4_**11** and BAEE was used as positive control. Data represents mean ± SEM of three independent replicates. . A two-way ANOVA was carried out to determine if the

differences among means were significant. Each replicate was done in triplicate. **F.** PADI4_**11** and PADI4_**11i** are cell permeable. CAPA assay with PADI4_**11** and PADI4_**11i**. Data show mean ± SEM of three independent experiments performed in triplicate. Data are normalised with cells with no peptide treated with TMR dye (Promega) (100 %) and cells with no peptide and no dye (0 %). **G.** CAPA assay with PADI4_**11** at 4 °C demonstrates that PADI4_**11** enters the cells via active cellular transport. Data show mean ± SEM of three independent experiments performed in triplicate. Data was normalised as in **F**.

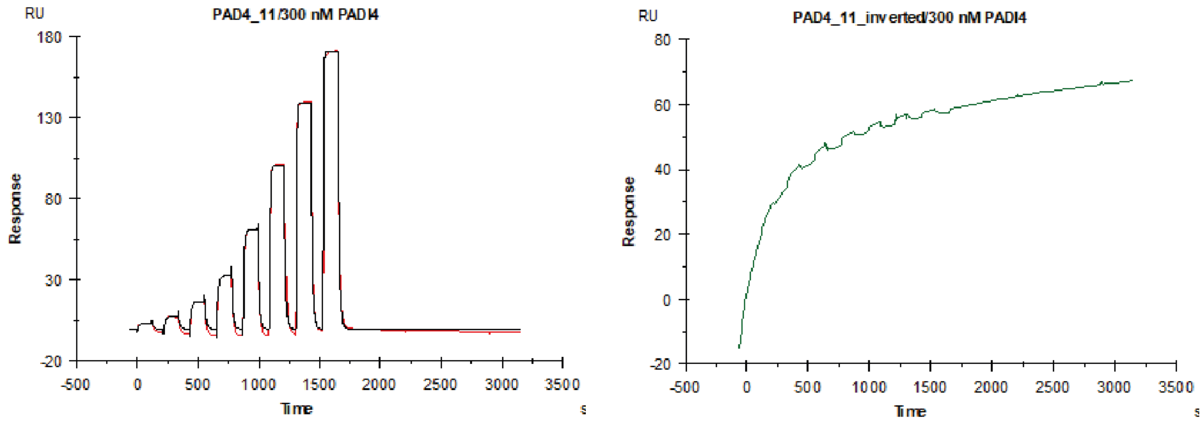

Supplementary 11. PADI4_11i does not bind to PADI4. SPR analysis of hPADI4 with either PADI4_11 or PADI4_11i peptides. Experiments were performed in triplicate and a representative experiment is shown.

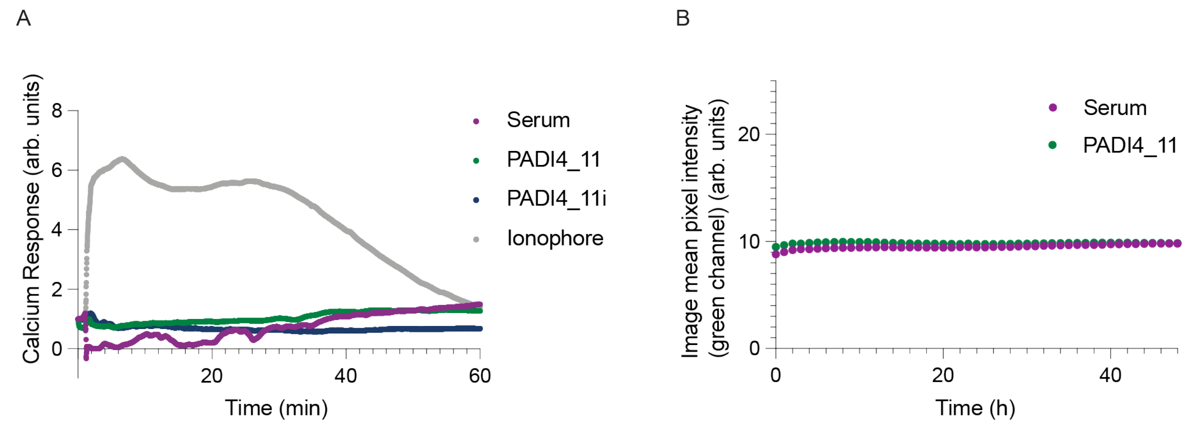

Supplementary 12. Time course of calcium influx. A. Calcium influx into cells over the course of 60 minutes, as measured by Calbryte intensity, after treatment with 25 μM PADI4_**11** or PADI4_**11i**. Calcium ionophore (10 μM) used as a positive control for calcium influx. **B.** Assessment of cytotoxicity by live cell imaging with Incucyte® Cytotox Green Dye, as a read-out for cell death. hPADI4 expressing mES cells treated with 25μM PADI4_**11** or DMSO vehicle (0.1%), and imaged for 48 h.

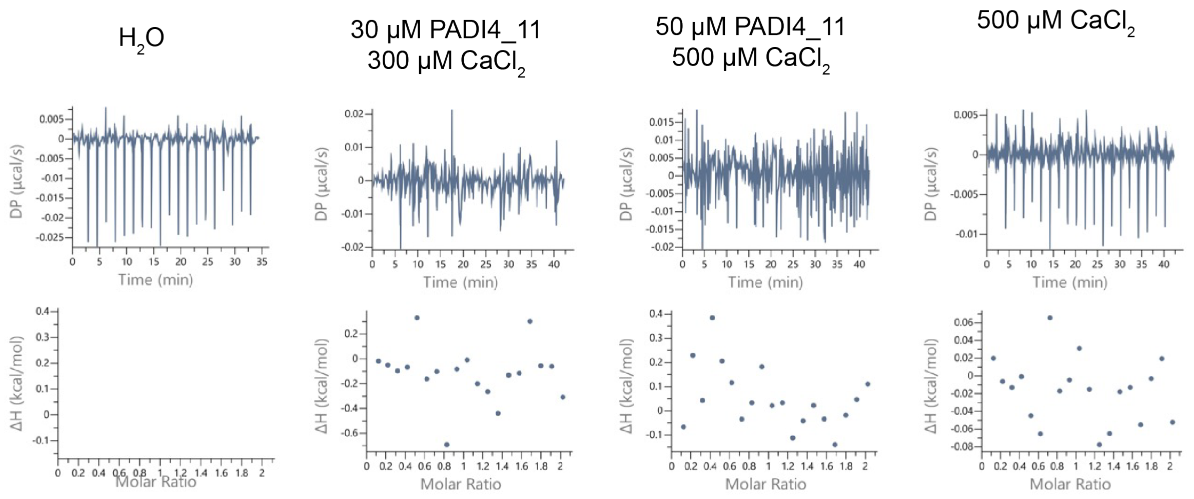

Supplementary Figure 13. PADI4_11 does not bind to calcium. Isothermal Titration Calorimetry between PADI4_**11** peptide (30 and 50 µM) and $CaCl_2$ (300 and 500 µM).

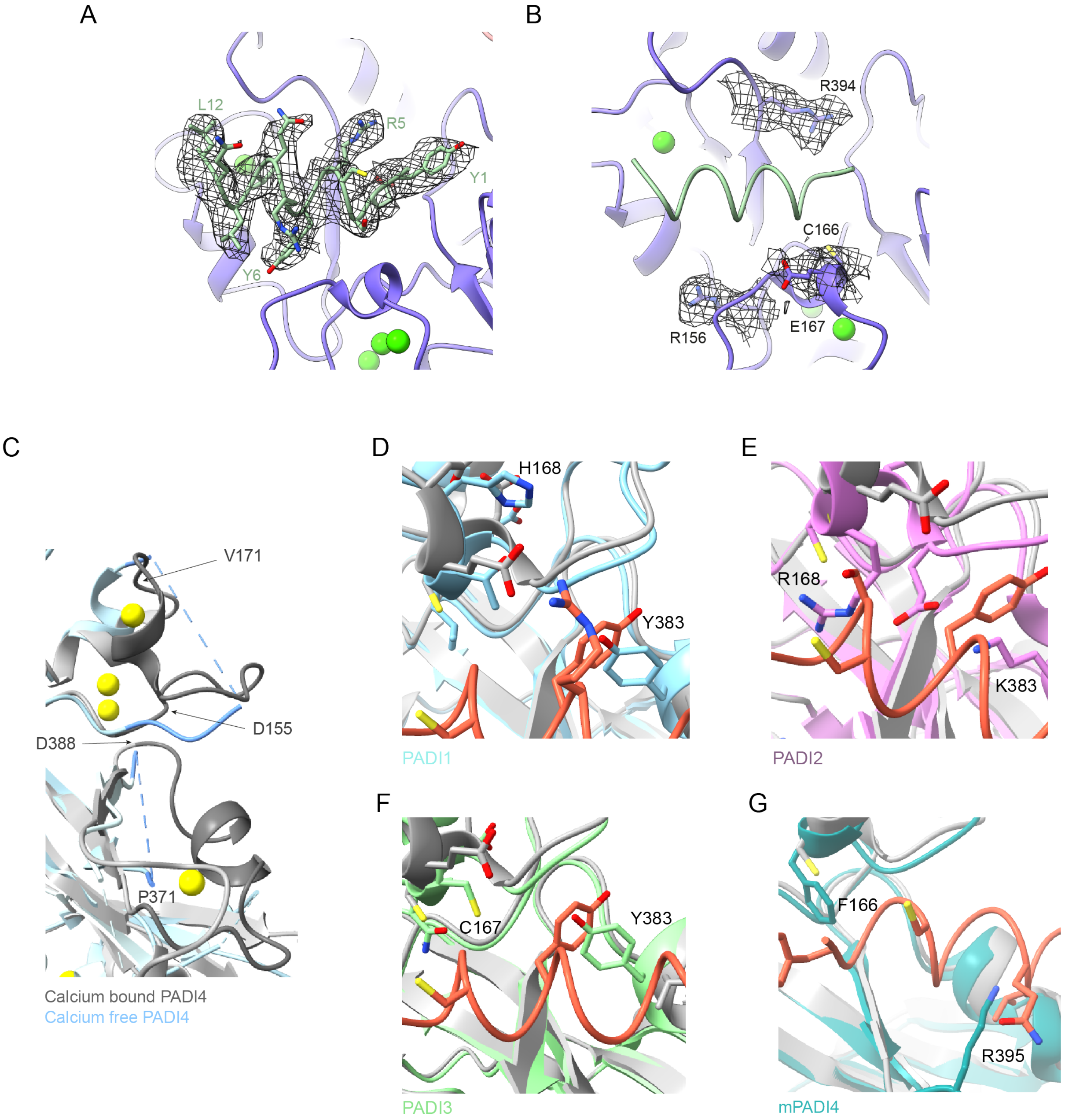

Supplementary Figure 14. PADI4_11 binds selectively to hPADI4. A. Electron density of PADI4_**11. B.** Electron density of significant PADI4 amino acids involved in the binding with PADI4_**11**. **C.** Alignment of PADI4 bound to calcium (grey) and calcium free PADI4 (PBD ID: 1WD8) (blue). Loops involved in the binding of PADI4_**11** peptide are shown in dark grey. **D.** Structure alignment of PADI1 AlphaFold model (cyan) with hPADI4 in complex with peptide PADI4_11. **E.** Structure alignment of PADI2 (PDB: 4N2B, magenta) with hPADI4 in complex with peptide PADI4_**11**. **F.** Structure alignment of PADI3 AlphaFold model (light green) with hPADI4 in complex with peptide PADI4_**11**. **G.** Structure alignment of mouse PADI4 AlphaFold model (teal) with hPADI4 in complex with peptide PADI4_**11**. Protein residues predicted to clash with PADI4_**11** are highlighted as sticks.

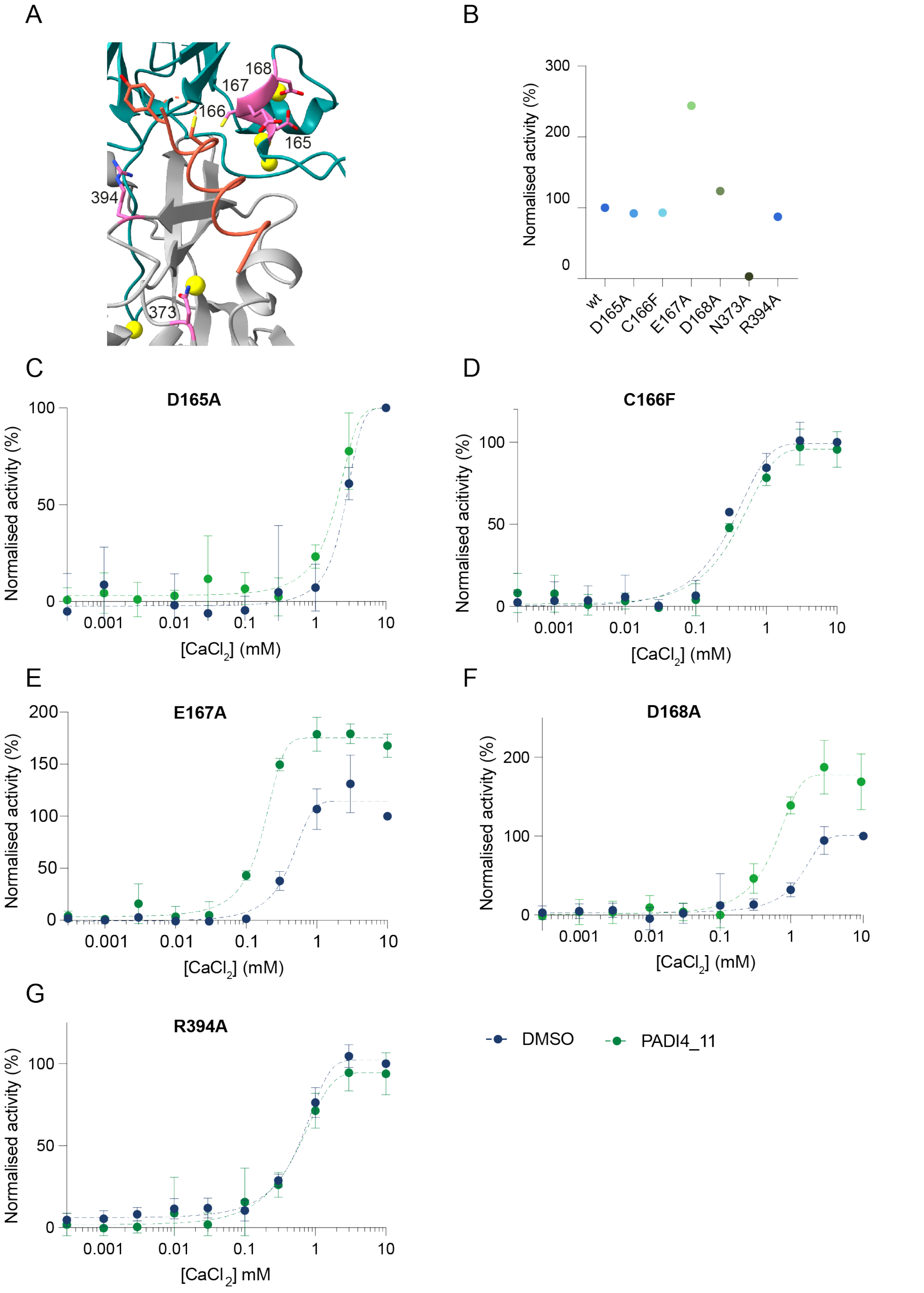

Supplementary Figure 15. PADI4_11 cannot activate some hPADI4 variants. A. View from a PADI4 cryoEM structure with PADI4_**11** (orange). Residues involved in the binding that were mutated for following experiments are shown in pink. **B.** Different PADI4 variants have

different activities. Activity of each PADI4 variant was measured by COLDER assay at 10 mM $CaCl_2$ following 30 min incubation at RT. Activity was normalised against the activity of wild-type PADI4. **C-G**. PADI4_**11** is not able to activate some PADI4 variants. COLDER assays were performed in presence or absence of PADI4_**11** at 30 μM and different concentrations of $CaCl_2$. Activity was normalised against the activity of each variant at 10 mM $CaCl_2$ in absence of peptide. Data represents mean ± SEM of two independent replicates. Each replicate was performed in triplicate.

A

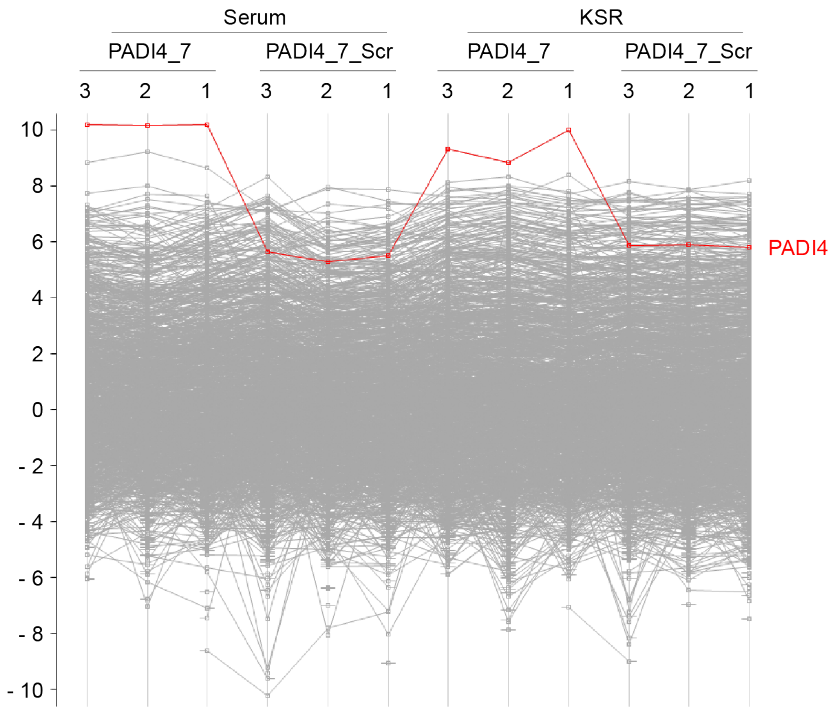

B

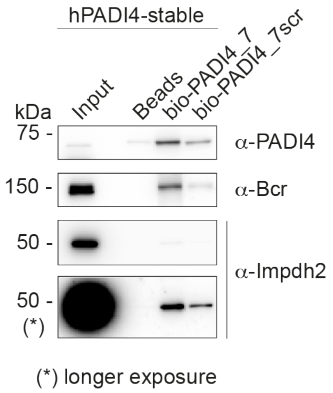

Supplementary Figure 16. Specific enrichment of PADI4 by bio-PADI4_7 and confirmation of interactors by immunoblot. A. Enrichment profile for PADI4 compared to all other identified proteins across all pull-down samples, as determined by Mass Spectrometric analysis. **B.** Immunoblot analysis of bio-PADI4_**7** and bio-PADI4_**7scr** pull-downs for PADI4, Bcr and Impdh2.

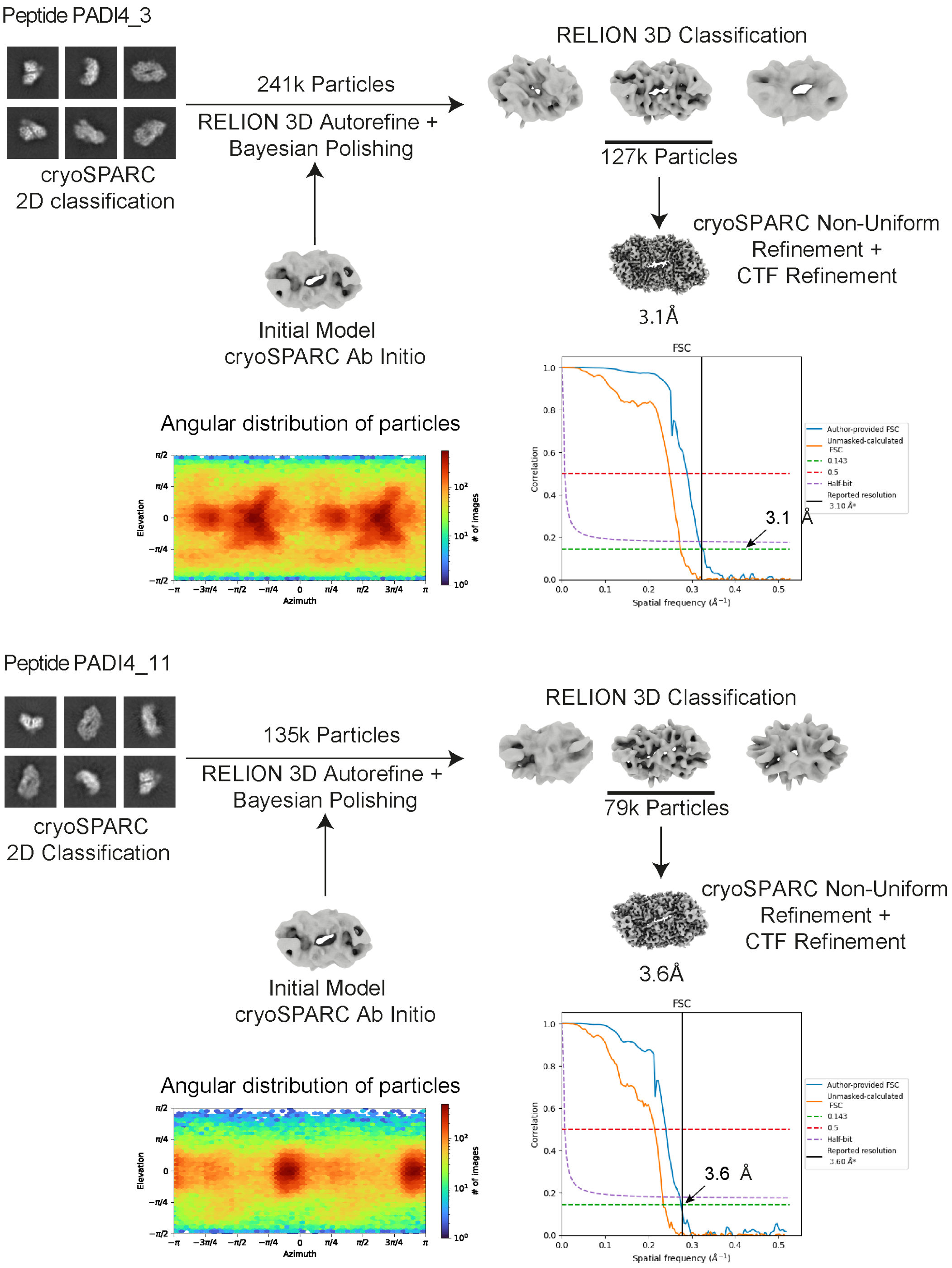

Supplementary Figure 17. Cryo-electron microscopy data processing pipeline for the structures of PADI4 in complex with PADI4_**3** (top) and PADI4 in complex with PADI4_**11** (bottom).

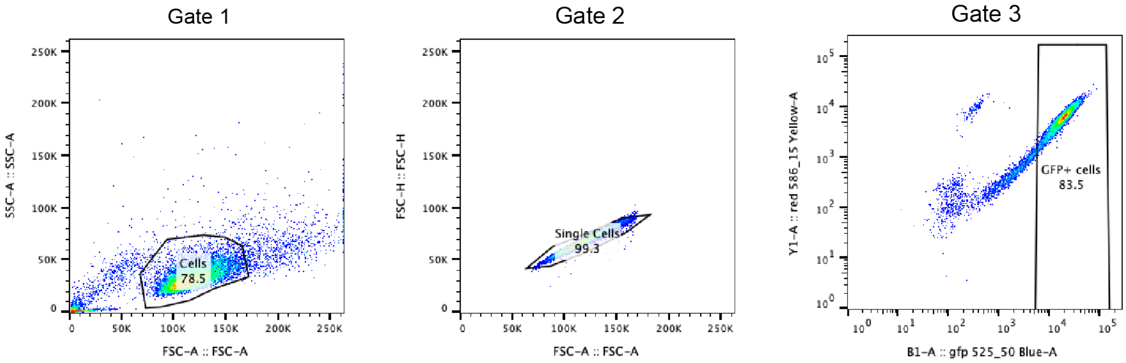

Supplementary Figure 18. Gating workflow followed to analyse Chloroalkane Penetration Assay (CAPA).

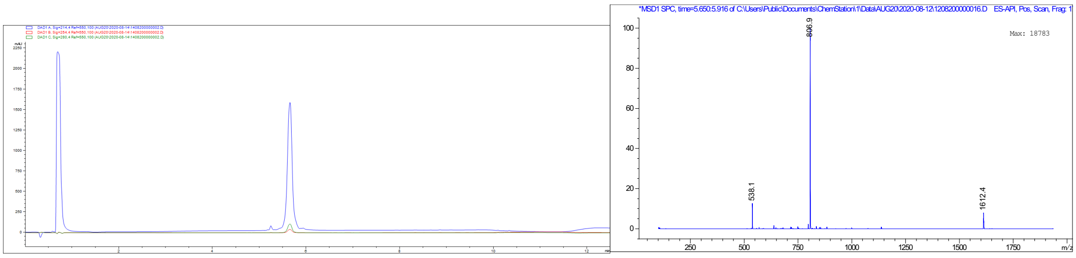

Supplementary Figure 19. PADI4_2 Left: HPLC trace, showing A214 in blue, A254 in red and A280 in green. Right: ESI-MS trace

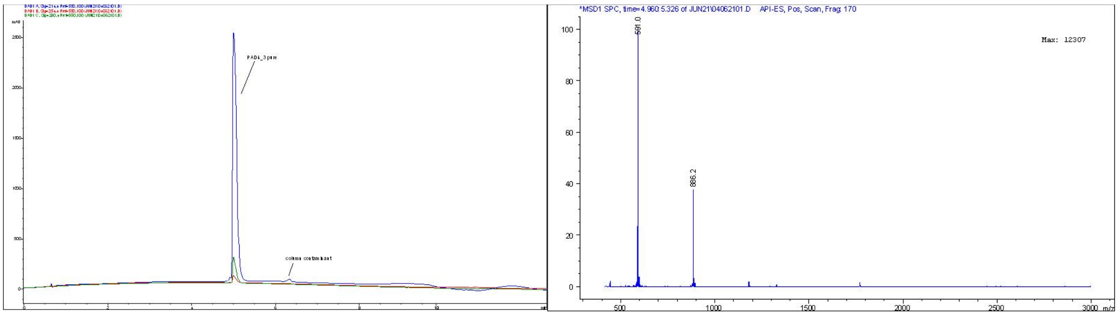

Supplementary Figure 20. PADI4_3 Left: HPLC trace, showing A214 in blue, A254 in red and A280 in green. Right: ESI-MS trace

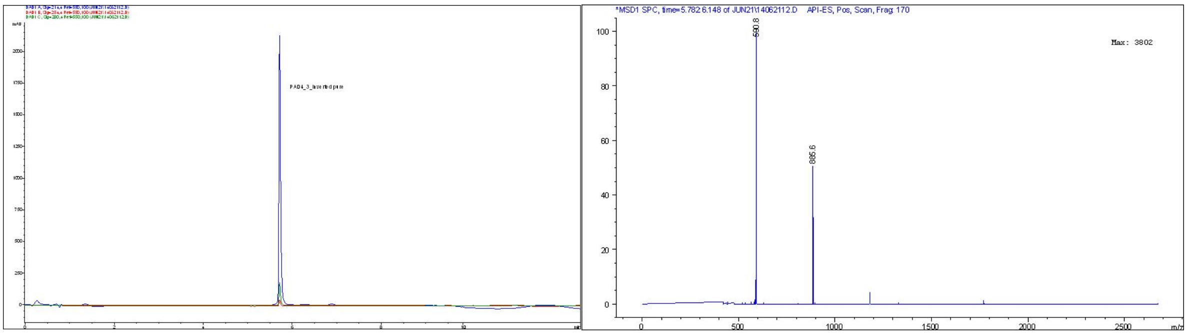

Supplementary Figure 21. PADI4_3i Left: HPLC trace, showing A214 in blue, A254 in red and A280 in green. Right: ESI-MS trace

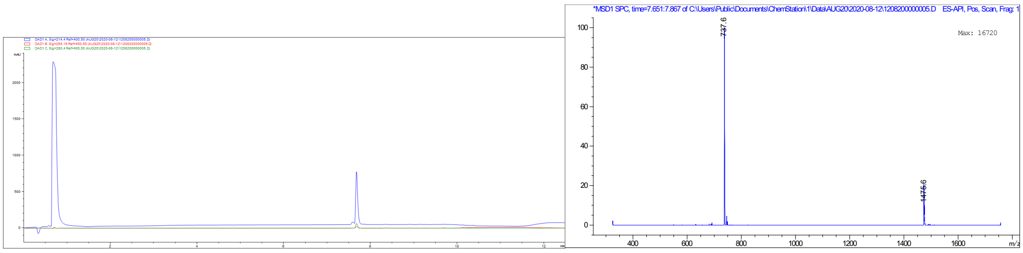

Supplementary Figure 22. PADI4_4 Left: HPLC trace, showing A214 in blue, A254 in red and A280 in green. Right: ESI-MS trace

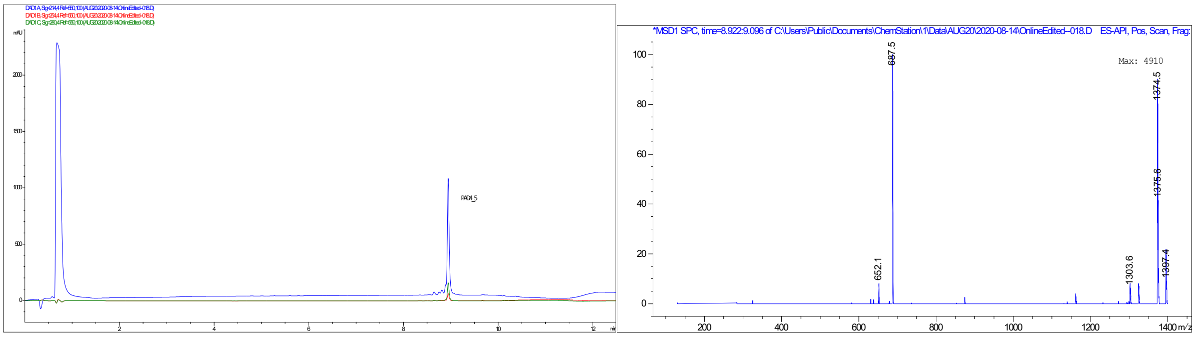

Supplementary Figure 23. PADI4_5 Left: HPLC trace, showing A214 in blue, A254 in red and A280 in green. Right: ESI-MS trace

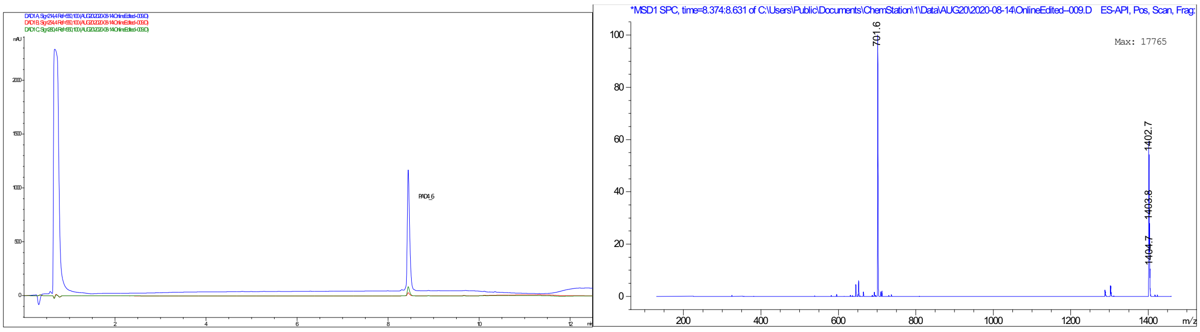

Supplementary Figure 24. PADI4_6 Left: HPLC trace, showing A214 in blue, A254 in red and A280 in green. Right: ESI-MS trace

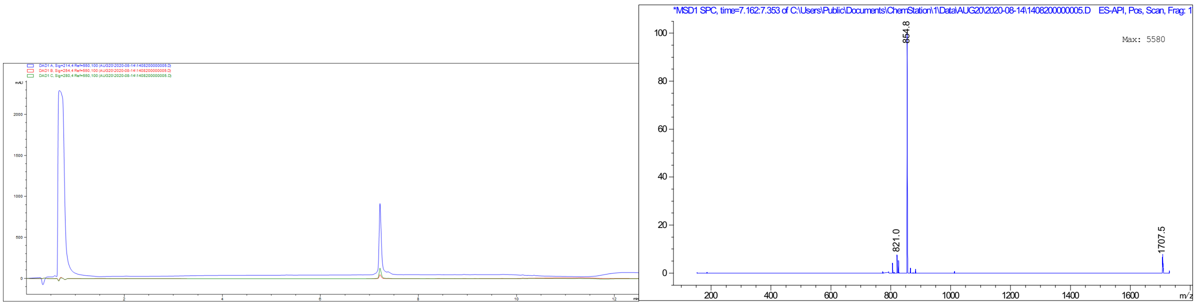

Supplementary Figure 25. PADI4_7 Left: HPLC trace, showing A214 in blue, A254 in red and A280 in green. Right: ESI-MS trace

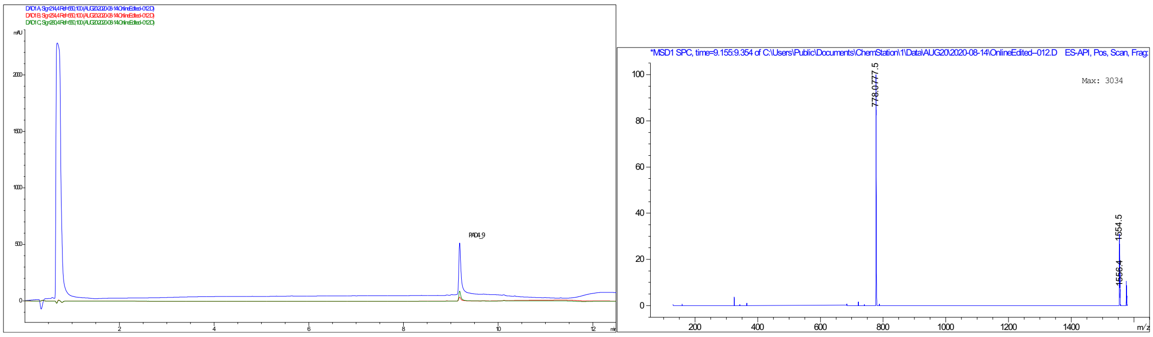

Supplementary Figure 26. PADI4_9 Left: HPLC trace, showing A214 in blue, A254 in red and A280 in green. Right: ESI-MS trace

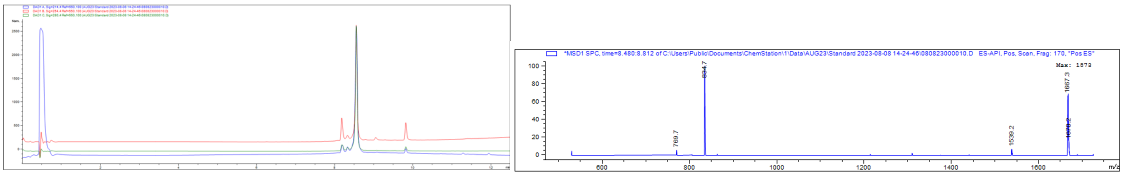

Supplementary Figure 27. PADI4_10 Top: HPLC trace, showing A214 in blue, A254 in red and A280 in green. Bottom: ESI-MS trace

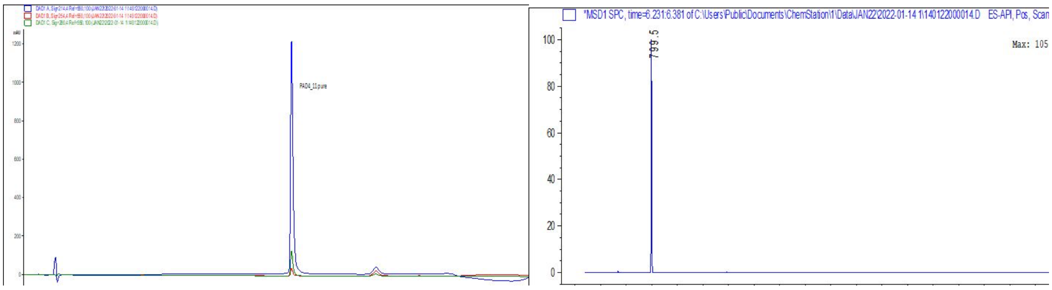

Supplementary Figure 28. PADI4_11 Left: HPLC trace, showing A214 in blue, A254 in red and A280 in green. Right: ESI-MS trace

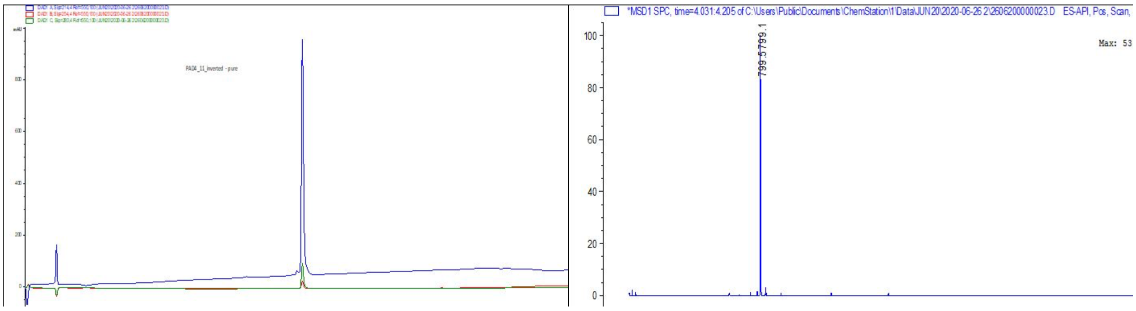

Supplementary Figure 29. PADI4_11i Left: HPLC trace, showing A214 in blue, A254 in red and A280 in green. Right: ESI-MS trace

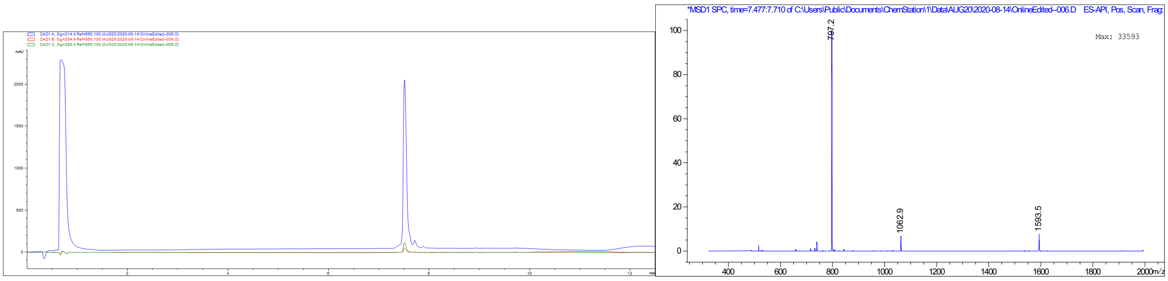

Supplementary Figure 30. PADI4_12 Left: HPLC trace, showing A214 in blue, A254 in red and A280 in green. Right: ESI-MS trace

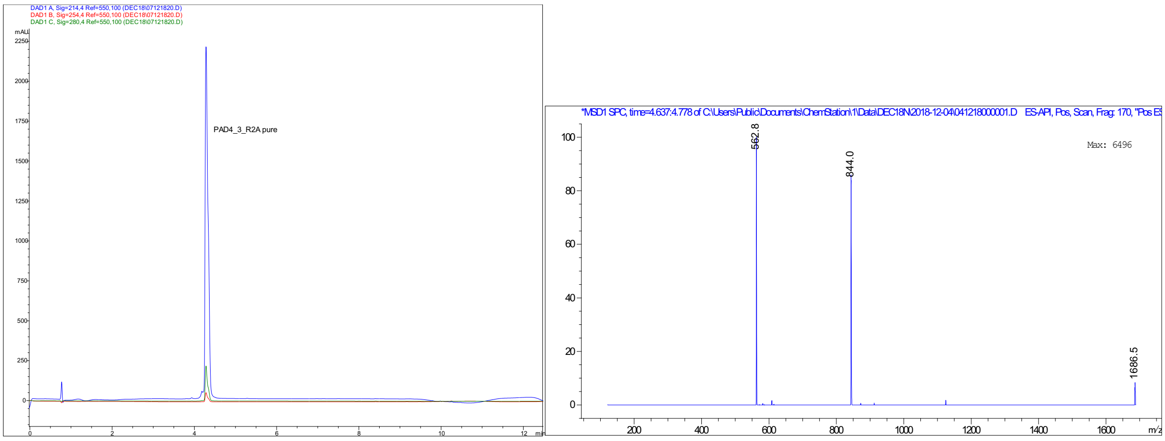

Supplementary Figure 31. PADI4_3_R2A Left: HPLC trace, showing A214 in blue, A254 in red and A280 in green. Right: ESI-MS trace of crude peptide

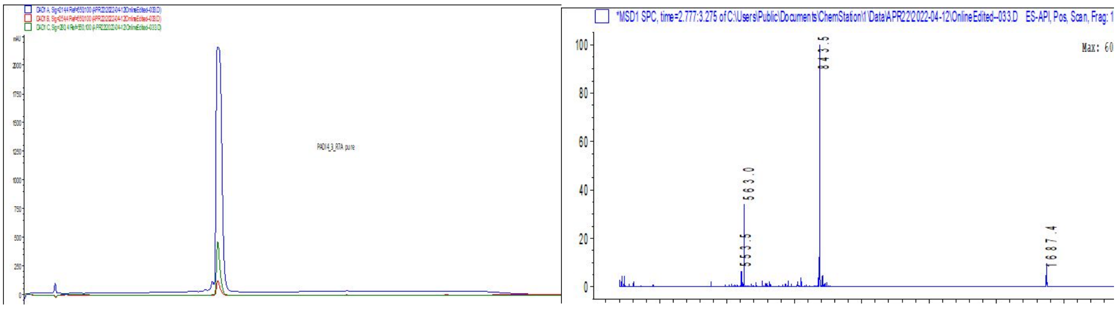

Supplementary Figure 32. PADI4_3_R7A Left: HPLC trace, showing A214 in blue, A254 in red and A280 in green. Right: ESI-MS trace

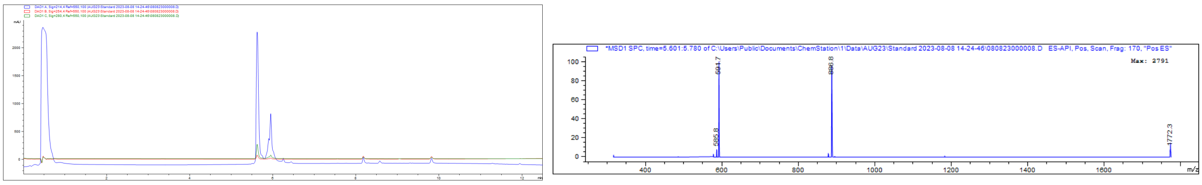

Supplementary Figure 33. PADI4_3_R2cit Top: HPLC trace, showing A214 in blue, A254 in red and A280 in green. Bottom: ESI-MS trace

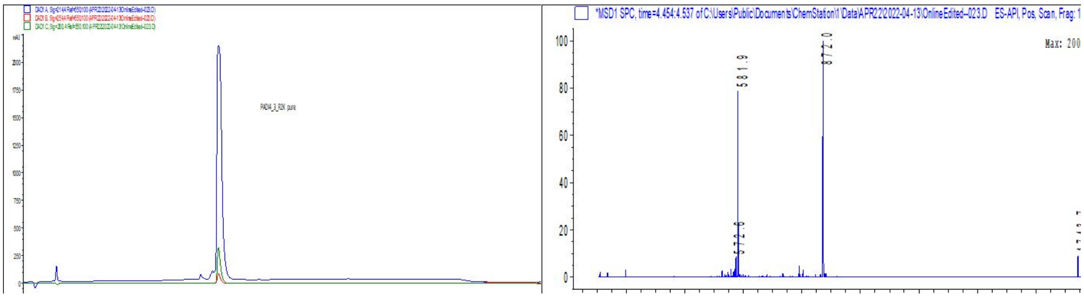

Supplementary Figure 34. PADI4_3_R2K Left: HPLC trace, showing A214 in blue, A254 in red and A280 in green. Right: ESI-MS trace

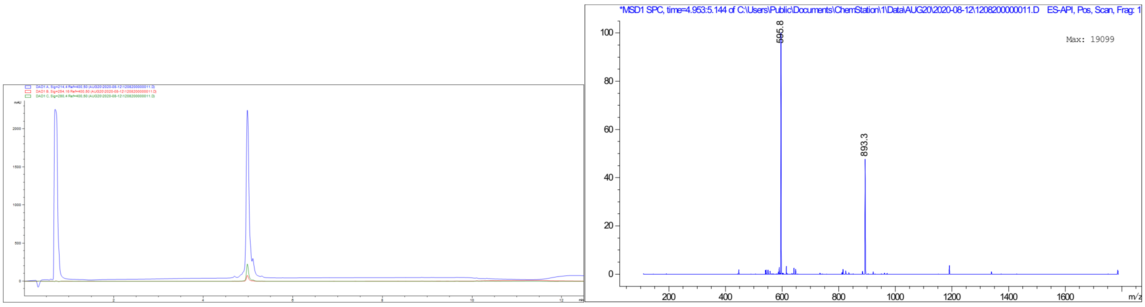

Supplementary Figure 35. PADI4_3_y1(OMe)Y Left: HPLC trace, showing A214 in blue, A254 in red and A280 in green. Right: ESI-MS trace

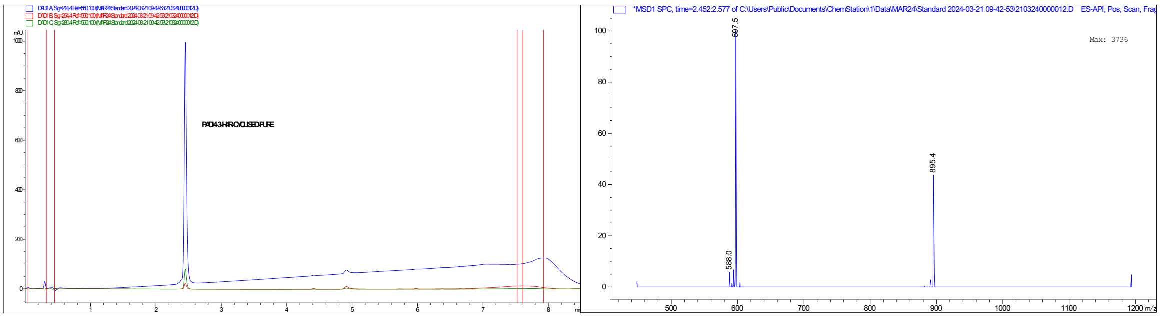

Supplementary Figure 36. PADI4_3_H4R Left: HPLC trace, showing A214 in blue, A254 in red and A280 in green. Right: ESI-MS trace

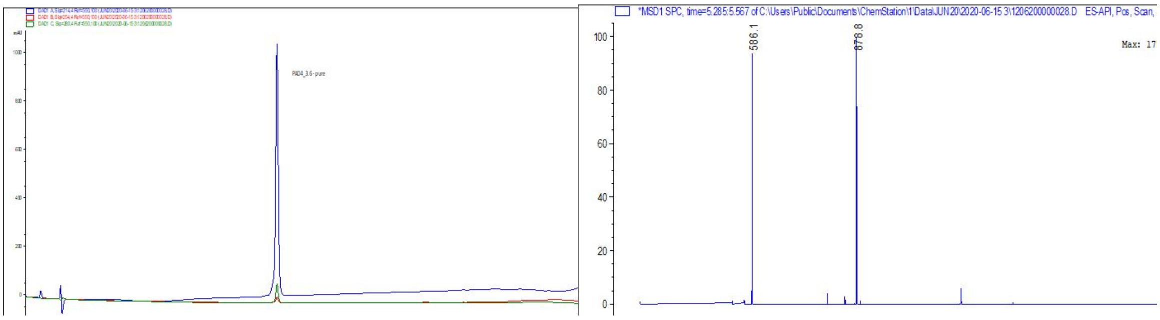

Supplementary Figure 37. PADI4_3_K10I Left: HPLC trace, showing A214 in blue, A254 in red and A280 in green. Right: ESI-MS trace

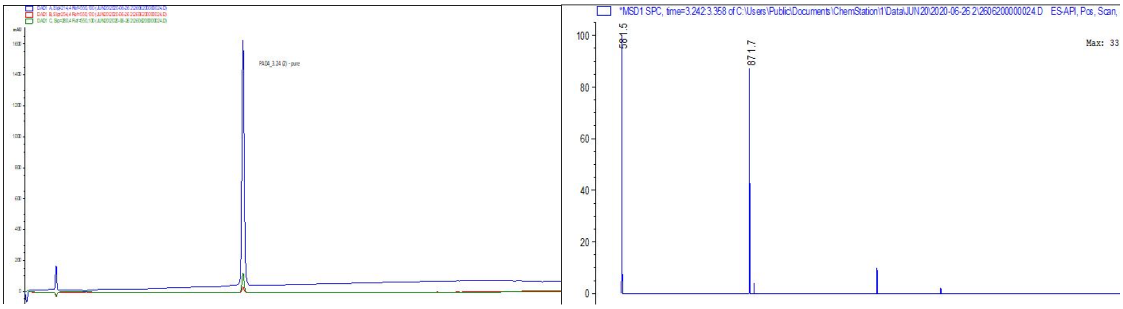

Supplementary Figure 38. PADI4_3_K10V Left: HPLC trace, showing A214 in blue, A254 in red and A280 in green. Right: ESI-MS trace

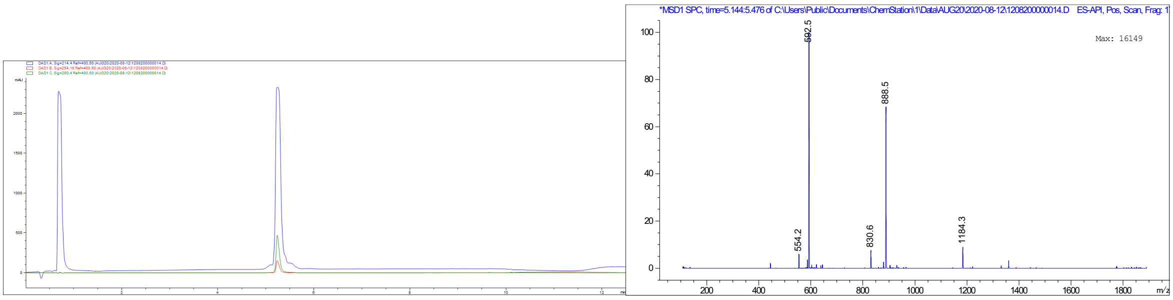

Supplementary Figure 39. PADI4_3_H4R_K10I Left: HPLC trace, showing A214 in blue, A254 in red and A280 in green. Right: ESI-MS trace

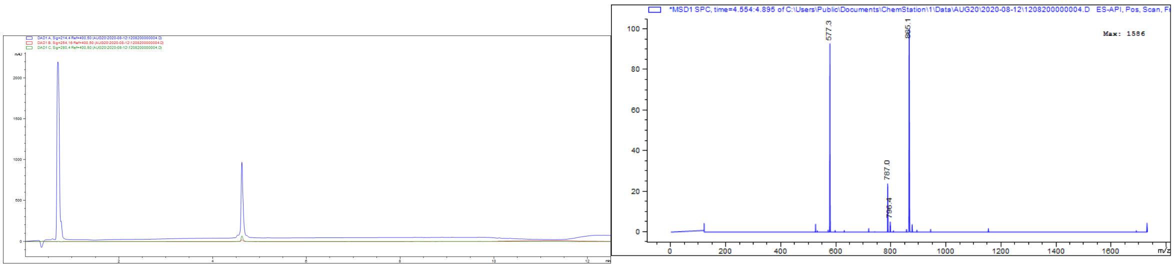

Supplementary Figure 40. PADI4_3_R7N Left: HPLC trace, showing A214 in blue, A254 in red and A280 in green. Right: ESI-MS trace

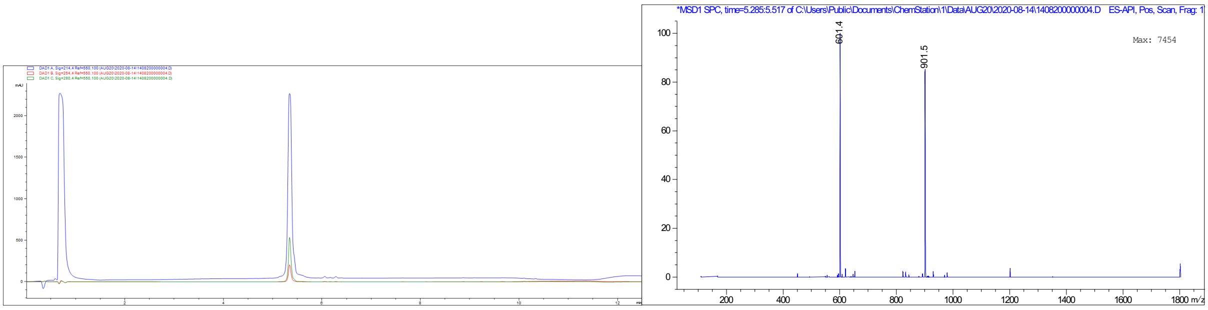

Supplementary Figure 41. PADI4_3_R7W Left: HPLC trace, showing A214 in blue, A254 in red and A280 in green. Right: ESI-MS trace

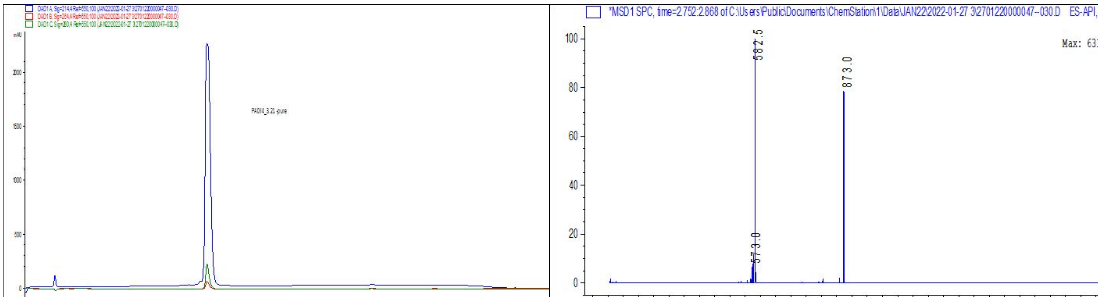

Supplementary Figure 42. PADI4_3_Y6H Left: HPLC trace, showing A214 in blue, A254 in red and A280 in green. Right: ESI-MS trace

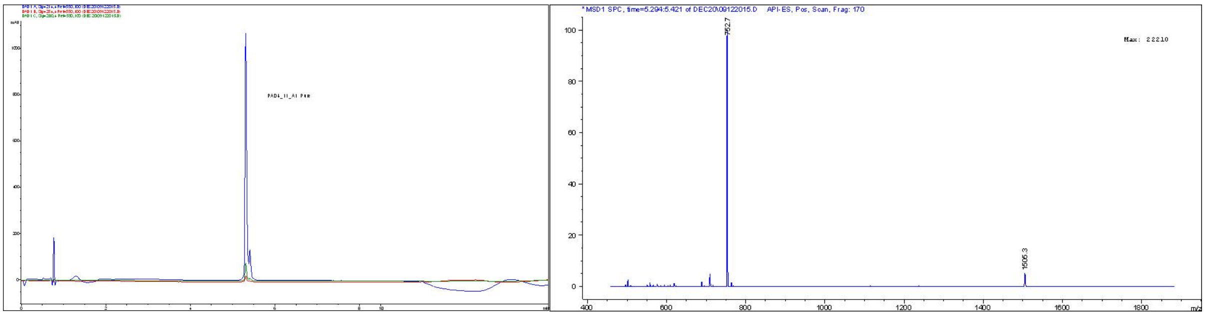

Supplementary Figure 43. PADI4_11_Y1A Left: HPLC trace, showing A214 in blue, A254 in red and A280 in green. Right: ESI-MS trace

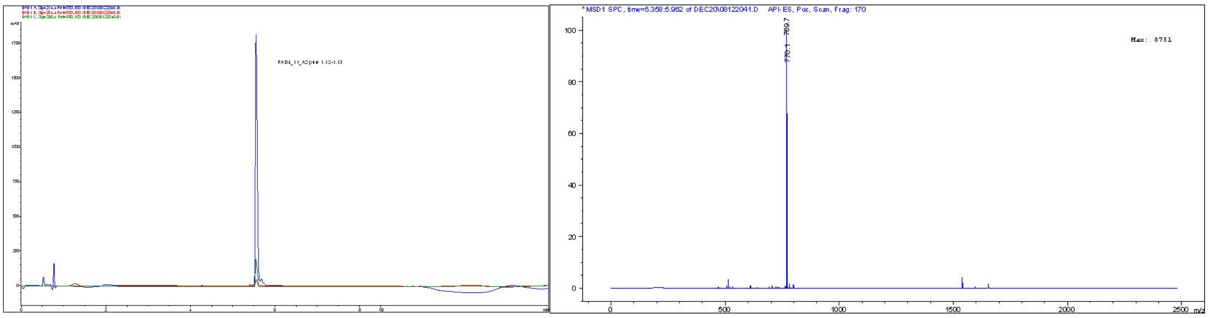

Supplementary Figure 44. PADI4_11_E2A Left: HPLC trace, showing A214 in blue, A254 in red and A280 in green. Right: ESI-MS trace

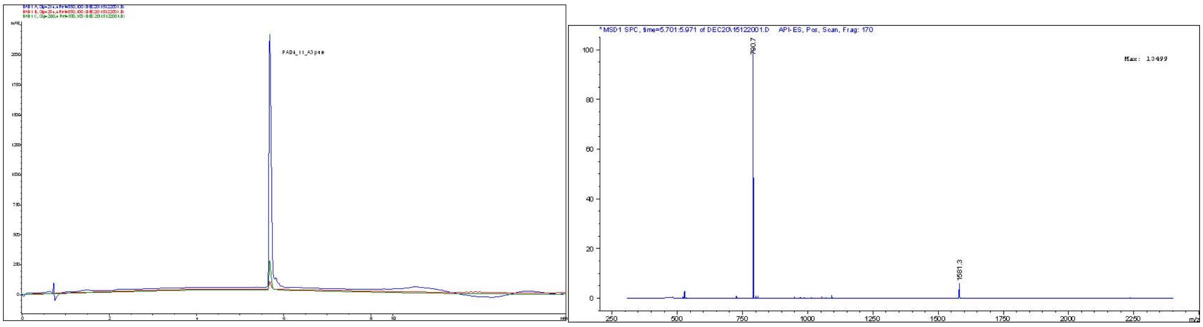

Supplementary Figure 45. PADI4_11_S3A Left: HPLC trace, showing A214 in blue, A254 in red and A280 in green. Right: ESI-MS trace

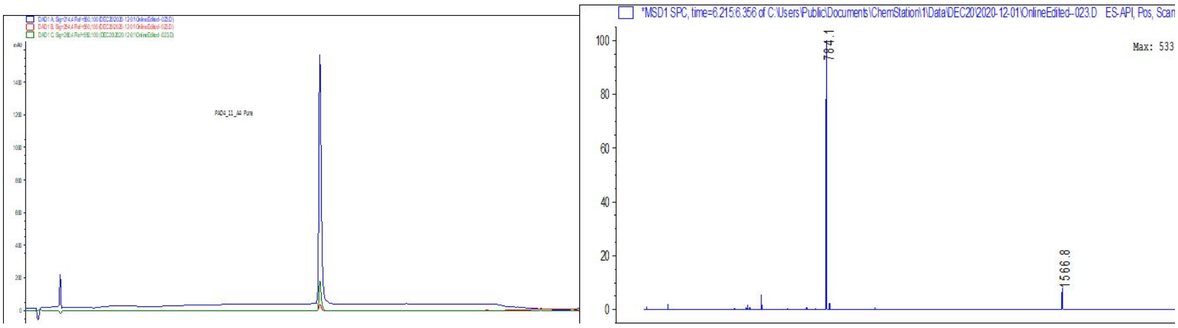

Supplementary Figure 46. PADI4_11_C4A Left: HPLC trace, showing A214 in blue, A254 in red and A280 in green. Right: ESI-MS trace

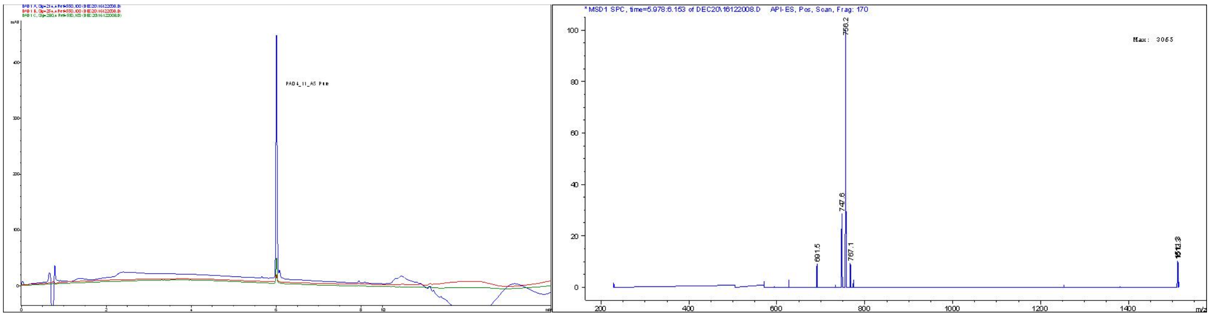

Supplementary Figure 47. PADI4_11_R5A Left: HPLC trace, showing A214 in blue, A254 in red and A280 in green. Right: ESI-MS trace

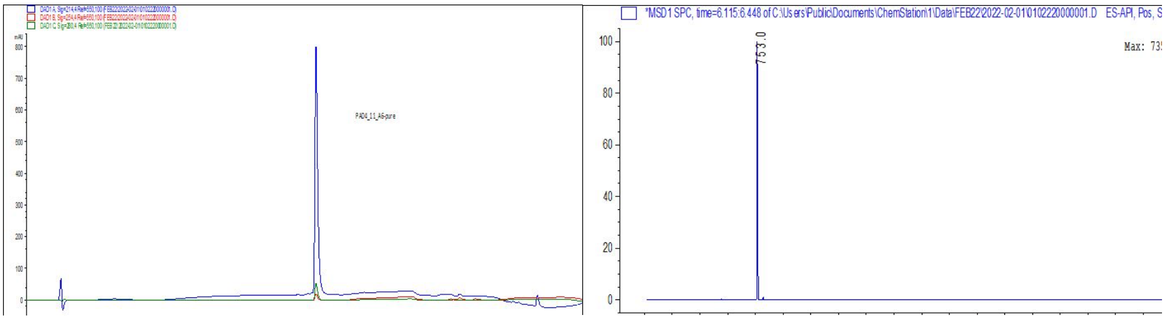

Supplementary Figure 48. PADI4_11_Y6A Left: HPLC trace, showing A214 in blue, A254 in red and A280 in green. Right: ESI-MS trace

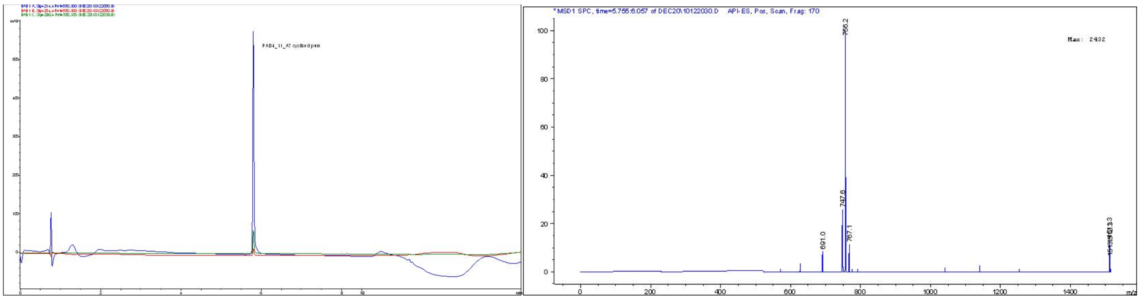

Supplementary Figure 49. PADI4_11_R7A Left: HPLC trace, showing A214 in blue, A254 in red and A280 in green. Right: ESI-MS trace

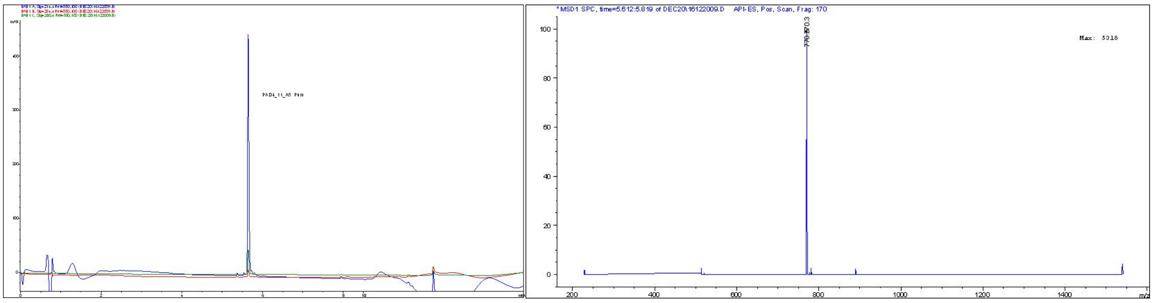

Supplementary Figure 50. PADI4_11_Q8A Left: HPLC trace, showing A214 in blue, A254 in red and A280 in green. Right: ESI-MS trace

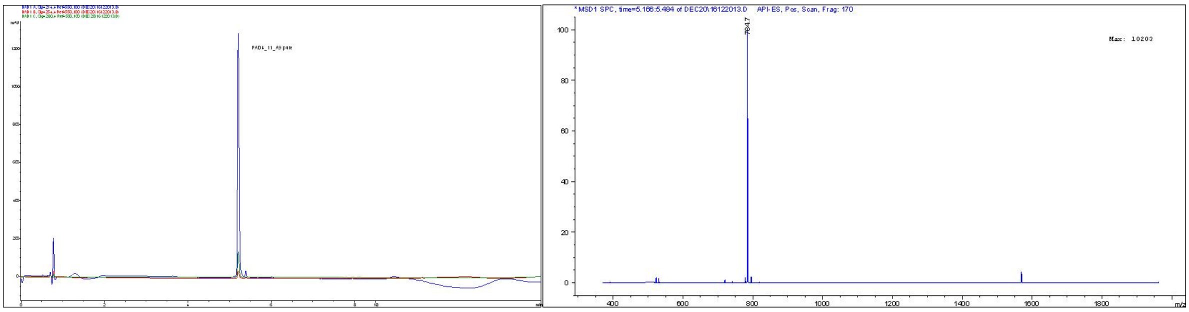

Supplementary Figure 51. PADI4_11_V9A Left: HPLC trace, showing A214 in blue, A254 in red and A280 in green. Right: ESI-MS trace

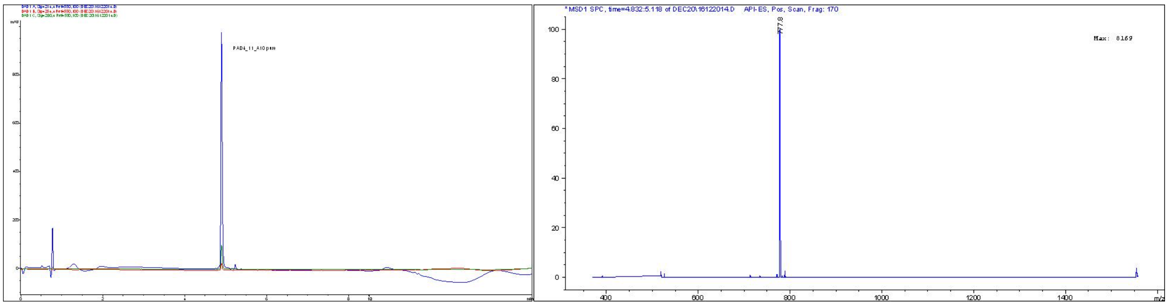

Supplementary Figure 52. PADI4_11_L10A Left: HPLC trace, showing A214 in blue, A254 in red and A280 in green. Right: ESI-MS trace

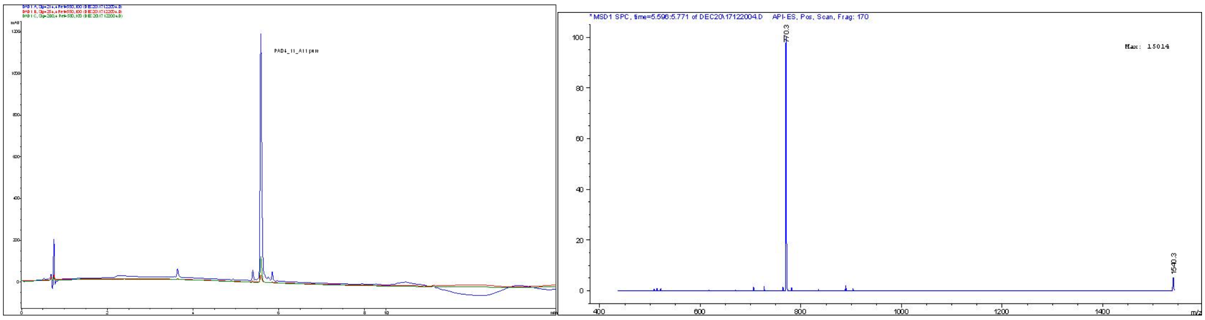

Supplementary Figure 53. PADI4_11_Q11A Left: HPLC trace, showing A214 in blue, A254 in red and A280 in green. Right: ESI-MS trace

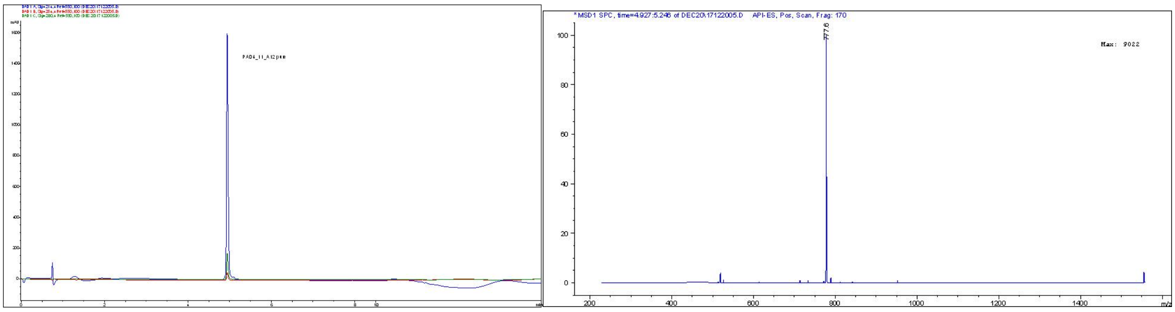

Supplementary Figure 54. PADI4_11_L12A Left: HPLC trace, showing A214 in blue, A254 in red and A280 in green. Right: ESI-MS trace

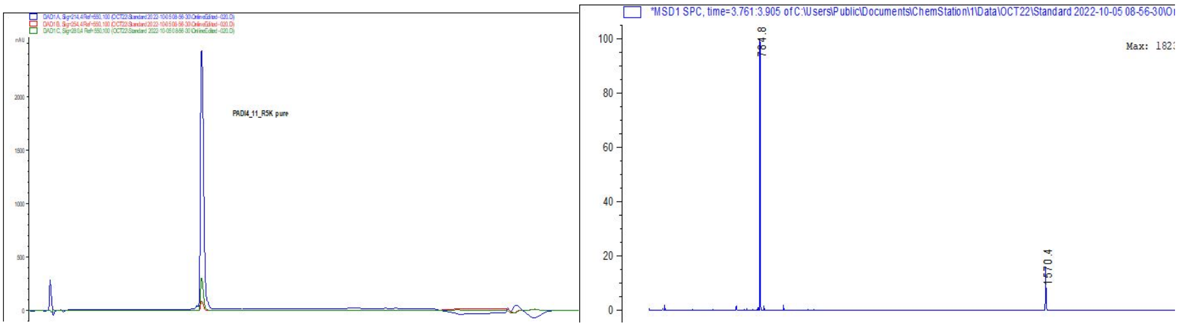

Supplementary Figure 55. PADI4_11_R5K Left: HPLC trace, showing A214 in blue, A254 in red and A280 in green. Right: ESI-MS trace

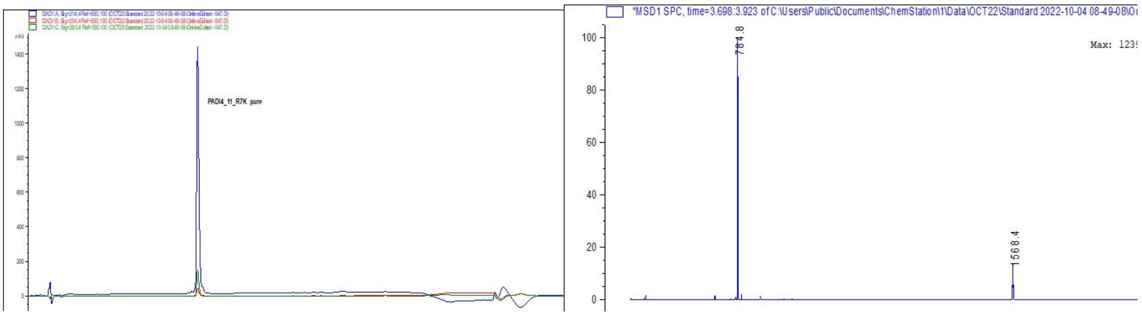

Supplementary Figure 56. PADI4_11_R7K Left: HPLC trace, showing A214 in blue, A254 in red and A280 in green.

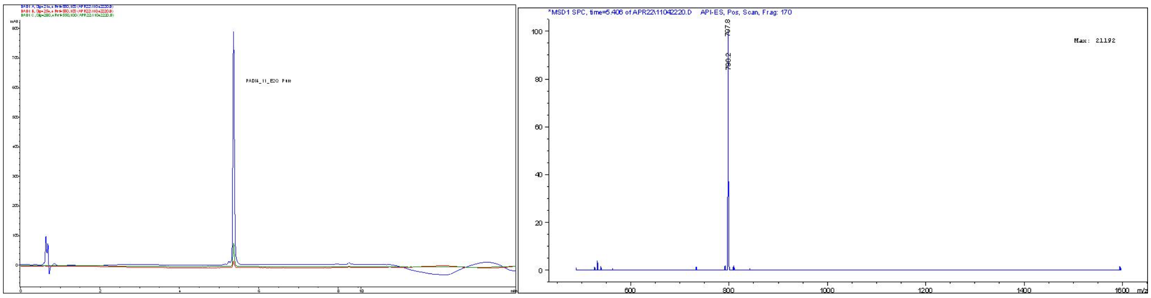

Supplementary Figure 57. PADI4_11_E2Q Left: HPLC trace, showing A214 in blue, A254 in red and A280 in green. Right: ESI-MS trace

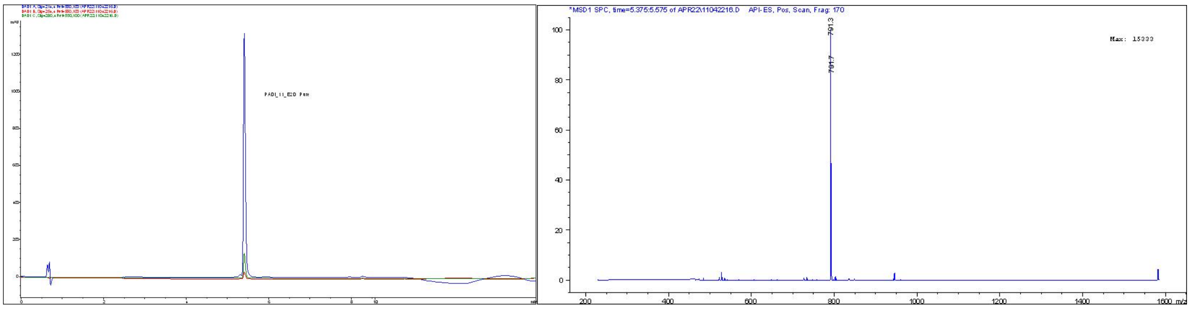

Supplementary Figure 58. PADI4_11_E2D Left: HPLC trace, showing A214 in blue, A254 in red and A280 in green. Right: ESI-MS trace

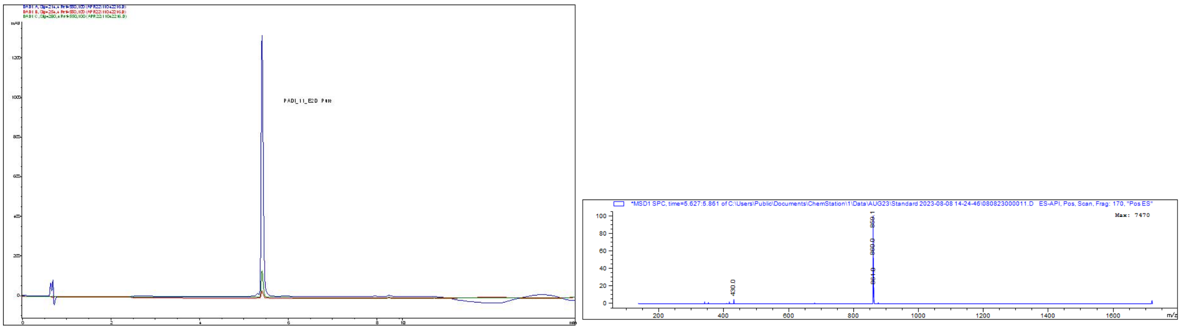

Supplementary Figure 59. PADI4_11B Left: HPLC trace, showing A214 in blue, A254 in red and A280 in green. Right: ESI-MS trace

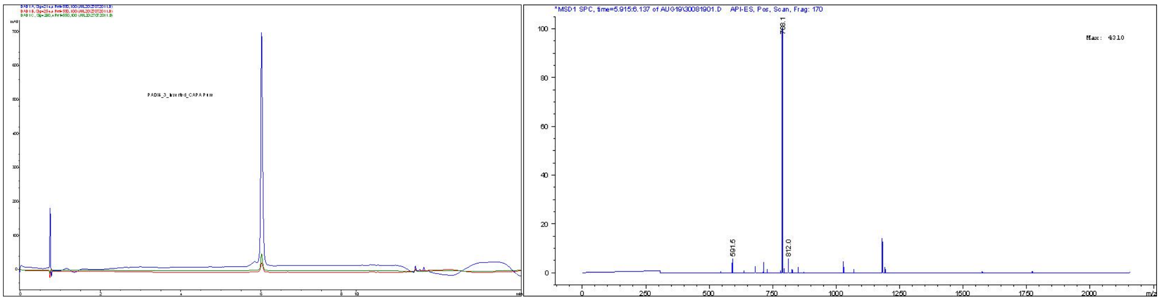

Supplementary Figure 60. PADI4_3_CAPA Left: HPLC trace, showing A214 in blue, A254 in red and A280 in green. Right: ESI-MS trace

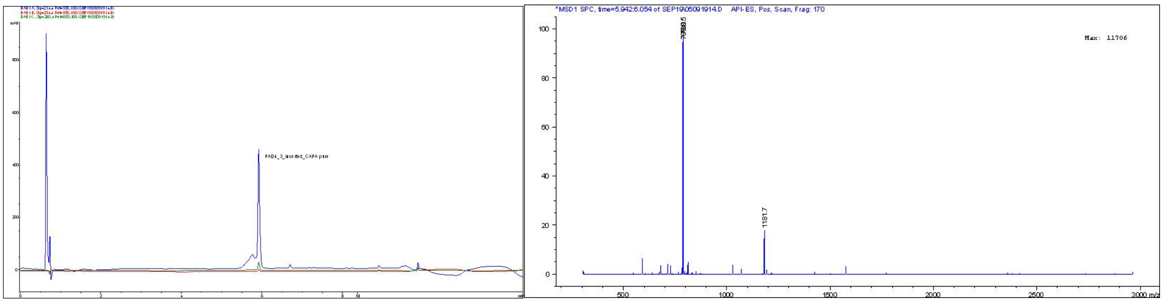

Supplementary Figure 61. PADI4_3i_CAPA Left: HPLC trace, showing A214 in blue, A254 in red and A280 in green. Right: ESI-MS trace

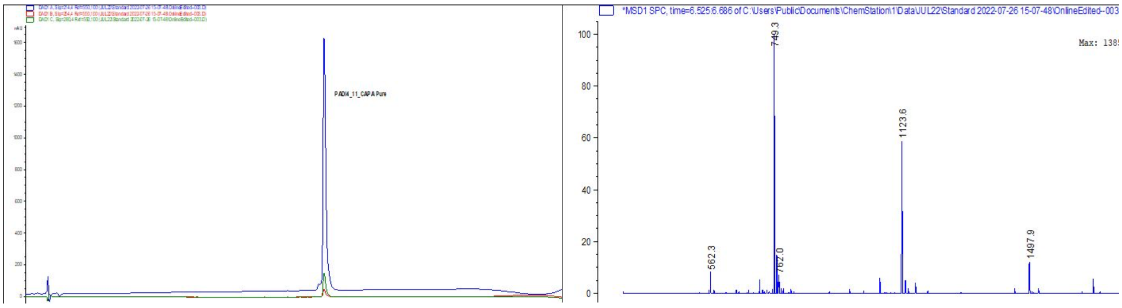

Supplementary Figure 62. PADI4_11_CAPA Left: HPLC trace, showing A214 in blue, A254 in red and A280 in green. Right: ESI-MS trace

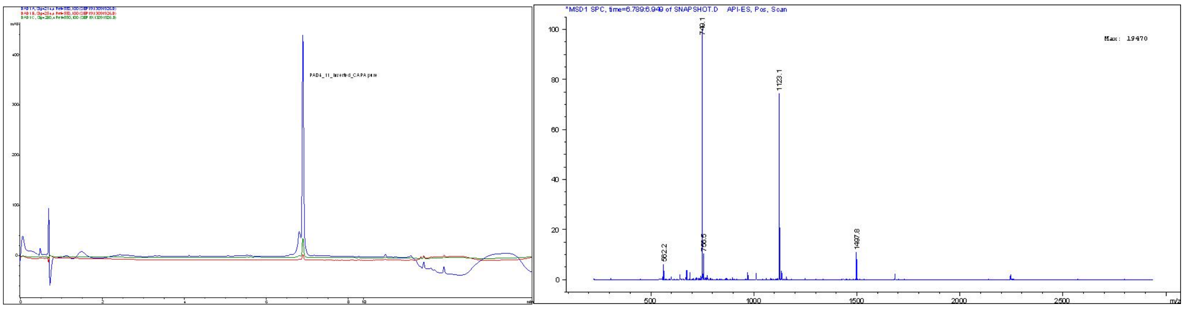

Supplementary Figure 63. PADI4_11i_CAPA Left: HPLC trace, showing A214 in blue, A254 in red and A280 in green. Right: ESI-MS trace

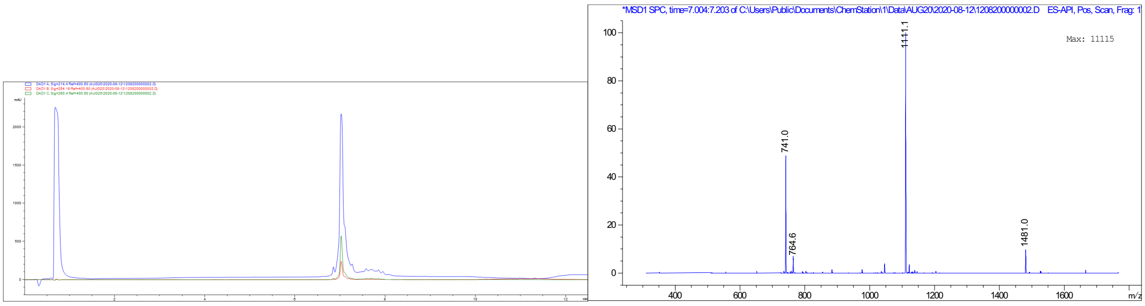

Supplementary Figure 64. PADI4_7_bio Left: HPLC trace, showing A214 in blue, A254 in red and A280 in green. Right: ESI-MS trace

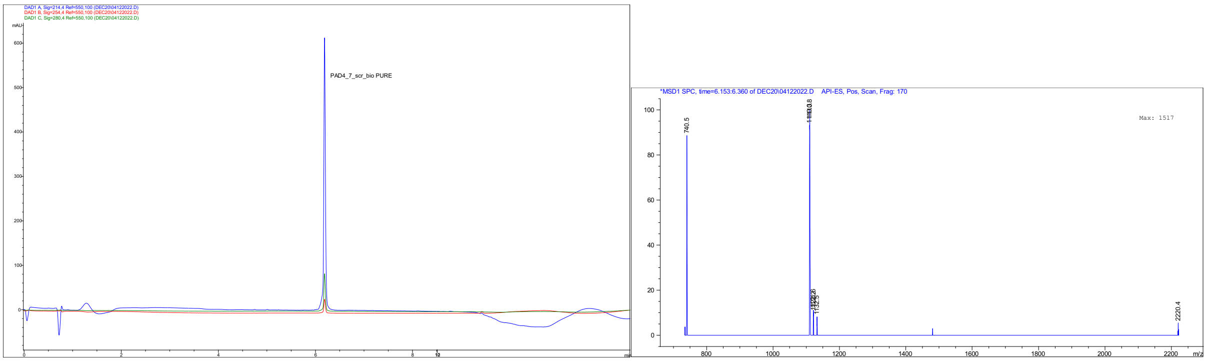

Supplementary Figure 65. PADI4_7scr_bio Left: HPLC trace, showing A214 in blue, A254 in red and A280 in green. Right: ESI-MS trace

Supplementary Table 1. SPR data of selected macrocyclic peptides enriched in the RaPID selections. Data shows average ± standard deviation of three independent experiments.

	10 mM Ca^{2+}			0 mM Ca^{2+}			200 µM Cl-amidine + 10 mM Ca^{2+}		
	**Kd (nM)**	**Kon (1/Ms)**	**Koff (1/s)**	**Kd (nM)**	**Kon (1/Ms)**	**Koff (1/s)**	**Kd (nM)**	**Kon (1/Ms)**	**Koff (1/s)**
**PAD4_1**	205 ± 52	$7.6\cdot10^4 \pm 0.8\cdot.10^4$	$2\cdot10^{-2} \pm 3\cdot10^{-3}$	105 ± 17	$9.5\cdot10^4 \pm 0.6\cdot10^4$	$1\cdot10^{-2} \pm 2\cdot10^{-3}$	728 ± 175	$4\cdot10^4 \pm 3\cdot10^3$	$3\cdot10^{-2} \pm 5\cdot10^{-3}$
**PAD4_2**	13 ± 4	$8\cdot10^4 \pm 2\cdot10^4$	$1.0\cdot10^{-3} \pm 0.5\cdot10^{-3}$	>5 µM	-	-	>5 µM	-	-
**PAD4_3**	2.7 ± 0.5	$2.5\cdot10^5 \pm 0.2\cdot10^5$	$7\cdot10^{-4} \pm 1\cdot10^{-4}$	>5 µM	-	-	>5 µM	-	-
**PAD4_7**	39 ± 24	$5\cdot10^6 \pm 5\cdot10^6$	$2\cdot10^{-1} \pm 2\cdot10^{-1}$	9 ± 1	$1\cdot10^7 \pm 1\cdot10^7$	$1\cdot10^{-1} \pm 1\cdot10^{-1}$	36 ± 12	$9\cdot10^6 \pm 6\cdot10^6$	$4\cdot10^{-1} \pm 2\cdot10^{-1}$
**PAD4_11**	457 ± 109	$2\cdot10^5 \pm 7\cdot10^5$	$1\cdot10^{-1} \pm 2\cdot10^{-2}$	>5 µM	-	-	382 ± 27	$3\cdot10^5 \pm 5\cdot10^4$	$1\cdot10^{-1} \pm 1\cdot10^{-2}$
**PAD4_12**	679 ± 83	$2\cdot10^5 \pm 5 \cdot10^4$	$1\cdot10^{-1} \pm 3\cdot10^{-2}$	>5 µM	-	-	674 ± 191	$3\cdot10^5 \pm 8\cdot10^4$	$2\cdot10^{-1} \pm 2\cdot10^{-2}$

Supplementary Table 2. Cryo-EM data collection, refinement, and validation statistics.

	PADI4_3 (EMDB-19011) (PDB 8R8U)	PADI4_11 (EMDB-19012) (PDB 8R8V)
**Data collection and processing**		
Voltage (kV)	300	300
Electron exposure (e–/Å^2)	28.0	28.0
Defocus range (μm)	-1 – -3	-1 – -3
Pixel size (Å)	0.95	0.95
Symmetry imposed	C2	C2
Final particle images (no.)	127k	79k
Map resolution (Å)	3.1	3.6
FSC threshold 0.143		
**Refinement**		
Initial model used (PDB code)	1WD9	1WD9
Model resolution (Å)	3.2	3.7
FSC threshold 0.5		
Map sharpening *B* factor (Å^2)	-146.3	-187.6
Model composition		
Non-hydrogen atoms	10140	10198
Protein residues	1282	1292
Ligands	10	10
B factors (Å^2)		
Protein	46.7	63.1
Ligand	29.6	49.2
R.m.s. deviations		
Bond lengths (Å)	0.002	0.005
Bond angles (°)	0.456	0.708
Validation		
MolProbity score	1.20	1.64
Clashscore	4.12	7.29
Poor rotamers (%)	0.18	0.26
Ramachandran plot		
Favored (%)	98.01	96.38
Allowed (%)	1.99	3.62
Disallowed (%)	0.00	0.00

Supplementary Table 3. SPR binding affinities for the different PADI4_3 analogues. Data represents average ± standard deviation of three independent replicates.

	**Sequence**	**K_D (nM)**	**Ka (1/Ms)**	**Kd (1/s)**
**PADI4_3_Y1Y(Ome)**	dY(OMe)RDHHYRHPKYCG	23 ± 2	$1 \cdot 10^{05} \pm 1 \cdot 10^{04}$	$3 \cdot 10^{-03} \pm 2 \cdot 10^{-05}$
**PAD4_3_R2A**	dYADHHYRHPKYCG	239 ± 8	$2 \cdot 10^{05} \pm 5 \cdot 10^{04}$	$3 \cdot 10^{-02} \pm 2 \cdot 10\text{-}^{02}$
**PAD4_3_R2cit**	dYcitDHHYRHPKYCG	20 ± 11	$5 \cdot 10^{05} \pm 6 \cdot 10^{05}$	$2 \cdot 10^{-03} \pm 2 \cdot 10^{-04}$
**PADI4_3_R2K**	dYKDHHYRHPKYCG	9 ± 2	$2 \cdot 10^{05} \pm 2 \cdot 10^{04}$	$2 \cdot 10^{-03} \pm 4 \cdot 10^{-04}$
**PADI4_3_H4R**	dYRDRHYRHPKYCG	14 ± 6	$1 \cdot 10^{06} \pm 1 \cdot 10^{06}$	$1 \cdot 10^{-02} \pm 7 \cdot 10^{-03}$
**PADI4_3_Y6H**	dYRDHHHRHPKYCG	30 ± 15	$6 \cdot 10^{04} \pm 3 \cdot 10^{04}$	$1 \cdot 10^{-03} \pm 2 \cdot 10\text{-}04$
**PADI4_3_R7N**	dYRDHHYNHPKYCG	41 ± 18	$1 \cdot 10^{04} \pm 6 \cdot 10^{03}$	$4 \cdot 10^{-04} \pm 1 \cdot 10^{-05}$
**PADI4_3_R7W**	dYRDHHYWHPKYCG	6 ± 3	$2 \cdot 10^{05} \pm 1 \cdot 10^{05}$	$2 \cdot 10^{-03} \pm 2 \cdot 10^{-03}$
**PAD4_3_R7A**	dYRDHHYAHPKYCG	7 ± 1	$7 \cdot 10^{04} \pm 2 \cdot 10^{04}$	$5 \cdot 10^{-04} \pm 7 \cdot 10^{-05}$
**PADI4_3_K10I**	dYRDHHYRHPIYCG	4 ± 2	$1 \cdot 10^{05} \pm 4 \cdot 10^{04}$	$4 \cdot 10^{-04} \pm 2 \cdot 10^{-04}$
**PADI4_3_K10V**	dYRDHHYRHPVYCG	5 ± 2	$8 \cdot 10^{04} \pm 2 \cdot 10^{04}$	$4 \cdot 10^{-04} \pm 4 \cdot 10^{-05}$
**PADI4_3H4R_K10_I**	dYRDRHYRHPIYCG	7 ± 3	$7 \cdot 10^{05} \pm 4 \cdot 10^{05}$	$2 \cdot 10^{-03} \pm 2 \cdot 10^{-03}$

Supplementary Table 4. Primers used in this study. SP18 is an 18-atom hexa-ethyleneglycol spacer.

Primer Name	Sequence
*mPadi4*_fw	CATATGATGGCCCAGGGTGCGGTGATC
*mPadi4*_rev	CTCGAGTCAGGGCACCATGTGCCACCAC
hPADI4_D165A_fw	ATCTTCTGCCATGGCCTGCGAGGATGATGAAG
hPADI4_D165A_rev	CATCATCCTCGCAGGCCATGGCAGAAGATTCG
hPADI4_D168A_fw	CTGCCATGGACTGCGAGGCTGATGAAGTGCTTGAC
hPADI4_D168A_rev	GTCAAGCACTTCATCAGCCTCGCAGTCCATGGCAG
hPADI4_D167A_fw	CTTCTGCCATGGACTGCGCGGATGATGAAGTGCTTGAC
hPADI4_D167A_rev	GTCAAGCACTTCATCATCCGCGCAGTCCATGGCAGAAG
hPADI4_N373A_fw	TTCGACTCTCCAAGGGCCAGAGGCCTGAAGG
hPADI4_N373A_rev	CTCCTTCAGGCCTCTGGCCCTTGGAGAGTCG
hPADI4_R394A_fw	GATTTTGGCTATGTAACTGCAGGGCCCCAAACAGGGGG
hPADI4_R394A_rev	CCCCCTGTTTGGGGCCCTGCAGTTACATAGCCAAAATC
hPADI4_C166F_fw	CTCGAATCTTCTGCCATGGACTCCGAGGATGATGAAGTGCTT G
hPADI4_C166F_rev	CAAGCACTTCATCATCCTCGAAGTCCATGGCAGAAGATTCGA G
T7g10M.F48	TAATACGACTCACTATAGGGTTAACTTTAAGAAGGAGATATAC ATATG
long_HA_reverse	TTTCCGCCCCCCGTCCTAAGAACCAGAACCAGAACCTGCATA GTCGGGCACGTCGTATGGGTAGCTGCCGCTGCC
short_HA_reverse	TTTCCGCCCCCCGTCCTAAGAACCAGAACCAGAACC
PAD4_3_F_NNK1	CTATAGGGTTAACTTTAAGAAGGAGATATACATATGNNKGATC ATCATTATAGGCATCCGAAGTATTGC
PAD4_3_F_NNK2	CTATAGGGTTAACTTTAAGAAGGAGATATACATATGAGGNNK CATCATTATAGGCATCCGAAGTATTGC
PAD4_3_F_NNK3	CTATAGGGTTAACTTTAAGAAGGAGATATACATATGAGGGAT NNKCATTATAGGCATCCGAAGTATTGC
PAD4_3_F_NNK4	CTATAGGGTTAACTTTAAGAAGGAGATATACATATGAGGGAT CATNNKTATAGGCATCCGAAGTATTGC
PAD4_3_F_NNK5	CTATAGGGTTAACTTTAAGAAGGAGATATACATATGAGGGAT CATCATNNKAGGCATCCGAAGTATTGC
PAD4_3_R_NNK6	TATGGGTAGCTGCCGCTGCCGCAATACTTCGGATGMNNATA ATGATGATCCCTCATAT
PAD4_3_R_NNK7	TATGGGTAGCTGCCGCTGCCGCAATACTTCGGMNNCCTATA ATGATGATCCCTCATAT

PAD4_3_R_NNK8	TATGGGTAGCTGCCGCTGCCGCAATACTTMNNATGCCTATAATGATGATCCCTCATAT
PAD4_3_R_NNK9	TATGGGTAGCTGCCGCTGCCGCAATAMNNCGGATGCCTATAATGATGATCCCTCATAT
PAD4_3_R_NNK10	TATGGGTAGCTGCCGCTGCCGCAMNNCTTCGGATGCCTATAATGATGATCCCTCATAT
PADI4_AttB1_F	GGGGACAAGTTTGTA CAAAAAAGCAGGCTTCACCATGGCCCAGGGGACATTGATCCG
PADI4_AttB2_R	GGGGACCACTTTGTACAAGAAAGCTGGGTCTCAGGGCACCATGTTCCACC
Pu_linker	[5'Phos]CTCCCGCCCCCCGTCC[SP18][SP18][SP18][SP18][SP18]CC[Puromycin]

Supplementary Table 5. Macrocyclic peptides used in this study. All peptides are cyclic (thioether bond between the Acetylated N-terminus and the cysteine side chain) unless otherwise specified. Abbreviations: Ac – N-terminal acetylation; y – D-Tyrosine; Cit – citrulline; Y(OMe) – O-Methyl-Tyrosine; B – β-Ala; K(Z) – Z attached via an amide bond to the lysine side chain where Z could be: bio (biotin), Cl (cl-alkane). Residues modified from the parent sequences are highlighted in bold. *Mass determined by MALDI.

Peptide Name	Peptide Sequence	Mass Calculated /Da	Deconvoluted Mass Observed /Da
PAD4_1	yFYRIGFWYPNYQC(S-)G-NH_2	2016.3	2015.3*
PAD4_2	yRDHRSPFDGYC(S-)G-NH_2	1611.7	1611.5
PAD4_3	yRDHHYRHPKYC(S-)G-NH_2	1771.0	1770.1
PAD4_3i	y**DR**HHYRHPKYC(S-)G-NH_2	1771.0	1769.8
PAD4_4	yHRLIVVIYVC(S-)G-NH_2	1473.8	1473.6
PAD4_5	yATPWLIVVLC(S-)G-NH_2	1373.7	1372.8
PAD4_6	yLVLTIRLVLC(S-)G-NH_2	1401.8	1400.9
PAD4_7	yYPKGSWGYKLFC(S-)G-NH_2	1708.0	1707.5
PAD4_8	yTLWTVLVVIC(S-)G-NH_2	1406.7	1427.5* (+Na)
PAD4_9	yAQWYIWVLLC(S-)G-NH_2	1553.9	1553.4
PAD4_10	yPWEISVWLLYC(S-)G-NH_2	1668.0	1666.3
PAD4_11	YESC(S-)RYRQVLQL-NH_2	1596.8	1596.0
PAD4_11_i	Y**SE**C(S-)RYRQVLQL-NH_2	1596.8	1597.6
PAD4_12	YEPC(S-)RFREILDL-NH_2	1592.8	1593.5
PAD4_3_R2A	y**A**DHHYRHPKYC(S-)G-NH_2	1685.8	1685.8
PAD4_3_R7A	yRDHHY**A**HPKYC(S-)G-NH_2	1685.8	1684.1
PAD4_3_R2cit	y**Cit**DHHYRHPKYC(S-)G-NH_2	1771.9	1772.0
PAD4_3_R2K	y**K**DHHYRHPKYC(S-)G-NH_2	1742.3	1742.8
PAD4_3_y1(OMe)Y	Y(OMe)RDHHYRHPKYC(S-)G-NH_2	1785.0	1784.9
P4_3_H4R	yRD**R**HYRHPKYC(S-)G-NH_2	1790.0	1789.4
P4_3_K10I	yRDHHYRHP**I**YC(S-)G-NH_2	1755.9	1755.6
P4_3_K10V	yRDHHYRHP**V**YC(S-)G-NH_2	1741.9	1741.6
P4_3_H4R_K10I	yRD**R**HYRHP**I**YC(S-)G-NH_2	1775.0	1774.9
P4_3_R7N	yRDHHY**N**HPKYC(S-)G-NH_2	1728.9	1728.6
P4_3_R7W	yRDHHY**W**HPKYC(S-)G-NH_2	1801.0	1800.9
P4_3_Y6H	yRDHH**H**RHPKYC(S-)G-NH_2	1744.9	1744.7
PAD4_11_Y1A	**A**ESC(S-)RYRQVLQL-NH_2	1504.7	1503.8
PAD4_11_E2A	Y**A**SC(S-)RYRQVLQL-NH_2	1538.8	1539.4
PAD4_11_S3A	YE**A**C(S-)RYRQVLQL-NH_2	1580.8	1581.3
PAD4_11_C4A	Ac-YES**A**RYRQVLQL-NH_2	1524.7	1565.5
PAD4_11_R5A	YESC(S-)**A**YRQVLQL-NH_2	1511.7	1510.7
PAD4_11_Y6A	YESC(S-)R**A**RQVLQL-NH_2	1504.7	1505.8
PAD4_11_R7A	YESC(S-)RY**A**QVLQL-NH_2	1511.7	1512.2
PAD4_11_Q8A	YESC(S-)RYR**A**VLQL-NH_2	1539.8	1538.8
PAD4_11_V9A	YESC(S-)RYRQ**A**LQL-NH_2	1568.8	1569.4
PAD4_11_L10A	YESC(S-)RYRQV**A**QL-NH_2	1554.8	1555.6
PAD4_11_Q11A	YESC(S-)RYRQVL**A**L-NH_2	1539.8	1540.3
PAD4_11_L12A	YESC(S-)RYRQVLQ**A**-NH_2	1554.8	1555.2
PADI4_11_R5K	YESC(S-)**K**YRQVLQL-NH_2	1568.3	1568.0
PADI4_11_R7K	YESC(S-)RY**K**QVLQL-NH_2	1568.3	1568.4

PADI4_11_E2Q	YQSC(S-)RYRQVLQL-NH_2	1593.8	see 2+ ion 797.8
PADI4_11_E2D	YDSC(S-)RYRQVLQL-NH_2	1580.8	1581.0
PADI4_11B	YESC(S-)Y-NH_2	858.9	858.1
PAD4_3_CAPA	yRDHHYRHPKYC(S-)GSBK(Cl)-NH_2	2364.4	2362.4
PAD4_3i_CAPA	y**DR**HHYRHPKYC(S-)GSBK(Cl)-NH_2	2364.4	2364.3
PAD4_11_CAPA	YESC(S-)RYRQVLQLGSBK(Cl)-NH_2	2247.5	2245.5
PAD4_11i_CAPA	Y**SE**C(S-)RYRQVLQLGSBK(Cl)-NH_2	2246.9	2244.8
P4_7_bio	yYPKGSWGYKLFC(S-)GSBK(Bio)-NH_2	2220.6	2220.1
P4_7scr_bio	yYPKGSWGYKLFC(S-)GSBK(Bio)-NH_2	2220.6	2220.4

hPADI1 plasmid

```
ACAATCGATAGATTGTCGCACCTGATTGCCCGACATTATCGCGAGCCCATTTATACCCATATAAATCAGCATCCATGTTGGAATTTAATCGCGGCCTAGAGCAAGAC
GTTTCCCGTTGAATATGGCTCATAACACCCCTTGTATTACTGTTTATGTAAGCAGACAGTTTTATTGTTCATGACCAAAATCCCTTAACGTGAGTTTTCGTTCCACT
GAGCGTCAGACCCCGTAGAAAAGATCAAAGGATCTTCTTGAGATCCTTTTTTTCTGCGCGTAATCTGCTGCTTGCAAACAAAAAAACCACCGCTACCAGCGGTGGTT
TGTTTGCCGGATCAAGAGCTACCAACTCTTTTTCCGAAGGTAACTGGCTTCAGCAGAGCGCAGATACCAAATACTGTCCTTCTAGTGTAGCCGTAGTTAGGCCACCA
CTTCAAGAACTCTGTAGCACCGCCTACATACCTCGCTCTGCTAATCCTGTTACCAGTGGCTGCTGCCAGTGGCGATAAGTCGTGTCTTACCGGGTTGGACTCAAGAC
GATAGTTACCGGATAAGGCGCAGCGGTCGGGCTGAACGGGGGGTTCGTGCACACAGCCCAGCTTGGAGCGAACGACCTACACCGAACTGAGATACCTACAGCGTGAG
CTATGAGAAAGCGCCACGCTTCCCGAAGGGAGAAAGGCGGACAGGTATCCGGTAAGCGGCAGGGTCGGAACAGGAGAGCGCACGAGGGAGCTTCCAGGGGGAAACGC
CTGGTATCTTTATAGTCCTGTCGGGTTTCGCCACCTCTGACTTGAGCGTCGATTTTTGTGATGCTCGTCAGGGGGGCGGAGCCTATGGAAAAACGCCAGCAACGCGG
CCTTTTTACGGTTCCTGGCCTTTTGCTGGCCTTTTGCTCACATGTTCTTTCCTGCGTTATCCCCTGATTCTGTGGATAACCGTATTACCGCCTTTGAGTGAGCTGAT
ACCGCTCGCCGCAGCCGAACGACCGAGCGCAGCGAGTCAGTGAGCGAGGAAGCGGAAGAGCGCCTGATGCGGTATTTTCTCCTTACGCATCTGTGCGGTATTTCACA
CCGCAATGGTGCACTCTCAGTACAATCTGCTCTGATGCCGCATAGTTAAGCCAGTATACACTCCGCTATCGCTACGTGACTGGGTCATGGCTGCGCCCCGACACCCG
CCAACACCCGCTGACGCGCCCTGACGGGCTTGTCTGCTCCCGGCATCCGCTTACAGACAAGCTGTGACCGTCTCCGGGAGCTGCATGTGTCAGAGGTTTTCACCGTC
ATCACCGAAACGCGCGAGGCAGCTGCGGTAAAGCTCATCAGCGTGGTCGTGAAGCGATTCACAGATGTCTGCCTGTTCATCCGCGTCCAGCTCGTTGAGTTTCTCCA
GAAGCGTTAATGTCTGGCTTCTGATAAAGCGGGCCATGTTAAGGGCGGTTTTTTCCTGTTTGGTCACTGATGCCTCCGTGTAAGGGGGATTTCTGTTCATGGGGGTA
ATGATACCGATGAAACGAGAGAGGATGCTCACGATACGGGTTACTGATGATGAACATGCCCGGTTACTGGAACGTTGTGAGGGTAAACAACTGGCGGTATGGATGCG
GCGGGACCAGAGAAAAATCACTCAGGGTCAATGCCAGCGCTTCGTTAATACAGATGTAGGTGTTCCACAGGGTAGCCAGCAGCATCCTGCGATGCAGATCCGGAACA
TAATGGTGCAGGGCGCTGACTTCCGCGTTTCCAGACTTTACGAAACACGGAAACCGAAGACCATTCATGTTGTTGCTCAGGTCGCAGACGTTTTGCAGCAGCAGTCG
CTTCACGTTCGCTCGCGTATCGGTGATTCATTCTGCTAACCAGTAAGGCAACCCCGCCAGCCTAGCCGGGTCCTCAACGACAGGAGCACGATCATGCGCACCCGTGG
GGCCGCCATGCCGGCGATAATGGCCTGCTTCTCGCCGAAACGTTTGGTGGCGGGACCAGTGACGAAGGCTTGAGCGAGGGCGTGCAAGATTCCGAATACCGCAAGCG
ACAGGCCGATCATCGTCGCGCTCCAGCGAAAGCGGTCCTCGCCGAAAATGACCCAGAGCGCTGCCGGCACCTGTCCTACGAGTTGCATGATAAAGAAGACAGTCATA
AGTGCGGCGACGATAGTCATGCCCCGCGCCCACCGGAAGGAGCTGACTGGGTTGAAGGCTCTCAAGGGCATCGGTCGAGATCCCGGTGCCTAATGAGTGAGCTAACT
TACATTAATTGCGTTGCGCTCACTGCCCGCTTTCCAGTCGGGAAACCTGTCGTGCCAGCTGCATTAATGAATCGGCCAACGCGCGGGGAGAGGCGGTTTGCGTATTG
GGCGCCAGGGTGGTTTTTCTTTTCACCAGTGAGACGGGCAACAGCTGATTGCCCTTCACCGCCTGGCCCTGAGAGAGTTGCAGCAAGCGGTCCACGCTGGTTTGCCC
CAGCAGGCGAAAATCCTGTTTGATGGTGGTTAACGGCGGGATATAACATGAGCTGTCTTCGGTATCGTCGTATCCCACTACCGAGATATCCGCACCAACGCGCAGCC
CGGACTCGGTAATGGCGCGCATTGCGCCCAGCGCCATCTGATCGTTGGCAACCAGCATCGCAGTGGGAACGATGCCCTCATTCAGCATTTGCATGGTTTGTTGAAAA
CCGGACATGGCACTCCAGTCGCCTTCCCGTTCCGCTATCGGCTGAATTTGATTGCGAGTGAGATATTTATGCCAGCCAGCCAGACGCAGACGCGCCGAGACAGAACT
TAATGGGCCCGCTAACAGCGCGATTTGCTGGTGACCCAATGCGACCAGATGCTCCACGCCCAGTCGCGTACCGTCTTCATGGGAGAAAATAATACTGTTGATGGGTG
TCTGGTCAGAGACATCAAGAAATAACGCCGGAACATTAGTGCAGGCAGCTTCCACAGCAATGGCATCCTGGTCATCCAGCGGATAGTTAATGATCAGCCCACTGACG
CGTTGCGCGAGAAGATTGTGCACCGCCGCTTTACAGGCTTCGACGCCGCTTCGTTCTACCATCGACACCACCACGCTGGCACCCAGTTGATCGGCGCGAGATTTAAT
CGCCGCGACAATTTGCGACGGCGCGTGCAGGGCCAGACTGGAGGTGGCAACGCCAATCAGCAACGACTGTTTGCCCGCCAGTTGTTGTGCCACGCGGTTGGGAATGT
AATTCAGCTCCGCCATCGCCGCTTCCACTTTTTCCCGCGTTTTCGCAGAAACGTGGCTGGCCTGGTTCACCACGCGGGAAACGGTCTGATAAGAGACACCGGCATAC
TCTGCGACATCGTATAACGTTACTGGTTTCACATTCACCACCCTGAATTGACTCTCTTCCGGGCGCTATCATGCCATACCGCGAAAGGTTTTGCGCCATTCGATGGT
GTCCGGGATCTCGACGCTCTCCCTTATGCGACTCCTGCATTAGGAAGCAGCCCAGTAGTAGGTTGAGGCCGTTGAGCACCGCCGCCGCAAGGAATGGTGCATGCAAG
GAGATGGCGCCCAACAGTCCCCCGGCCACGGGGCCTGCCACCATACCCACGCCGAAACAAGCGCTCATGAGCCCGAAGTGGCGAGCCCGATCTTCCCCATCGGTGAT
GTCGGCGATATAGGCGCCAGCAACCGCACCTGTGGCGCCGGTGATGCCGGCCACGATGCGTCCGGCGTAGAGGATCGAGATCTCGATCCCGCGAAATTAATACGACT
CACTATAGGGGAATTGTGAGCGGATAACAATTCCCCTCTAGAAATAATTTTGTTTAACTTTAAGAAGGAGATATACATATGCACCATCATCATCATCATTCTTCTGG
TGTAGATCTGGGTACCGAGAACCTGTACTTCCAATCCATGGCCCCAAAGAGAGTTGTGCAGCTGTCCCTGAAGATGCCTACCCATGCCGTGTGTGTGGTGGGAGTCG
AGGCACATGTGGACATTCACAGTGATGTGCCCAAGGGTGCCAACAGCTTCAGGGTCTCTGGAAGCTCCGGGGTGGAGGTCTTCATGGTCTACAACCGCACACGTGTG
AAAGAGCCCATAGGCAAGGCCCGTTGGCCGCTAGACACTGATGCAGACATGGTCGTATCTGTGGGCACAGCCAGTAAGGAATTAAAGGACTTCAAGGTGAGGGTCTC
CTACTTTGGGGAGCAGGAAGACCAAGCTCTGGGCCGCAGCGTGCTTTACCTCACTGGCGTCGATATTTCCCTTGAGGTTGACACAGGCCGCACAGGCAAGGTGAAGA
GGAGCCAAGGGGACAAGAAAACCTGGCGCTGGGGCCCTGAGGGCTATGGGGCTATCTTGCTGGTGAACTGTGACCGGGACAATCACAGGTCCGCAGAGCCTGACCTC
ACCCACAGCTGGCTGATGTCGCTGGCTGACCTGCAGGACATGTCCCCAATGCTGCTGAGCTGCAATGGCCCCGACAAGCTCTTCGACAGCCACAAGCTTGTCTTGAA
CGTGCCCTTTTCTGATTCCAAAAGAGTGAGGGTCTTCTGTGCCAGGGGTGGAATTCTCTCTCGGACTACAAACAGGTGCTGGGGCCCCAGTGTCTGTCCTATGAAG
TTGAGCGACAGCCAGGGGAGCAGGAGATCAAGTTCTATGTGGAGGGGCTGACCTTCCCCGATGCCGATTTCCTAGGGCTGGTTTCCCTCAGTGTCAGCCTGGTGGAC
CCGGGGACCCTGCCCGAGGTGACCCTCTTCACAGACACTGTGGGCTTCCGCATGGCCCCCTGGATCATGACGCCCAACACTCAGCCTCCTGAGGAGCTGTATGTGTG
CAGAGTGATGGACACTCATGGCTCCAATGAGAAATTCCTGGAGGACATGTCTTATCTGACATTGAAAGCCAACTGCAAGCTGACCATCTGCCCTCAAGTTGAAAATC
GAAATGACCGCTGGATCCAGGACGAGATGGAGTTTGGCTACATCGAGGCCCCTCACAAATCCTTCCCCGTGGTCTTTGACTCCCCCCGGAACAGGGGCCTGAAAGAT
TTCCCCTATAAGAGGATCCTGGGTCCTGACTTTGGATATGTTACCCGGGAGATCCCGCTCCCTGGTCCCTCCAGCCTTGACTCCTTCGGCAACCTGGACGTCAGCCC
GCCCGTCACGGTGGGCGGCACGGAATACCCCCTGGGCCGGATCCTCATCGGGAGCAGCTTCCCCAAGTCCGGTGGGCGGCAGATGGCCAGGGCAGTGCGGAACTTCC
TGAAGGCACAGCAGGTGCAGGCACCCGTGGAGCTCTACTCGGACTGGCTCTCTGTGGGCCATGTGGACGAGTTTCTGACCTTTGTGCCTACCTCTGACCAAAAGGGC
TTCCGGCTGCTCCTGGCTAGCCCCAGCGCTTGCCTCAAACTCTTCCAAGAGAAGAAAGAAGAGGGTTATGGGGAGGCAGCCCAGTTTGATGGGTTAAAACACCAGGC
AAAAAGAAGCATTAATGAGATGCTGGCAGACAGACACCTCCAGAGAGACAATCTTCATGCACAGAAATGCATTGACTGGAACCGTAATGTGCTGAAGCGGGAGCTGG
GCCTGGCAGAGAGTGACATCGTGGACATTCCCCAGCTCTTCTTCCTGAAAAACTTCTACGCGGAAGCCTTCTTCCCAGACATGGTTAACATGGTGGTCTTAGGCAAG
TACCTGGGCATCCCCAAGCCCTACGGGCCCATCATCAATGGCCGCTGCTGCCTGGAGGAGAAGGTGCAGTCCCTGCTGGAGCCTCTGGGCCTGCACTGCATCTTCAT
TGATGACTACTTGTCCTACCACGAGCTGCAGGGGGAGATCCACTGTGGCACCAACGTGCGCAGGAAGCCCTTTCCCTTCAAATGGTGGAACATGGTGCCCTGACAGT
AAAGGTGGATACGGATCCGAATTCGAGCTCCGTCGACAAGCTTGCGGCCGCACTCGAGCACCACCACCACCACCACTGAGATCCGGCTGCTAACAAAGCCCGAAAGG
AAGCTGAGTTGGCTGCTGCCACCGCTGAGCAATAACTAGCATAACCCCTTGGGGCCTCTAAACGGGTCTTGAGGGGTTTTTTGCTGAAAGGAGGAACTATATCCGGA
TTGGCGAATGGGACGCGCCCTGTAGCGGCGCATTAAGCGCGGCGGGTGTGGTGGTTACGCGCAGCGTGACCGCTACACTTGCCAGCGCCCTAGCGCCCGCTCCTTTC
GCTTTCTTCCCTTCCTTTCTCGCCACGTTCGCCGGCTTTCCCCGTCAAGCTCTAAATCGGGGGCTCCCTTTAGGGTTCCGATTTAGTGCTTTACGGCACCTCGACCC
CAAAAAACTTGATTAGGGTGATGGTTCACGTAGTGGGCCATCGCCCTGATAGACGGTTTTTCGCCCTTTGACGTTGGAGTCCACGTTCTTTAATAGTGGACTCTTGT
TCCAAACTGGAACAACACTCAACCCTATCTCGGTCTATTCTTTTGATTTATAAGGGATTTTGCCGATTTCGGCCTATTGGTTAAAAAATGAGCTGATTTAACAAAAA
TTTAACGCGAATTTTAACAAAATATTAACGCTTACAATTTAGGTGGCACTTTTCGGGGAAATGTGCGCGGAACCCCTATTTGTTTATTTTTCTAAATACATTCAAAT
ATGTATCCGCTCATGAATTAATTCTTAGAAAAACTCATCGAGCATCAAATGAAACTGCAATTTATTCATATCAGGATTATCAATACCATATTTTTGAAAAAGCCGTT
TCTGTAATGAAGGAGAAAACTCACCGAGGCAGTTCCATAGGATGGCAAGATCCTGGTATCGGTCTGCGATTCCGACTCGTCCAACATCAATACAACCTATTAATTTC
CCCTCGTCAAAAATAAGGTTATCAAGTGAGAAATCACCATGAGTGACGACTGAATCCGGTGAGAATGGCAAAAGTTTATGCATTTCTTTCCAGACTTGTTCAACAGG
CCAGCCATTACGCTCGTCATCAAAATCACTCGCATCAACCAAACCGTTATTCATTCGTGATTGCGCCTGAGCGAGACGAAATACGCGATCGCTGTTAAAAGGACAAT
TACAAACAGGAATCGAATGCAACCGGCGCAGGAACACTGCCAGCGCATCAACAATATTTTCACCTGAATCAGGATATTCTTCTAATACCTGGAATGCTGTTTTCCCG
GGGATCGCAGTGGTGAGTAACCATGCATCATCAGGAGTACGGATAAAATGCTTGATGGTCGGAAGAGGCATAAATTCCGTCAGCCAGTTTAGTCTGACCATCTCATC
TGTAACATCATTGGCAACGCTACCTTTGCCATGTTTCAGAAACAACTCTGGCGCATCGGGCTTCCCAT
```

His Tag
TEV protease cleavage site
PADI1

hPADI2 plasmid

GTCATCAAAATCACTCGCATCAACCAAACCGTTATTCATTCGTGATTGCGCCTGAGCGAGACGAAATACGCGATCGCTGTTAAAAGGACAATTACAAACAGGAATCG
AATGCAACCGGCGCAGGAACACTGCCAGCGCATCAACAATATTTTCACCTGAATCAGGATATTCTTCTAATACCTGGAATGCTGTTTTCCCGGGGATCGCAGTGGTG
AGTAACCATGCATCATCAGGAGTACGGATAAAATGCTTGATGGTCGGAAGAGGCATAAATTCCGTCAGCCAGTTTAGTCTGACCATCTCATCTGTAACATCATTGGC
AACGCTACCTTTGCCATGTTTCAGAAACAACTCTGGCGCATCGGGCTTCCCATACAATCGATAGATTGTCGCACCTGATTGCCCGACATTATCGCGAGCCCATTTAT
ACCCATATAAATCAGCATCCATGTTGGAATTTAATCGCGGCCTAGAGCAAGACGTTTCCCGTTGAATATGGCTCATAACACCCCTTGTATTACTGTTTATGTAAGCA
GACAGTTTTATTGTTCATGACCAAAATCCCTTAACGTGAGTTTTCGTTCCACTGAGCGTCAGACCCCGTAGAAAAGATCAAAGGATCTTCTTGAGATCCTTTTTTTC
TGCGCGTAATCTGCTGCTTGCAAACAAAAAAACCACCGCTACCAGCGGTGGTTTGTTTGCCGGATCAAGAGCTACCAACTCTTTTTCCGAAGGTAACTGGCTTCAGC
AGAGCGCAGATACCAAATACTGTCCTTCTAGTGTAGCCGTAGTTAGGCCACCACTTCAAGAACTCTGTAGCACCGCCTACATACCTCGCTCTGCTAATCCTGTTACC
AGTGGCTGCTGCCAGTGGCGATAAGTCGTGTCTTACCGGGTTGGACTCAAGACGATAGTTACCGGATAAGGCGCAGCGGTCGGGCTGAACGGGGGGTTCGTGCACAC
AGCCCAGCTTGGAGCGAACGACCTACACCGAACTGAGATACCTACAGCGTGAGCTATGAGAAAGCGCCACGCTTCCCGAAGGGAGAAAGGCGGACAGGTATCCGGTA
AGCGGCAGGGTCGGAACAGGAGAGCGCACGAGGGAGCTTCCAGGGGGAAACGCCTGGTATCTTTATAGTCCTGTCGGGTTTCGCCACCTCTGACTTGAGCGTCGATT
TTTGTGATGCTCGTCAGGGGGGCGGAGCCTATGGAAAAACGCCAGCAACGCGGCCTTTTTACGGTTCCTGGCCTTTTGCTGGCCTTTTGCTCACATGTTCTTTCCTG
CGTTATCCCCTGATTCTGTGGATAACCGTATTACCGCCTTTGAGTGAGCTGATACCGCTCGCCGCAGCCGAACGACCGAGCGCAGCGAGTCAGTGAGCGAGGAAGCG
GAAGAGCGCCTGATGCGGTATTTTCTCCTTACGCATCTGTGCGGTATTTCACACCGCAATGGTGCACTCTCAGTACAATCTGCTCTGATGCCGCATAGTTAAGCCAG
TATACACTCCGCTATCGCTACGTGACTGGGTCATGGCTGCGCCCCGACACCCGCCAACACCCGCTGACGCGCCCTGACGGGCTTGTCTGCTCCCGGCATCCGCTTAC
AGACAAGCTGTGACCGTCTCCGGGAGCTGCATGTGTCAGAGGTTTTCACCGTCATCACCGAAACGCGCGAGGCAGCTGCGGTAAAGCTCATCAGCGTGGTCGTGAAG
CGATTCACAGATGTCTGCCTGTTCATCCGCGTCCAGCTCGTTGAGTTTCTCCAGAAGCGTTAATGTCTGGCTTCTGATAAAGCGGGCCATGTTAAGGGCGGTTTTTT
CCTGTTTGGTCACTGATGCCTCCGTGTAAGGGGGATTTCTGTTCATGGGGGTAATGATACCGATGAAACGAGAGAGGATGCTCACGATACGGGTTACTGATGATGAA
CATGCCCGGTTACTGGAACGTTGTGAGGGTAAACAACTGGCGGTATGGATGCGGCGGGACCAGAGAAAAATCACTCAGGGTCAATGCCAGCGCTTCGTTAATACAGA
TGTAGGTGTTCCACAGGGTAGCCAGCAGCATCCTGCGATGCAGATCCGGAACATAATGGTGCAGGGCGCTGACTTCCGCGTTTCCAGACTTTACGAAACACGGAAAC
CGAAGACCATTCATGTTGTTGCTCAGGTCGCAGACGTTTTGCAGCAGCAGTCGCTTCACGTTCGCTCGCGTATCGGTGATTCATTCTGCTAACCAGTAAGGCAACCC
CGCCAGCCTAGCCGGGTCCTCAACGACAGGAGCACGATCATGCGCACCCGTGGGGCCGCCATGCCGGCGATAATGGCCTGCTTCTCGCCGAAACGTTTGGTGGCGGG
ACCAGTGACGAAGGCTTGAGCGAGGGCGTGCAAGATTCCGAATACCGCAAGCGACAGGCCGATCATCGTCGCGCTCCAGCGAAAGCGGTCCTCGCCGAAAATGACCC
AGAGCGCTGCCGGCACCTGTCCTACGAGTTGCATGATAAAGAAGACAGTCATAAGTGCGGCGACGATAGTCATGCCCCGCGCCCACCGGAAGGAGCTGACTGGGTTG
AAGGCTCTCAAGGGCATCGGTCGAGATCCCGGTGCCTAATGAGTGAGCTAACTTACATTAATTGCGTTGCGCTCACTGCCCGCTTTCCAGTCGGGAAACCTGTCGTG
CCAGCTGCATTAATGAATCGGCCAACGCGCGGGGAGAGGCGGTTTGCGTATTGGGCGCCAGGGTGGTTTTTCTTTTCACCAGTGAGACGGGCAACAGCTGATTGCCC
TTCACCGCCTGGCCCTGAGAGAGTTGCAGCAAGCGGTCCACGCTGGTTTGCCCCAGCAGGCGAAAATCCTGTTTGATGGTGGTTAACGGCGGGATATAACATGAGCT
GTCTTCGGTATCGTCGTATCCCACTACCGAGATATCCGCACCAACGCGCAGCCCGGACTCGGTAATGGCGCGCATTGCGCCCAGCGCCATCTGATCGTTGGCAACCA
GCATCGCAGTGGGAACGATGCCCTCATTCAGCATTTGCATGGTTTGTTGAAAACCGGACATGGCACTCCAGTCGCCTTCCCGTTCCGCTATCGGCTGAATTTGATTG
CGAGTGAGATATTTATGCCAGCCAGCCAGACGCAGACGCGCCGAGACAGAACTTAATGGGCCCGCTAACAGCGCGATTTGCTGGTGACCCAATGCGACCAGATGCTC
CACGCCCAGTCGCGTACCGTCTTCATGGGAGAAAATAATACTGTTGATGGGTGTCTGGTCAGAGACATCAAGAAATAACGCCGGAACATTAGTGCAGGCAGCTTCCA
CAGCAATGGCATCCTGGTCATCCAGCGGATAGTTAATGATCAGCCCACTGACGCGTTGCGCGAGAAGATTGTGCACCGCCGCTTTACAGGCTTCGACGCCGCTTCGT
TCTACCATCGACACCACCACGCTGGCACCCAGTTGATCGGCGCGAGATTTAATCGCCGCGACAATTTGCGACGGCGCGTGCAGGGCCAGACTGGAGGTGGCAACGCC
AATCAGCAACGACTGTTTGCCCGCCAGTTGTTGTGCCACGCGGTTGGGAATGTAATTCAGCTCCGCCATCGCCGCTTCCACTTTTTCCCGCGTTTTCGCAGAAACGT
GGCTGGCCTGGTTCACCACGCGGGAAACGGTCTGATAAGAGACACCGGCATACTCTGCGACATCGTATAACGTTACTGGTTTCACATTCACCACCCTGAATTGACTC
TCTTCCGGGCGCTATCATGCCATACCGCGAAAGGTTTTGCGCCATTCGATGGTGTCCGGGATCTCGACGCTCTCCCTTATGCGACTCCTGCATTAGGAAGCAGCCCA
GTAGTAGGTTGAGGCCGTTGAGCACCGCCGCCGCAAGGAATGGTGCATGCAAGGAGATGGCGCCCAACAGTCCCCCGGCCACGGGGCCTGCCACCATACCCACGCCG
AAACAAGCGCTCATGAGCCCGAAGTGGCGAGCCCGATCTTCCCCATCGGTGATGTCGGCGATATAGGCGCCAGCAACCGCACCTGTGGCGCCGGTGATGCCGGCCAC
GATGCGTCCGGCGTAGAGGATCGAGATCTCGATCCCGCGAAATTAATACGACTCACTATAGGGGAATTGTGAGCGGATAACAATTCCCCTCTAGAAATAATTTTGTT
TAACTTTAAGAAGGAGATATACCATGAGCGGCCTGAACGATATTTTTGAAGCGCAGAAAATTGAATGGCATGAAGGCAGCGCTGGAGGTTCAGGTCATCACCACCAT
CATCACGGTAGTGGTGGAATGCTGCGCGAGCGGACCGTGCGGCTGCAGTACGGGAGCCGCGTGGAGGCGGTGTACGTGCTGGGCACCTACCTCTGGACCGATGTCTA
CAGCGCGGCCCCAGCCGGGGCCCAAACCTTCAGCCTGAAGCACTCGGAACACGTGTGGGTGGAGGTGGTGCGTGATGGGGAGGCTGAGGAGGTGGCCACCAATGGCA
AGCAGCGCTGGCTTCTCTCGCCCAGCACCACCCTGCGGGTCACCATGAGCCAGGCGAGCACCGAGGCCAGCAGTGACAAGGTCACCGTCAACTACTATGACGAGGAA
GGGAGCATTCCCATCGACCAGGCGGGGCTCTTCCTCACAGCCATTGAGATCTCCCTGGATGTGGACGCAGACCGGGATGGTGTGGTGGAGAAGAACAACCCAAAGAA
GGCATCCTGGACCTGGGGCCCCGAGGGCCAGGGGGCCATCCTGCTGGTGAACTGTGACCGAGAGACACCCTGGTTGCCCAAGGAGGACTGCCGTGATGAGAAGGTCT
ACAGCAAGGAAGATCTCAAGGACATGTCCCAGATGATCCTGCGGACCAAAGGCCCCGACCGCCTCCCCGCCGGATACGAGATAGTTCTGTACATTTCCATGTCAGAC
TCAGACAAAGTGGGCGTGTTCTACGTGGAGAACCCGTTCTTCGGCCAACGCTATATCCACATCCTGGGCCGGCGGAAGCTCTACCATGTGGTCAAGTACACGGGTGG
CTCCGCGGAGCTGCTGTTCTTCGTGGAAGGCCTCTGTTTCCCCGACGAGGGCTTCTCAGGCCTGGTCTCCATCCATGTCAGCCTGCTGGAGTACATGGCCCAGGACA
TTCCCCTGACTCCCATCTTCACGGACACCGTGATATTCCGGATTGCTCCGTGGATCATGACCCCCAACATCCTGCCTCCCGTGTCGGTGTTTGTGTGCTGCATGAAG
GATAATTACCTGTTCCTGAAAGAGGTGAAGAACCTTGTGGAGAAAACCAACTGTGAGCTGAAGGTCTGCTTCCAGTACCTAAACCGAGGCGATCGCTGGATCCAGGA
TGAAATTGAGTTTGGCTACATCGAGGCCCCCCATAAAGGCTTCCCCGTGGTGCTGGACTCTCCCCGAGATGGAAACCTAAAGGACTTCCCTGTGAAGGAGCTCCTGG
GCCCAGATTTTGGCTACGTGACCCGGGAGCCCCTCTTTGAGTCTGTCACCAGCCTTGACTCATTTGGAAACCTGGAGGTCAGTCCCCCAGTGACCGTGAATGGCAAG
ACATACCCGCTTGGCCGCATCCTCATCGGGAGCAGCTTTCCTCTGTCTGGTGGTCGGAGGATGACCAAGGTGGTGCGTGACTTCCTGAAGGCCCAGCAGGTGCAGGC
ACCCGTGGAGCTCTACTCAGACTGGCTGACTGTGGGCCACGTGGATGAGTTCATGTCCTTTGTCCCCATCCCCGGCACAAAGAAATTCCTGCTACTCATGGCCAGCA
CCTCGGCCTGCTACAAGCTCTTCCGAGAGAAGCAGAAGGACGGCCATGGAGAGGCCATCATGTTCAAAGGCTTGGGTGGGATGAGCAGCAAGCGAATCACCATCAAC
AAGATTCTGTCCAACGAGAGCCTTGTGCAGGAGAACCTGTACTTCCAGCGCTGCCTGGACTGGAACCGTGACATCCTCAAGAAGGAGCTGGGACTGACAGAGCAGGA
CATCATTGACCTGCCCGCTCTGTTCAAGATGGACGAGGACCACCGTGCCAGAGCCTTCTTCCCAAACATGGTGAACATGATCGTGCTGGACAAGGACCTGGGCATCC
CCAAGCCATTCGGGCCACAGGTTGAGGAGGAATGCTGCCTGGAGATGCACGTGCGTGGCCTCCTGGAGCCCCTGGGCCTCGAATGCACCTTCATCGACGACATTTCT
GCCTACCACAAATTTCTGGGGGAAGTCCACTGTGGCACCAACGTCCGCAGGAAGCCCTTCACCTTCAAGTGGTGGCACATGGTGCCCTAAGGTGGAAGTGGTCATCA
CCACCATCATCACTGATGACGAAGCTTGCGGCCGCACTCGAGCACCACCACCACCACCACTGAGATCCGGCTGCTAACAAAGCCCGAAAGGAAGCTGAGTTGGCTGC
TGCCACCGCTGAGCAATAACTAGCATAACCCCTTGGGGCCTCTAAACGGGTCTTGAGGGGTTTTTTGCTGAAAGGAGGAACTATATCCGGATTGGCGAATGGGACGC
GCCCTGTAGCGGCGCATTAAGCGCGGCGGGTGTGGTGGTTACGCGCAGCGTGACCGCTACACTTGCCAGCGCCCTAGCGCCCGCTCCTTTCGCTTTCTTCCCTTCCT
TTCTCGCCACGTTCGCCGGCTTTCCCCGTCAAGCTCTAAATCGGGGGCTCCCTTTAGGGTTCCGATTTAGTGCTTTACGGCACCTCGACCCCAAAAAACTTGATTAG
GGTGATGGTTCACGTAGTGGGCCATCGCCCTGATAGACGGTTTTTCGCCCTTTGACGTTGGAGTCCACGTTCTTTAATAGTGGACTCTTGTTCCAAACTGGAACAAC
ACTCAACCCTATCTCGGTCTATTCTTTTGATTTATAAGGGATTTTGCCGATTTCGGCCTATTGGTTAAAAAATGAGCTGATTTAACAAAAATTTAACGCGAATTTTA
ACAAAATATTAACGCTTACAATTTAGGTGGCACTTTTCGGGGAAATGTGCGCGGAACCCCTATTTGTTTATTTTTCTAAATACATTCAAATATGTATCCGCTCATGA
ATTAATTCTTAGAAAAACTCATCGAGCATCAAATGAAACTGCAATTTATTCATATCAGGATTATCAATACCATATTTTTGAAAAAGCCGTTTCTGTAATGAAGGAGA
AAACTCACCGAGGCAGTTCCATAGGATGGCAAGATCCTGGTATCGGTCTGCGATTCCGACTCGTCCAACATCAATACAACCTATTAATTTCCCCTCGTCAAAAATAA
GGTTATCAAGTGAGAAATCACCATGAGTGACGACTGAATCCGGTGAGAATGGCAAAAGTTTATGCATTTCTTTCCAGACTTGTTCAACAGGCCAGCCATTACGCTC

His Tag
Avi Tag
PADI2

hPADI3 plasmid

CCCGCTAACAGCGCGATTTGCTGGTGACCCAATGCGACCAGATGCTCCACGCCCAGTCGCGTACCGTCTTCATGGGAGAAAATAATACTGTTGATGGGTGTCTGGTC
AGAGACATCAAGAAATAACGCCGGAACATTAGTGCAGGCAGCTTCCACAGCAATGGCATCCTGGTCATCCAGCGGATAGTTAATGATCAGCCCACTGACGCGTTGCG
CGAGAAGATTGTGCACCGCCGCTTTACAGGCTTCGACGCCGCTTCGTTCTACCATCGACACCACCACGCTGGCACCCAGTTGATCGGCGCGAGATTTAATCGCCGCG
ACAATTTGCGACGGCGCGTGCAGGGCCAGACTGGAGGTGGCAACGCCAATCAGCAACGACTGTTTGCCCGCCAGTTGTTGTGCCACGCGGTTGGGAATGTAATTCAG
CTCCGCCATCGCCGCTTCCACTTTTTCCCGCGTTTTCGCAGAAACGTGGCTGGCCTGGTTCACCACGCGGGAAACGGTCTGATAAGAGACACCGGCATACTCTGCGA
CATCGTATAACGTTACTGGTTTCACATTCACCACCCTGAATTGACTCTCTTCCGGGCGCTATCATGCCATACCGCGAAAGGTTTTGCGCCATTCGATGGTGTCCGGG
ATCTCGACGCTCTCCCTTATGCGACTCCTGCATTAGGAAGCAGCCCAGTAGTAGGTTGAGGCCGTTGAGCACCGCCGCCGCAAGGAATGGTGCATGCAAGGAGATGG
CGCCCAACAGTCCCCCGGCCACGGGGCCTGCCACCATACCCACGCCGAAACAAGCGCTCATGAGCCCGAAGTGGCGAGCCCGATCTTCCCCATCGGTGATGTCGGCG
ATATAGGCGCCAGCAACCGCACCTGTGGCGCCGGTGATGCCGGCCACGATGCGTCCGGCGTAGAGGATCGAGATCTCGATCCCGCGAAATTAATACGACTCACTATA
GGGGAATTGTGAGCGGATAACAATTCCCCTCTAGAAATAATTTTGTTTAACTTTAAGAAGGAGATATACATATGCACCATCATCATCATCATTCTTCTGGTGTAGAT
CTGGGTACCGAGAACCTGTACTTCCAATCCATGTCGCTGCAGAGAATCGTGCGTGTGTCCCTGGAGCATCCCACCAGCGCGGTGTGTGTGGCTGGCGTGGAGACCCT
CGTGGACATTTATGGGTCAGTGCCTGAGGGCACAGAAATGTTTGAGGTCTATGGGACGCCTGGCGTGGACATCTACATCTCTCCCAACATGGAGAGGGGCCGGGAGC
GTGCAGACACCAGGCGGTGGCGCTTTGACGCGACTTTGGAGATCATCGTGGTCATGAACTCCCCCAGCAATGACCTCAACGACAGCCATGTTCAGATTTCCTACCAC
TCCAGCCATGAGCCTCTGCCCCTGGCCTATGCGGTGCTCTACCTCACCTGTGTTGACATCTCTCTGGATTGCGACCTGAACTGTGAGGGAAGGCAGGACAGGAACTT
TGTAGACAAGCGGCAGTGGGTCTGGGGGCCCAGTGGGTATGGCGGCATCTTGCTGGTGAACTGTGACCGTGATGATCCGAGCTGTGATGTCCAGGACAATTGTGACC
AGCACGTGCACTGCCTGCAAGACCTGGAAGACATGTCTGTCATGGTCCTGCGGACGCAGGGCCCTGCAGCCCTCTTTGATGACCACAAACTTGTCCTCCATACCTCC
AGCTATGATGCCAAACGGGCACAGGTCTTCCACATCTGCGGTCCTGAGGATGTGTGTGAGGCCTATAGGCATGTGCTGGGCCAAGATAAGGTGTCCTATGAGGTACC
CCGCTTGCATGGGGATGAGGAGCGCTTCTTCGTGGAAGGCCTGTCCTTCCCTGATGCCGGCTTCACAGGACTCATCTCCTTCCATGTCACTCTGCTGGACGACTCCA
ACGAGGATTTCTCGGCATCCCCTATCTTCACTGACACTGTGGTGTTCCGAGTGGCACCCTGGATCATGACGCCCAGCACTCTGCCACCCCTAGAGGTGTATGTGTGC
CGTGTGAGGAACAACACGTGTTTTGTGGATGCGGTGGCAGAGCTGGCCAGGAAGGCCGGCTGCAAGCTGACCATCTGCCCACAGGCCGAGAACCGCAACGACCGCTG
GATCCAGGATGAGATGGAGCTGGGCTACGTTCAGGCGCCGCACAAGACCCTCCCGGTGGTCTTTGACTCCCCAAGGAATGGGGAACTGCAGGATTTCCCTTACAAAA
GAATCCTGGGTCCAGATTTTGGTTACGTGACTCGGGAACCACGCGACAGGTCTGTGAGTGGCCTGGACTCCTTTGGGAACCTGGAGGTCAGCCCTCCAGTGGTGGCC
AATGGGAAAGAGTACCCCCTGGGGAGGATCCTCATTGGGGGCAACCTGCCTGGGTCAAGTGGCCGCAGGGTCACCCAGGTGGTGCGGGACTTCCTCCATGCCCAGAA
GGTGCAGCCCCCCGTGGAGCTCTTTGTGGACTGGTTGGCCGTGGGCCATGTGGATGAGTTTCTGAGCTTTGTCCCTGCCCCCGATGGGAAGGGCTTCCGGATGCTCC
TGGCCAGCCCTGGGGCCTGCTTCAAGCTCTTCCAGGAAAAGCAGAAGTGTGGCCACGGGAGGGCCCTCCTGTTCCAGGGGGTTGTTGATGATGAGCAGGTCAAGACC
ATCTCCATCAACCAGGTGCTCTCCAATAAAGACCTCATCAACTACAATAAGTTTGTGCAGAGCTGCATCGACTGGAACCGTGAGGTGCTGAAGCGGGAGCTGGGCCT
GGCAGAGTGTGACATCATTGACATCCCACAGCTCTTCAAGACCGAGAGGAAAAAAGCAACGGCCTTCTTCCCTGACTTGGTGAACATGCTGGTGCTGGGGAAGCACC
TGGGCATCCCCAAGCCCTTTGGGCCCATCATCAATGGCTGCTGCTGCCTGGAGGAGAAGGTGCGGTCCCTGCTGGAGCCTCTGGGCCTCCACTGCACCTTCATTGAT
GACTTCACTCCATACCACATGCTGCATGGGGAGGTGCACTGTGGCACCAATGTGTGCAGAAAGCCCTTCTCTTTCAAGTGGTGGAACATGGTGCCCTGACAGTAAAG
GTGGATACGGATCCGAATTCGAGCTCCGTCGACAAGCTTGCGGCCGCACTCGAGCACCACCACCACCACCACTGAGATCCGGCTGCTAACAAAGCCCGAAAGGAAGC
TGAGTTGGCTGCTGCCACCGCTGAGCAATAACTAGCATAACCCCTTGGGGCCTCTAAACGGGTCTTGAGGGGTTTTTTGCTGAAAGGAGGAACTATATCCGGATTGG
CGAATGGGACGCGCCCTGTAGCGGCGCATTAAGCGCGGCGGGTGTGGTGGTTACGCGCAGCGTGACCGCTACACTTGCCAGCGCCCTAGCGCCCGCTCCTTTCGCTT
TCTTCCCTTCCTTTCTCGCCACGTTCGCCGGCTTTCCCCGTCAAGCTCTAAATCGGGGGCTCCCTTTAGGGTTCCGATTTAGTGCTTTACGGCACCTCGACCCCAAA
AAACTTGATTAGGGTGATGGTTCACGTAGTGGGCCATCGCCCTGATAGACGGTTTTTCGCCCTTTGACGTTGGAGTCCACGTTCTTTAATAGTGGACTCTTGTTCCA
AACTGGAACAACACTCAACCCTATCTCGGTCTATTCTTTTGATTTATAAGGGATTTTGCCGATTTCGGCCTATTGGTTAAAAAATGAGCTGATTTAACAAAAATTTA
ACGCGAATTTTAACAAAATATTAACGCTTACAATTTAGGTGGCACTTTTCGGGGAAATGTGCGCGGAACCCCTATTTGTTTATTTTTCTAAATACATTCAAATATGT
ATCCGCTCATGAATTAATTCTTAGAAAAACTCATCGAGCATCAAATGAAACTGCAATTTATTCATATCAGGATTATCAATACCATATTTTTGAAAAAGCCGTTTCTG
TAATGAAGGAGAAAACTCACCGAGGCAGTTCCATAGGATGGCAAGATCCTGGTATCGGTCTGCGATTCCGACTCGTCCAACATCAATACAACCTATTAATTTCCCCT
CGTCAAAAATAAGGTTATCAAGTGAGAAATCACCATGAGTGACGACTGAATCCGGTGAGAATGGCAAAAGTTTATGCATTTCTTTCCAGACTTGTTCAACAGGCCAG
CCATTACGCTCGTCATCAAAATCACTCGCATCAACCAAACCGTTATTCATTCGTGATTGCGCCTGAGCGAGACGAAATACGCGATCGCTGTTAAAAGGACAATTACA
AACAGGAATCGAATGCAACCGGCGCAGGAACACTGCCAGCGCATCAACAATATTTTCACCTGAATCAGGATATTCTTCTAATACCTGGAATGCTGTTTTCCCGGGGA
TCGCAGTGGTGAGTAACCATGCATCATCAGGAGTACGGATAAAATGCTTGATGGTCGGAAGAGGCATAAATTCCGTCAGCCAGTTTAGTCTGACCATCTCATCTGTA
ACATCATTGGCAACGCTACCTTTGCCATGTTTCAGAAACAACTCTGGCGCATCGGGCTTCCCATACAATCGATAGATTGTCGCACCTGATTGCCCGACATTATCGCG
AGCCCATTTATACCCATATAAATCAGCATCCATGTTGGAATTTAATCGCGGCCTAGAGCAAGACGTTTCCCGTTGAATATGGCTCATAACACCCCTTGTATTACTGT
TTATGTAAGCAGACAGTTTTATTGTTCATGACCAAAATCCCTTAACGTGAGTTTTCGTTCCACTGAGCGTCAGACCCCGTAGAAAAGATCAAAGGATCTTCTTGAGA
TCCTTTTTTTCTGCGCGTAATCTGCTGCTTGCAAACAAAAAAACCACCGCTACCAGCGGTGGTTTGTTTGCCGGATCAAGAGCTACCAACTCTTTTTCCGAAGGTAA
CTGGCTTCAGCAGAGCGCAGATACCAAATACTGTCCTTCTAGTGTAGCCGTAGTTAGGCCACCACTTCAAGAACTCTGTAGCACCGCCTACATACCTCGCTCTGCTA
ATCCTGTTACCAGTGGCTGCTGCCAGTGGCGATAAGTCGTGTCTTACCGGGTTGGACTCAAGACGATAGTTACCGGATAAGGCGCAGCGGTCGGGCTGAACGGGGGG
TTCGTGCACACAGCCCAGCTTGGAGCGAACGACCTACACCGAACTGAGATACCTACAGCGTGAGCTATGAGAAAGCGCCACGCTTCCCGAAGGGAGAAAGGCGGACA
GGTATCCGGTAAGCGGCAGGGTCGGAACAGGAGAGCGCACGAGGGAGCTTCCAGGGGGAAACGCCTGGTATCTTTATAGTCCTGTCGGGTTTCGCCACCTCTGACTT
GAGCGTCGATTTTTGTGATGCTCGTCAGGGGGGCGGAGCCTATGGAAAAACGCCAGCAACGCGGCCTTTTTACGGTTCCTGGCCTTTTGCTGGCCTTTTGCTCACAT
GTTCTTTCCTGCGTTATCCCCTGATTCTGTGGATAACCGTATTACCGCCTTTGAGTGAGCTGATACCGCTCGCCGCAGCCGAACGACCGAGCGCAGCGAGTCAGTGA
GCGAGGAAGCGGAAGAGCGCCTGATGCGGTATTTTCTCCTTACGCATCTGTGCGGTATTTCACACCGCAATGGTGCACTCTCAGTACAATCTGCTCTGATGCCGCAT
AGTTAAGCCAGTATACACTCCGCTATCGCTACGTGACTGGGTCATGGCTGCGCCCCGACACCCGCCAACACCCGCTGACGCGCCCTGACGGGCTTGTCTGCTCCCGG
CATCCGCTTACAGACAAGCTGTGACCGTCTCCGGGAGCTGCATGTGTCAGAGGTTTTCACCGTCATCACCGAAACGCGCGAGGCAGCTGCGGTAAAGCTCATCAGCG
TGGTCGTGAAGCGATTCACAGATGTCTGCCTGTTCATCCGCGTCCAGCTCGTTGAGTTTCTCCAGAAGCGTTAATGTCTGGCTTCTGATAAAGCGGGCCATGTTAAG
GGCGGTTTTTTCCTGTTTGGTCACTGATGCCTCCGTGTAAGGGGGATTTCTGTTCATGGGGGTAATGATACCGATGAAACGAGAGAGGATGCTCACGATACGGGTTA
CTGATGATGAACATGCCCGGTTACTGGAACGTTGTGAGGGTAAACAACTGGCGGTATGGATGCGGCGGGACCAGAGAAAAATCACTCAGGGTCAATGCCAGCGCTTC
GTTAATACAGATGTAGGTGTTCCACAGGGTAGCCAGCAGCATCCTGCGATGCAGATCCGGAACATAATGGTGCAGGGCGCTGACTTCCGCGTTTCCAGACTTTACGA
AACACGGAAACCGAAGACCATTCATGTTGTTGCTCAGGTCGCAGACGTTTTGCAGCAGCAGTCGCTTCACGTTCGCTCGCGTATCGGTGATTCATTCTGCTAACCAG
TAAGGCAACCCCGCCAGCCTAGCCGGGTCCTCAACGACAGGAGCACGATCATGCGCACCCGTGGGGCCGCCATGCCGGCGATAATGGCCTGCTTCTCGCCGAAACGT
TTGGTGGCGGGACCAGTGACGAAGGCTTGAGCGAGGGCGTGCAAGATTCCGAATACCGCAAGCGACAGGCCGATCATCGTCGCGCTCCAGCGAAAGCGGTCCTCGCC
GAAAATGACCCAGAGCGCTGCCGGCACCTGTCCTACGAGTTGCATGATAAAGAAGACAGTCATAAGTGCGGCGACGATAGTCATGCCCCGCGCCCACCGGAAGGAGC
TGACTGGGTTGAAGGCTCTCAAGGGCATCGGTCGAGATCCCGGTGCCTAATGAGTGAGCTAACTTACATTAATTGCGTTGCGCTCACTGCCCGCTTTCCAGTCGGGA
AACCTGTCGTGCCAGCTGCATTAATGAATCGGCCAACGCGCGGGGAGAGGCGGTTTGCGTATTGGGCGCCAGGGTGGTTTTTCTTTTCACCAGTGAGACGGGCAACA
GCTGATTGCCCTTCACCGCCTGGCCCTGAGAGAGTTGCAGCAAGCGGTCCACGCTGGTTTGCCCCAGCAGGCGAAAATCCTGTTTGATGGTGGTTAACGGCGGGATA
TAACATGAGCTGTCTTCGGTATCGTCGTATCCCACTACCGAGATATCCGCACCAACGCGCAGCCCGGACTCGGTAATGGCGCGCATTGCGCCCAGCGCCATCTGATC
GTTGGCAACCAGCATCGCAGTGGGAACGATGCCCTCATTCAGCATTTGCATGGTTTGTTGAAAACCGGACATGGCACTCCAGTCGCCTTCCCGTTCCGCTATCGGCT
GAATTTGATTGCGAGTGAGATATTTATGCCAGCCAGCCAGACGCAGACGCGCCGAGACAGAACTTAATGGG

His Tag
TEV protease cleavage site
PADI3

hPADI4 plasmid

```
CCCGCTAACAGCGCGATTTGCTGGTGACCCAATGCGACCAGATGCTCCACGCCCAGTCGCGTACCGTCTTCATGGGAGAAAATAATACTGTTGATGGGTGTCTGGTC
AGAGACATCAAGAAATAACGCCGGAACATTAGTGCAGGCAGCTTCCACAGCAATGGCATCCTGGTCATCCAGCGGATAGTTAATGATCAGCCCACTGACGCGTTGCG
CGAGAAGATTGTGCACCGCCGCTTTACAGGCTTCGACGCCGCTTCGTTCTACCATCGACACCACCACGCTGGCACCCAGTTGATCGGCGCGAGATTTAATCGCCGCG
ACAATTTGCGACGGCGCGTGCAGGGCCAGACTGGAGGTGGCAACGCCAATCAGCAACGACTGTTTGCCCGCCAGTTGTTGTGCCACGCGGTTGGGAATGTAATTCAG
CTCCGCCATCGCCGCTTCCACTTTTTCCCGCGTTTTCGCAGAAACGTGGCTGGCCTGGTTCACCACGCGGGAAACGGTCTGATAAGAGACACCGGCATACTCTGCGA
CATCGTATAACGTTACTGGTTTCACATTCACCACCCTGAATTGACTCTCTTCCGGGCGCTATCATGCCATACCGCGAAAGGTTTTGCGCCATTCGATGGTGTCCGGG
ATCTCGACGCTCTCCCTTATGCGACTCCTGCATTAGGAAGCAGCCCAGTAGTAGGTTGAGGCCGTTGAGCACCGCCGCCGCAAGGAATGGTGCATGCAAGGAGATGG
CGCCCAACAGTCCCCCGGCCACGGGGCCTGCCACCATACCCACGCCGAAACAAGCGCTCATGAGCCCGAAGTGGCGAGCCCGATCTTCCCCATCGGTGATGTCGGCG
ATATAGGCGCCAGCAACCGCACCTGTGGCGCCGGTGATGCCGGCCACGATGCGTCCGGCGTAGAGGATCGAGATCTCGATCCCGCGAAATTAATACGACTCACTATA
GGGGAATTGTGAGCGGATAACAATTCCCCTCTAGAAATAATTTTGTTTAACTTTAAGAAGGAGATATACCATGAGCGGCCTGAACGATATTTTTGAAGCGCAGAAAA
TTGAATGGCATGAAGGCAGCGCTGGAGGTTCAGGTCATCACCACCATCATCACGGTAGTGGTGGAATGGCCCAGGGGACATTGATCCGTGTGACCCCAGAGCAGCCC
ACCCATGCCGTGTGTGTGCTGGGCACCTTGACTCAGCTTGACATCTGCAGCTCTGCCCCTGAGGACTGCACGTCCTTCAGCATCAACGCCTCCCCAGGGGTGGTCGT
GGATATTGCCCACAGCCCTCCAGCCAAGAAGAAATCCACAGGTTCCTCCACATGGCCCCTGGACCCTGGGGTAGAGGTGACCCTGACGATGAAAGCGGCCAGTGGTA
GCACAGGCGACCAGAAGGTTCAGATTTCATACTACGGACCCAAGACTCCACCAGTCAAAGCTCTACTCTACCTCACCGCGGTGGAAATCTCCCTGTGCGCAGACATC
ACCCGCACCGGCAAAGTGAAGCCAACCAGAGCTGTGAAAGATcAGAGGACCTGGACCTGGGGCCCTTGTGGACAGGGTGCCATCCTGCTGGTGAACTGTGACAGAGA
CAATCTCGAATCTTCTGCCATGGACTGCGAGGATGATGAAGTGCTTGACAGCGAAGACCTGCAGGACATGTCGCTGATGACCCTGAGCACGAAGACCCCCAAGGACT
TCTTCACAAACCATACACTGGTGCTCCACGTGGCCAGGTCTGAGATGGACAAAGTGAGGGTGTTTCAGGCCACACGGGGCAAACTGTCCTCCAAGTGCAGCGTAGTC
TTGGGTCCCAAGTGGCCCTCTCACTACCTGATGGTCCCCGGTGGAAAGCACAACATGGACTTCTACGTGGAGGCCCTCGCTTTCCCGGACACCGACTTCCCGGGGCT
CATTACCCTCACCATCTCCCTGCTGGACACGTCCAACCTGGAGCTCCCCGAGGCTGTGGTGTTCCAAGACAGCGTGGTCTTCCGCGTGGCGCCCTGGATcATGACCC
CCAACACCCAGCCCCCGCAGGAGGTGTACGCGTGCAGTATTTTTGAAAATGAGGACTTCCTGAAGTCAGTGACTACTCTGGCCATGAAAGCCAAGTGCAAGCTGACC
ATCTGCCCTGAGGAGGAGAACATGGATGACCAGTGGATGCAGGATGAAATGGAGATcGGCTACATCCAAGCCCCACACAAAACGCTGCCCGTGGTCTTCGACTCTCC
AAGGAACAGAGGCCTGAAGGAGTTTCCCATCAAACGAGTGATGGGTCCAGATTTTGGCTATGTAACTCGAGGGCCCCAAACAGGGGGTATCAGTGGACTGGACTCCT
TTGGGAACCTGGAAGTGAGCCCCCCAGTCACAGTCAGGGGCAAGGAATACCCGCTGGGCAGGATTCTCTTCGGGGACAGCTGTTATCCCAGCAATGACAGCCGGCAG
ATGCACCAGGCCCTACAGGACTTCCTCAGTGCCCAGCAGGTGCAGGCCCCTGTGAAGCTCTATTCTGACTGGCTGTCCGTGGGCCACGTGGACGAGTTCCTGAGCTT
TGTGCCAGCACCCGACAGGAAGGGCTTCCGGCTGCTCCTGGCCAGCCCCAGGTCCTGCTACAAACTGTTCCAGGAGCAGCAGAATGAGGGCCACGGGGAGGCCCTGC
TGTTCGAAGGGATCAAGAAAAAAAAACAGCAGAAAATAAAGAACATTCTGTCAAACAAGACATTGAGAGAACATAATTCATTTGTGGAGAGATGCATCGACTGGAAC
CGCGAGCTGCTGAAGCGGGAGCTGGGCCTGGCCGAGAGTGACATCATTGACATCCCGCAGCTCTTCAAGCTCAAAGAGTTCTCTAAGGCGGAAGCTTTTTTCCCCAA
CATGGTGAACATGCTGGTGCTAGGGAAGCACCTGGGCATCCCCAAGCCCTTCGGGCCCGTCATCAACGGCCGCTGCTGCCTGGAGGAGAAGGTGTGTTCCCTGCTGG
AGCCACTGGGCCTCCAGTGCACCTTCATCAACGACTTCTTCACCTACCACATCAGGCATGGGGAGGTGCACTGCGGCACCAACGTGCGCAGAAAGCCCTTCTCCTTC
AAGTGGTGGAACATGGTGCCCTGAGGTGGAAGTGGTCATCACCACCATCATCACTGATGACGAAGCTTGCGGCCGCACTCGAGCACCACCACCACCACCACTGAGAT
CCGGCTGCTAACAAAGCCCGAAAGGAAGCTGAGTTGGCTGCTGCCACCGCTGAGCAATAACTAGCATAACCCCTTGGGGCCTCTAAACGGGTCTTGAGGGGTTTTTT
GCTGAAAGGAGGAACTATATCCGGATTGGCGAATGGGACGCGCCCTGTAGCGGCGCATTAAGCGCGGCGGGTGTGGTGGTTACGCGCAGCGTGACCGCTACACTTGC
CAGCGCCCTAGCGCCCGCTCCTTTCGCTTTCTTCCCTTCCTTTCTCGCCACGTTCGCCGGCTTTCCCCGTCAAGCTCTAAATCGGGGGCTCCCTTTAGGGTTCCGAT
TTAGTGCTTTACGGCACCTCGACCCCAAAAAACTTGATTAGGGTGATGGTTCACGTAGTGGGCCATCGCCCTGATAGACGGTTTTTCGCCCTTTGACGTTGGAGTCC
ACGTTCTTTAATAGTGGACTCTTGTTCCAAACTGGAACAACACTCAACCCTATCTCGGTCTATTCTTTTGATTTATAAGGGATTTTGCCGATTTCGGCCTATTGGTT
AAAAAATGAGCTGATTTAACAAAAATTTAACGCGAATTTTAACAAAATATTAACGCTTACAATTTAGGTGGCACTTTTCGGGGAAATGTGCGCGGAACCCCTATTTG
TTTATTTTTCTAAATACATTCAAATATGTATCCGCTCATGAATTAATTCTTAGAAAAACTCATCGAGCATCAAATGAAACTGCAATTTATTCATATCAGGATTATCA
ATACCATATTTTTGAAAAAGCCGTTTCTGTAATGAAGGAGAAAACTCACCGAGGCAGTTCCATAGGATGGCAAGATCCTGGTATCGGTCTGCGATTCCGACTCGTCC
AACATCAATACAACCTATTAATTTCCCCTCGTCAAAAATAAGGTTATCAAGTGAGAAATCACCATGAGTGACGACTGAATCCGGTGAGAATGGCAAAAGTTTATGCA
TTTCTTTCCAGACTTGTTCAACAGGCCAGCCATTACGCTCGTCATCAAAATCACTCGCATCAACCAAACCGTTATTCATTCGTGATTGCGCCTGAGCGAGACGAAAT
ACGCGATCGCTGTTAAAAGGACAATTACAAACAGGAATCGAATGCAACCGGCGCAGGAACACTGCCAGCGCATCAACAATATTTTCACCTGAATCAGGATATTCTTC
TAATACCTGGAATGCTGTTTTCCCGGGGATCGCAGTGGTGAGTAACCATGCATCATCAGGAGTACGGATAAAATGCTTGATGGTCGGAAGAGGCATAAATTCCGTCA
GCCAGTTTAGTCTGACCATCTCATCTGTAACATCATTGGCAACGCTACCTTTGCCATGTTTCAGAAACAACTCTGGCGCATCGGGCTTCCCATACAATCGATAGATT
GTCGCACCTGATTGCCCGACATTATCGCGAGCCCATTTATACCCATATAAATCAGCATCCATGTTGGAATTTAATCGCGGCCTAGAGCAAGACGTTTCCCGTTGAAT
ATGGCTCATAACACCCCTTGTATTACTGTTTATGTAAGCAGACAGTTTTATTGTTCATGACCAAAATCCCTTAACGTGAGTTTTCGTTCCACTGAGCGTCAGACCCC
GTAGAAAAGATCAAAGGATCTTCTTGAGATCCTTTTTTTCTGCGCGTAATCTGCTGCTTGCAAACAAAAAAACCACCGCTACCAGCGGTGGTTTGTTTGCCGGATCA
AGAGCTACCAACTCTTTTTCCGAAGGTAACTGGCTTCAGCAGAGCGCAGATACCAAATACTGTCCTTCTAGTGTAGCCGTAGTTAGGCCACCACTTCAAGAACTCTG
TAGCACCGCCTACATACCTCGCTCTGCTAATCCTGTTACCAGTGGCTGCTGCCAGTGGCGATAAGTCGTGTCTTACCGGGTTGGACTCAAGACGATAGTTACCGGAT
AAGGCGCAGCGGTCGGGCTGAACGGGGGGTTCGTGCACACAGCCCAGCTTGGAGCGAACGACCTACACCGAACTGAGATACCTACAGCGTGAGCTATGAGAAAGCGC
CACGCTTCCCGAAGGGAGAAAGGCGGACAGGTATCCGGTAAGCGGCAGGGTCGGAACAGGAGAGCGCACGAGGGAGCTTCCAGGGGGAAACGCCTGGTATCTTTATA
GTCCTGTCGGGTTTCGCCACCTCTGACTTGAGCGTCGATTTTTGTGATGCTCGTCAGGGGGGCGGAGCCTATGGAAAAACGCCAGCAACGCGGCCTTTTTACGGTTC
CTGGCCTTTTGCTGGCCTTTTGCTCACATGTTCTTTCCTGCGTTATCCCCTGATTCTGTGGATAACCGTATTACCGCCTTTGAGTGAGCTGATACCGCTCGCCGCAG
CCGAACGACCGAGCGCAGCGAGTCAGTGAGCGAGGAAGCGGAAGAGCGCCTGATGCGGTATTTTCTCCTTACGCATCTGTGCGGTATTTCACACCGCAATGGTGCAC
TCTCAGTACAATCTGCTCTGATGCCGCATAGTTAAGCCAGTATACACTCCGCTATCGCTACGTGACTGGGTCATGGCTGCGCCCCGACACCCGCCAACACCCGCTGA
CGCGCCCTGACGGGCTTGTCTGCTCCCGGCATCCGCTTACAGACAAGCTGTGACCGTCTCCGGGAGCTGCATGTGTCAGAGGTTTTCACCGTCATCACCGAAACGCG
CGAGGCAGCTGCGGTAAAGCTCATCAGCGTGGTCGTGAAGCGATTCACAGATGTCTGCCTGTTCATCCGCGTCCAGCTCGTTGAGTTTCTCCAGAAGCGTTAATGTC
TGGCTTCTGATAAAGCGGGCCATGTTAAGGGCGGTTTTTTCCTGTTTGGTCACTGATGCCTCCGTGTAAGGGGGATTTCTGTTCATGGGGGTAATGATACCGATGAA
ACGAGAGAGGATGCTCACGATACGGGTTACTGATGATGAACATGCCCGGTTACTGGAACGTTGTGAGGGTAAACAACTGGCGGTATGGATGCGGCGGGACCAGAGAA
AAATCACTCAGGGTCAATGCCAGCGCTTCGTTAATACAGATGTAGGTGTTCCACAGGGTAGCCAGCAGCATCCTGCGATGCAGATCCGGAACATAATGGTGCAGGGC
GCTGACTTCCGCGTTTCCAGACTTTACGAAACACGGAAACCGAAGACCATTCATGTTGTTGCTCAGGTCGCAGACGTTTTGCAGCAGCAGTCGCTTCACGTTCGCTC
GCGTATCGGTGATTCATTCTGCTAACCAGTAAGGCAACCCCGCCAGCCTAGCCGGGTCCTCAACGACAGGAGCACGATCATGCGCACCCGTGGGGCCGCCATGCCGG
CGATAATGGCCTGCTTCTCGCCGAAACGTTTGGTGGCGGGACCAGTGACGAAGGCTTGAGCGAGGGCGTGCAAGATTCCGAATACCGCAAGCGACAGGCCGATCATC
GTCGCGCTCCAGCGAAAGCGGTCCTCGCCGAAAATGACCCAGAGCGCTGCCGGCACCTGTCCTACGAGTTGCATGATAAAGAAGACAGTCATAAGTGCGGCGACGAT
AGTCATGCCCCGCGCCCACCGGAAGGAGCTGACTGGGTTGAAGGCTCTCAAGGGCATCGGTCGAGATCCCGGTGCCTAATGAGTGAGCTAACTTACATTAATTGCGT
TGCGCTCACTGCCCGCTTTCCAGTCGGGAAACCTGTCGTGCCAGCTGCATTAATGAATCGGCCAACGCGCGGGGAGAGGCGGTTTGCGTATTGGGCGCCAGGGTGGT
TTTTCTTTTCACCAGTGAGACGGGCAACAGCTGATTGCCCTTCACCGCCTGGCCCTGAGAGAGTTGCAGCAAGCGGTCCACGCTGGTTTGCCCCAGCAGGCGAAAAT
CCTGTTTGATGGTGGTTAACGGCGGGATATAACATGAGCTGTCTTCGGTATCGTCGTATCCCACTACCGAGATATCCGCACCAACGCGCAGCCCGGACTCGGTAATG
GCGCGCATTGCGCCCAGCGCCATCTGATCGTTGGCAACCAGCATCGCAGTGGGAACGATGCCCTCATTCAGCATTTGCATGGTTTGTTGAAAACCGGACATGGCACT
CCAGTCGCCTTCCCGTTCCGCTATCGGCTGAATTTGATTGCGAGTGAGATATTTATGCCAGCCAGCCAGACGCAGACGCGCCGAGACAGAACTTAATGGG
```

His Tag
Avi Tag
hPADI4

mPADI4 plasmid

TAAATCGGGCGATGCCTGTGTTGCAGAATACCAGCAGGCTGGCCGTGGTCGCCGGGGGCGGAAATGGTTTTCGCCTTTTGGCGCAAACTTATATTTGTCGATGTTCT
GGCGTCTGGAACAAGGCCCGGCGGCGGCGATTGGTTTAAGTCTGGTTATCGGTATCGTGATGGCGGAAGTATTACGCAAGCTGGGAGCAGATAAAGTTCGTGTCAAA
TGGCCTAATGACCTCTATCTGCAGGATCGCAAGCTGGCAGGCATTCTTGTGGAGCTGACTGGCAAAACTGGCGATGCGGCGCAAATAGTCATTGGAGCCGGGATCAA
CATGGCAATGCGCCGTGTTGAAGAGAGTGTCGTTAATCAGGGGTGGATCACGCTGCAGGAAGCGGGGATCAATCTCGATCGTAATACGTTGGCGGCCATGCTAATAC
GTGAATTACGTGCTGCGTTGGAACTCTTCGAACAAGAAGGATTGGCACCTTATCTGTCGCGCTGGGAAAAGCTGGATAATTTTATTAATCGCCCAGTGAAACTTATC
ATTGGTGATAAAGAAATATTTGGCATTTCACGCGGAATAGACAAACAGGGGGCTTTATTACTTGAGCAGGATGGAATAATAAAACCCTGGATGGGCGGTGAAATATC
CCTGCGTAGTGCAGAAAAATAAGCGGCCGCAGCTTAATTAGCTGAGCTTGGACTCCTGTTGATAGATCCAGTAATGACCTCAGAACTCCATCTGGATTTGTTCAGAA
CGCTCGGTTGCCGCCGGGCGTTTTTTATTGGTGAGAATCCAAGCTAGCTTGGCGAGATTTTCAGGAGCTAAGGAAGCTAAAATGGAGAAAAAAATCACTGGATATAC
CACCGTTGATATATCCCAATGGCATCGTAAAGAACATTTTGAGGCATTTCAGTCAGTTGCTCAATGTACCTATAACCAGACCGTTCAGCTGGATATTACGGCCTTTT
TAAAGACCGTAAAGAAAAATAAGCACAAGTTTTATCCGGCCTTTATTCACATTCTTGCCCGCCTGATGAATGCTCATCCGGAATTTCGTATGGCAATGAAAGACGGT
GAGCTGGTGATATGGGATAGTGTTCACCCTTGTTACACCGTTTTCCATGAGCAAACTGAAACGTTTTCATCGCTCTGGAGTGAATACCACGACGATTTCCGGCAGTT
TCTACACATATATTCGCAAGATGTGGCGTGTTACGGTGAAAACCTGGCCTATTTCCCTAAAGGGTTTATTGAGAATATGTTTTTCGTCTCAGCCAATCCCTGGGTGA
GTTTCACCAGTTTTGATTTAAACGTGGCCAATATGGACAACTTCTTCGCCCCCGTTTTCACCATGGGCAAATATTATACGCAAGGCGACAAGGTGCTGATGCCGCTG
GCGATTCAGGTTCATCATGCCGTTTGTGATGGCTTCCATGTCGGCAGAATGCTTAATGAATTACAACAGTACTGCGATGAGTGGCAGGGCGGGGCGTAATTTTTTTA
AGGCAGTTATTGGTGCCCTTAAACGCCTGGGGTAATGACTCTCTAGCTTGAGGCATCAAATAAAACGAAAGGCTCAGTCGAAAGACTGGGCCTTTCGTTTTATCTGT
TGTTTGTCGGTGAACGCTCTCCTGAGTAGGACAAATCCGCCCTCTAGATTACGTGCAGTCGATGATAAGCTGTCAAACATGAGAATTGTGCCTAATGAGTGAGCTAA
CTTACATTAATTGCGTTGCGCTCACTGCCCGCTTTCCAGTCGGGAAACCTGTCGTGCCAGCTGCATTAATGAATCGGCCAACGCGCGGGGAGAGGCGGTTTGCGTAT
TGGGCGCCAGGGTGGTTTTTCTTTTCACCAGTGAGACGGGCAACAGCTGATTGCCCTTCACCGCCTGGCCCTGAGAGAGTTGCAGCAAGCGGTCCACGCTGGTTTGC
CCCAGCAGGCGAAAATCCTGTTTGATGGTGGTTAACGGCGGGATATAACATGAGCTGTCTTCGGTATCGTCGTATCCCACTACCGAGATATCCGCACCAACGCGCAG
CCCGGACTCGGTAATGGCGCGCATTGCGCCCAGCGCCATCTGATCGTTGGCAACCAGCATCGCAGTGGGAACGATGCCCTCATTCAGCATTTGCATGGTTTGTTGAA
AACCGGACATGGCACTCCAGTCGCCTTCCCGTTCCGCTATCGGCTGAATTTGATTGCGAGTGAGATATTTATGCCAGCCAGCCAGACGCAGACGCGCCGAGACAGAA
CTTAATGGGCCCGCTAACAGCGCGATTTGCTGGTGACCCAATGCGACCAGATGCTCCACGCCCAGTCGCGTACCGTCTTCATGGGAGAAAATAATACTGTTGATGGG
TGTCTGGTCAGAGACATCAAGAAATAACGCCGGAACATTAGTGCAGGCAGCTTCCACAGCAATGGCATCCTGGTCATCCAGCGGATAGTTAATGATCAGCCCACTGA
CGCGTTGCGCGAGAAGATTGTGCACCGCCGCTTTACAGGCTTCGACGCCGCTTCGTTCTACCATCGACACCACCACGCTGGCACCCAGTTGATCGGCGCGAGATTTA
ATCGCCGCGACAATTTGCGACGGCGCGTGCAGGGCCAGACTGGAGGTGGCAACGCCAATCAGCAACGACTGTTTGCCCGCCAGTTGTTGTGCCACGCGGTTGGGAAT
GTAATTCAGCTCCGCCATCGCCGCTTCCACTTTTTCCCGCGTTTTCGCAGAAACGTGGCTGGCCTGGTTCACCACGCGGGAAACGGTCTGATAAGAGACACCGGCAT
ACTCTGCGACATCGTATAACGTTACTGGTTTCACATTCACCACCCTGAATTGACTCTCTTCCGGGCGCTATCATGCCATACCGCGAAAGGTTTTGCACCATTCGATG
GTGTCGGAATTTCGGGCAGCGTTGGGTCCTGGCCACGGGTGCGCATGATCTAGAGCTGCCTCGCGCGTTTCGGTGATGACGGTGAAAACCTCTGACACATGCAGCTC
CCGGAGACGGTCACAGCTTGTCTGTAAGCGGATGCCGGGAGCAGACAAGCCCGTCAGGGCGCGTCAGCGGGTGTTGGCGGGTGTCGGGGCGCAGCCATGACCCAGTC
ACGTAGCGATAGCGGAGTGTATACTGGCTTAACTATGCGGCATCAGAGCAGATTGTACTGAGAGTGCACCATATGCGGTGTGAAATACCGCACAGATGCGTAAGGAG
AAAATACCGCATCAGGCGCTCTTCCGCTTCCTCGCTCACTGACTCGCTGCGCTCGGTCGTTCGGCTGCGGCGAGCGGTATCAGCTCACTCAAAGGCGGTAATACGGT
TATCCACAGAATCAGGGGATAACGCAGGAAAGAACATGTGAGCAAAAGGCCAGCAAAAGGCCAGGAACCGTAAAAAGGCCGCGTTGCTGGCGTTTTTCCATAGGCTC
CGCCCCCCTGACGAGCATCACAAAAATCGACGCTCAAGTCAGAGGTGGCGAAACCCGACAGGACTATAAAGATACCAGGCGTTTCCCCCTGGAAGCTCCCTCGTGCG
CTCTCCTGTTCCGACCCTGCCGCTTACCGGATACCTGTCCGCCTTTCTCCCTTCGGGAAGCGTGGCGCTTTCTCATAGCTCACGCTGTAGGTATCTCAGTTCGGTGT
AGGTCGTTCGCTCCAAGCTGGGCTGTGTGCACGAACCCCCCGTTCAGCCCGACCGCTGCGCCTTATCCGGTAACTATCGTCTTGAGTCCAACCCGGTAAGACACGAC
TTATCGCCACTGGCAGCAGCCACTGGTAACAGGATTAGCAGAGCGAGGTATGTAGGCGGTGCTACAGAGTTCTTGAAGTGGTGGCCTAACTACGGCTACACTAGAAG
GACAGTATTTGGTATCTGCGCTCTGCTGAAGCCAGTTACCTTCGGAAAAAGAGTTGGTAGCTCTTGATCCGGCAAACAAACCACCGCTGGTAGCGGTGGTTTTTTTG
TTTGCAAGCAGCAGATTACGCGCAGAAAAAAAGGATCTCAAGAAGATCCTTTGATCTTTTCTACGGGGTCTGACGCTCAGTGGAACGAAAACTCACGTTAAGGGATT
TTGGTCATGAGATTATCAAAAAGGATCTTCACCTAGATCCTTTTAAATTAAAAATGAAGTTTTAAATCAATCTAAAGTATATATGAGTAAACTTGGTCTGACAGTTA
CCAATGCTTAATCAGTGAGGCACCTATCTCAGCGATCTGTCTATTTCGTTCATCCATAGTTGCCTGACTCCCCGTCGTGTAGATAACTACGATACGGGAGGGCTTAC
CATCTGGCCCCAGTGCTGCAATGATACCGCGAGACCCACGCTCACCGGCTCCAGATTTATCAGCAATAAACCAGCCAGCCGGAAGGGCCGAGCGCAGAAGTGGTCCT
GCAACTTTATCCGCCTCCATCCAGTCTATTAATTGTTGCCGGGAAGCTAGAGTAAGTAGTTCGCCAGTTAATAGTTTGCGCAACGTTGTTGCCATTGCTACAGGCAT
CGTGGTGTCACGCTCGTCGTTTGGTATGGCTTCATTCAGCTCCGGTTCCCAACGATCAAGGCGAGTTACATGATCCCCCATGTTGTGCAAAAAAGCGGTTAGCTCCT
TCGGTCCTCCGATCGTTGTCAGAAGTAAGTTGGCCGCAGTGTTATCACTCATGGTTATGGCAGCACTGCATAATTCTCTTACTGTCATGCCATCCGTAAGATGCTTT
TCTGTGACTGGTGAGTACTCAACCAAGTCATTCTGAGAATAGTGTATGCGGCGACCGAGTTGCTCTTGCCCGGCGTCAATACGGGATAATACCGCGCCACATAGCAG
AACTTTAAAAGTGCTCATCATTGGAAAACGTTCTTCGGGGCGAAAACTCTCAAGGATCTTACCGCTGTTGAGATCCAGTTCGATGTAACCCACTCGTGCACCCAACT
GATCTTCAGCATCTTTTACTTTCACCAGCGTTTCTGGGTGAGCAAAAACAGGAAGGCAAAATGCCGCAAAAAAGGGAATAAGGGCGACACGGAAATGTTGAATACTC
ATACTCTTCCTTTTTCAATATTATTGAAGCATTTATCAGGGTTATTGTCTCATGAGCGGATACATATTTGAATGTATTTAGAAAAATAAACAAATAGGGGTTCCGCG
CACATTTCCCCGAAAAGTGCCACCTGACGTCTAAGAAACCATTATTATCATGACATTAACCTATAAAAATAGGCGTATCACGAGGCCCTTTCGTCTTCACCTCGATT
CGTCTTCACCTCGAGAAATCATAAAAAATTTATTTGCTTTGTGAGCGGATAACAATTATAATAGATTCAATTGTGAGCGGATAACAATTTCACACAGAATTTATTAA
AGAGGAGAAATTACATATGTCCGGCCTGAACGACATCTTCGAGGCTCAGAAAATCGAATGGCACGAAGGTCACCATCATCACCACCACGGATCCATGGCCCAGGGTG
CGGTGATCCACGTGGCCCCCGAGCAGCCCACTCACGCCGTGTGTGTGGTGGGCACAGCGACCCCGCTGGATGTCCGCGGTTCTGCTCCTAAGGGCTACACAACCTTC
GGCATCACAGCCTCTCCAGGAGTCATCGTAGATGTCATCCATGGTCCTCCAGTCAAGAAGAGTACCATGGGGGCCTCCAAATGGCCTCTGGACCCTGAGCTGGAGGT
GACCCTACAGGTGAAAGCAGCCAGCAGCAGAACAGATGATGAAAAGGTTCGAGTTTCATACTATGGACCCAAGACCTCCCCAGTCCAAGCCCTGATCTACATCACTG
GGGTGGAACTGTCCCTGAGCGCAGATGTCACCCGCACAGGCAGAGTGAAGCCAGCCCAAGCCGGGAAGGATCAGAGCACCTGGACCTGGGGCCCGGGCGGCCGTGGC
GCCATCCTGTTGGTGAACTGTGACAAAGAGGACCCTCAGGCCTCCGGAATGGACTTTGAGGATGACAAGATCTTGGACAACAAAGACCTGCAGGACATGTCTCCAAT
GACCCTAAGCACGAAGACGCCCAAAGACTTCTTTGAAAAGTATCAGCTGGTGTTGGAGGTGCCCAAGGCCAAGATGAACAGAGTGAGAGTCTTCCGGGCCACACGGG
GCAAACTGCCGTCCCGGTACAAGGTGGCCCTGGGACCACAACAGTTCTCGTATTGCCTGGAGCTGCCCGGCGGCCAGCACAGCACAGACTTCTATGTGGAAGGCCTT
GCTTTCCCAGACGCAGACTTCAAAGGGCTCATTCCCCTCACCATCTCCCTGCTGGACAAGTCTAACCCGGAGCTCCCCGAGGCCCTGGTGTTCCAAGACAGTGTGAC
GTTCCGTGTGGCCCCCTGGATCATGACCCCCAACACTCAGCCCCCCCAGGAGGTGTACGTGTGCAGGGTTTCTGACAATGAAGACTTCCTAAAGTCACTAGCTACTC
TGACCAAGAAAGCCAAGTGCAAGCTGACTGTGTGCCCCGAGGAGGAGAATATAGATGACCAATGGATGCAGGACGAAATGGAGATTGGCTACATCCAGGCCCCACAC
AAGACGCTGCCTGTGGTCTTTGACTCCCCGAGGGACAGAGGCCTGAAGGATTTCCCTGTCAAGCGAGTTATGGGTCCAAATTTTGGCTATGTGACCCGAAAGCTCTA
TATGTCAGAGCTCACTGGGCTGGATGCCTTTGGGAACCTGGAGGTGAGTCCCCCAGTCACTGTCAGAGGGAAGGAGTACCCACTGGGCAGAATTCTCATCGGGAATA
GCGGTTACTCCAGCAGCGAGAGCCGGGACATGCACCAGGCCCTGCAGGACTTCCTGAGCGCCCAGCAGGTGCAGGCCCCCGTGAGGCTCTTCTCCGATTGGCTCTTT
GTGGGTCACGTGGATGAGTTCTTGAGCTTTGTCCCAGCGCGGGACAAGCAGGGTTTCGGCTGCTGCTGTCCAGCCCCAGAGCTTGCTATCAGCTGTTCCAGGAGCT
ACAGAGCCAGGGCCACGGGGAGGCGACACTGTTCGAAGGACTCAAGAGGAAAAGGCAGACAATCAATGAAATTCTGTCCAACAAGAAATTAAGAGACCAGAATGCCT
ATGTGGAGAGCTGTATCGACTGGAACCGGGCGGTGCTGAAGCGGGAGCTGGGCCTGGCAGAGGGTGACATCATCGACATTCCGCAGCTCTTCAAGCTCGCGGGGAAC
TCCAGAGGGAACTCTAAGGCCCAGGCCTTCTTCCCAAACATGGTGAACATGCTGGTCCTGGGCAAGTACCTGGGCATCCCCAAGCCCTTTGGGCCCATCATCGATGG
CCACTGCTGCCTGGAGGAGGAGGTGCGTTCCCACCTGGAGCCGCTGGGTCTGCACTGCACCTTCATCAACGACTTCTACACCTACCACGTGTACAACGGGGAGGTTC
ACTGTGGCACCAATGTGCGCAGGAAGCCCTTCACCTTCAAGTGGTGGCACATGGTGCCCTGAGAATTCAGGGTACCCAATAAGCTTACAATTGGTGGTACATAGAAT
GGTTAACTTTAACAAGGAGAAAAACATGAAGGATAACACCGTGCCACTGAAATTGATTGCCCTGTTAGCGAACGGTGAATTTCACTCTGGCGAGCAGTTGGGTGAAA
CGCTGGGAATGAGCCGGGCGGCTATTAATAAACACATTCAGACACTGCGTGACTGGGGCGTTGATGTCTTTACCGTTCCGGGTAAAGGATACAGCCTGCCTGAGCCC
ATCCAGTTACTTAATGCTGAACAGATATTGGGTCAGCTGGATGGCGGTAGTGTAGCCGTGCTGCCAGTTATTGACTCCACGAATCAGTACCTTCTTGATCGTATCGG
AGAGCT

His Tag
Avi Tag
mPADI4

hPADI4_D165A plasmid

GCTAACAGCGCGATTTGCTGGTGACCCAATGCGACCAGATGCTCCACGCCCAGTCGCGTACCGTCTTCATGGGAGAAAATAATACTGTTGATGGGTGTCTGGTCAGA
GACATCAAGAAATAACGCCGGAACATTAGTGCAGGCAGCTTCCACAGCAATGGCATCCTGGTCATCCAGCGGATAGTTAATGATCAGCCCACTGACGCGTTGCGCGA
GAAGATTGTGCACCGCCGCTTTACAGGCTTCGACGCCGCTTCGTTCTACCATCGACACCACCACGCTGGCACCCAGTTGATCGGCGCGAGATTTAATCGCCGCGACA
ATTTGCGACGGCGCGTGCAGGGCCAGACTGGAGGTGGCAACGCCAATCAGCAACGACTGTTTGCCCGCCAGTTGTTGTGCCACGCGGTTGGGAATGTAATTCAGCTC
CGCCATCGCCGCTTCCACTTTTTCCCGCGTTTTCGCAGAAACGTGGCTGGCCTGGTTCACCACGCGGGAAACGGTCTGATAAGAGACACCGGCATACTCTGCGACAT
CGTATAACGTTACTGGTTTCACATTCACCACCCTGAATTGACTCTCTTCCGGGCGCTATCATGCCATACCGCGAAAGGTTTTGCGCCATTCGATGGTGTCCGGGATC
TCGACGCTCTCCCTTATGCGACTCCTGCATTAGGAAGCAGCCCAGTAGTAGGTTGAGGCCGTTGAGCACCGCCGCCGCAAGGAATGGTGCATGCAAGGAGATGGCGC
CCAACAGTCCCCCGGCCACGGGGCCTGCCACCATACCCACGCCGAAACAAGCGCTCATGAGCCCGAAGTGGCGAGCCCGATCTTCCCCATCGGTGATGTCGGCGATA
TAGGCGCCAGCAACCGCACCTGTGGCGCCGGTGATGCCGGCCACGATGCGTCCGGCGTAGAGGATCGAGATCTCGATCCCGCGAAATTAATACGACTCACTATAGGG
GAATTGTGAGCGGATAACAATTCCCCTCTAGAAATAATTTTGTTTAACTTTAAGAAGGAGATATACCATGAGCGGCCTGAACGATATTTTTGAAGCGCAGAAAATTG
AATGGCATGAAGGCAGCGCTGGAGGTTCAGGTCATCACCACCATCATCACGGTAGTGGTGGAATGGCCCAGGGGACATTGATCCGTGTGACCCCAGAGCAGCCCACC
CATGCCGTGTGTGTGCTGGGCACCTTGACTCAGCTTGACATCTGCAGCTCTGCCCCTGAGGACTGCACGTCCTTCAGCATCAACGCCTCCCCAGGGGTGGTCGTGGA
TATTGCCCACAGCCCTCCAGCCAAGAAGAAATCCACAGGTTCCTCCACATGGCCCCTGGACCCTGGGGTAGAGGTGACCCTGACGATGAAAGCGGCCAGTGGTAGCA
CAGGCGACCAGAAGGTTCAGATTTCATACTACGGACCCAAGACTCCACCAGTCAAAGCTCTACTCTACCTCACCGCGGTGGAAATCTCCCTGTGCGCAGACATCACC
CGCACCGGCAAAGTGAAGCCAACCAGAGCTGTGAAAGATCAGAGGACCTGGACCTGGGGCCCTTGTGGACAGGGTGCCATCCTGCTGGTGAACTGTGACAGAGACAA
TCTCGAATCTTCTGCCATGGCCTGCGAGGATGATGAAGTGCTTGACAGCGAAGACCTGCAGGACATGTCGCTGATGACCCTGAGCACGAAGACCCCCAAGGACTTCT
TCACAAACCATACACTGGTGCTCCACGTGGCCAGGTCTGAGATGGACAAAGTGAGGGTGTTTCAGGCCACACGGGGCAAACTGTCCTCCAAGTGCAGCGTAGTCTTG
GGTCCCAAGTGGCCCTCTCACTACCTGATGGTCCCCGGTGGAAAGCACAACATGGACTTCTACGTGGAGGCCCTCGCTTTCCCGGACACCGACTTCCCGGGGCTCAT
TACCCTCACCATCTCCCTGCTGGACACGTCCAACCTGGAGCTCCCCGAGGCTGTGGTGTTCCAAGACAGCGTGGTCTTCCGCGTGGCGCCCTGGATCATGACCCCCA
ACACCCAGCCCCCGCAGGAGGTGTACGCGTGCAGTATTTTTGAAAATGAGGACTTCCTGAAGTCAGTGACTACTCTGGCCATGAAAGCCAAGTGCAAGCTGACCATC
TGCCCTGAGGAGGAGAACATGGATGACCAGTGGATGCAGGATGAAATGGAGATcGGCTACATCCAAGCCCCACACAAAACGCTGCCCGTGGTCTTCGACTCTCCAAG
GAACAGAGGCCTGAAGGAGTTTCCCATCAAACGAGTGATGGGTCCAGATTTTGGCTATGTAACTCGAGGGCCCCAAACAGGGGGTATCAGTGGACTGGACTCCTTTG
GGAACCTGGAAGTGAGCCCCCCAGTCACAGTCAGGGGCAAGGAATACCCGCTGGGCAGGATTCTCTTCGGGGACAGCTGTTATCCCAGCAATGACAGCCGGCAGATG
CACCAGGCCCTACAGGACTTCCTCAGTGCCCAGCAGGTGCAGGCCCCTGTGAAGCTCTATTCTGACTGGCTGTCCGTGGGCCACGTGGACGAGTTCCTGAGCTTTGT
GCCAGCACCCGACAGGAAGGGCTTCCGGCTGCTCCTGGCCAGCCCCAGGTCCTGCTACAAACTGTTCCAGGAGCAGCAGAATGAGGGCCACGGGGAGGCCCTGCTGT
TCGAAGGGATCAAGAAAAAAAAACAGCAGAAAATAAAGAACATTCTGTCAAACAAGACATTGAGAGAACATAATTCATTTGTGGAGAGATGCATCGACTGGAACCGC
GAGCTGCTGAAGCGGGAGCTGGGCCTGGCCGAGAGTGACATCATTGACATCCCGCAGCTCTTCAAGCTCAAAGAGTTCTCTAAGGCGGAAGCTTTTTTCCCCAACAT
GGTGAACATGCTGGTGCTAGGGAAGCACCTGGGCATCCCCAAGCCCTTCGGGCCCGTCATCAACGGCCGCTGCTGCCTGGAGGAGAAGGTGTGTTCCCTGCTGGAGC
CACTGGGCCTCCAGTGCACCTTCATCAACGACTTCTTCACCTACCACATCAGGCATGGGGAGGTGCACTGCGGCACCAACGTGCGCAGAAAGCCCTTCTCCTTCAAG
TGGTGGAACATGGTGCCCTGAGGTGGAAGTGGTCATCACCACCATCATCACTGATGACGAAGCTTGCGGCCGCACTCGAGCACCACCACCACCACCACTGAGATCCG
GCTGCTAACAAAGCCCGAAAGGAAGCTGAGTTGGCTGCTGCCACCGCTGAGCAATAACTAGCATAACCCCTTGGGGCCTCTAAACGGGTCTTGAGGGGTTTTTTGCT
GAAAGGAGGAACTATATCCGGATTGGCGAATGGGACGCGCCCTGTAGCGGCGCATTAAGCGCGGCGGGTGTGGTGGTTACGCGCAGCGTGACCGCTACACTTGCCAG
CGCCCTAGCGCCCGCTCCTTTCGCTTTCTTCCCTTCCTTTCTCGCCACGTTCGCCGGCTTTCCCCGTCAAGCTCTAAATCGGGGGCTCCCTTTAGGGTTCCGATTTA
GTGCTTTACGGCACCTCGACCCCAAAAAACTTGATTAGGGTGATGGTTCACGTAGTGGGCCATCGCCCTGATAGACGGTTTTTCGCCCTTTGACGTTGGAGTCCACG
TTCTTTAATAGTGGACTCTTGTTCCAAACTGGAACAACACTCAACCCTATCTCGGTCTATTCTTTTGATTTATAAGGGATTTTGCCGATTTCGGCCTATTGGTTAAA
AAATGAGCTGATTTAACAAAAATTTAACGCGAATTTTAACAAAATATTAACGCTTACAATTTAGGTGGCACTTTTCGGGGAAATGTGCGCGGAACCCCTATTTGTTT
ATTTTTCTAAATACATTCAAATATGTATCCGCTCATGAATTAATTCTTAGAAAAACTCATCGAGCATCAAATGAAACTGCAATTTATTCATATCAGGATTATCAATA
CCATATTTTTGAAAAAGCCGTTTCTGTAATGAAGGAGAAAACTCACCGAGGCAGTTCCATAGGATGGCAAGATCCTGGTATCGGTCTGCGATTCCGACTCGTCCAAC
ATCAATACAACCTATTAATTTCCCCTCGTCAAAAATAAGGTTATCAAGTGAGAAATCACCATGAGTGACGACTGAATCCGGTGAGAATGGCAAAAGTTTATGCATTT
CTTTCCAGACTTGTTCAACAGGCCAGCCATTACGCTCGTCATCAAAATCACTCGCATCAACCAAACCGTTATTCATTCGTGATTGCGCCTGAGCGAGACGAAATACG
CGATCGCTGTTAAAAGGACAATTACAAACAGGAATCGAATGCAACCGGCGCAGGAACACTGCCAGCGCATCAACAATATTTTCACCTGAATCAGGATATTCTTCTAA
TACCTGGAATGCTGTTTTCCCGGGGATCGCAGTGGTGAGTAACCATGCATCATCAGGAGTACGGATAAAATGCTTGATGGTCGGAAGAGGCATAAATTCCGTCAGCC
AGTTTAGTCTGACCATCTCATCTGTAACATCATTGGCAACGCTACCTTTGCCATGTTTCAGAAACAACTCTGGCGCATCGGGCTTCCCATACAATCGATAGATTGTC
GCACCTGATTGCCCGACATTATCGCGAGCCCATTTATACCCATATAAATCAGCATCCATGTTGGAATTTAATCGCGGCCTAGAGCAAGACGTTTCCCGTTGAATATG
GCTCATAACACCCCTTGTATTACTGTTTATGTAAGCAGACAGTTTTATTGTTCATGACCAAAATCCCTTAACGTGAGTTTTCGTTCCACTGAGCGTCAGACCCCGTA
GAAAAGATCAAAGGATCTTCTTGAGATCCTTTTTTTCTGCGCGTAATCTGCTGCTTGCAAACAAAAAAACCACCGCTACCAGCGGTGGTTTGTTTGCCGGATCAAGA
GCTACCAACTCTTTTTCCGAAGGTAACTGGCTTCAGCAGAGCGCAGATACCAAATACTGTCCTTCTAGTGTAGCCGTAGTTAGGCCACCACTTCAAGAACTCTGTAG
CACCGCCTACATACCTCGCTCTGCTAATCCTGTTACCAGTGGCTGCTGCCAGTGGCGATAAGTCGTGTCTTACCGGGTTGGACTCAAGACGATAGTTACCGGATAAG
GCGCAGCGGTCGGGCTGAACGGGGGGTTCGTGCACACAGCCCAGCTTGGAGCGAACGACCTACACCGAACTGAGATACCTACAGCGTGAGCTATGAGAAAGCGCCAC
GCTTCCCGAAGGGAGAAAGGCGGACAGGTATCCGGTAAGCGGCAGGGTCGGAACAGGAGAGCGCACGAGGGAGCTTCCAGGGGGAAACGCCTGGTATCTTTATAGTC
CTGTCGGGTTTCGCCACCTCTGACTTGAGCGTCGATTTTTGTGATGCTCGTCAGGGGGGCGGAGCCTATGGAAAAACGCCAGCAACGCGGCCTTTTTACGGTTCCTG
GCCTTTTGCTGGCCTTTTGCTCACATGTTCTTTCCTGCGTTATCCCCTGATTCTGTGGATAACCGTATTACCGCCTTTGAGTGAGCTGATACCGCTCGCCGCAGCCG
AACGACCGAGCGCAGCGAGTCAGTGAGCGAGGAAGCGGAAGAGCGCCTGATGCGGTATTTTCTCCTTACGCATCTGTGCGGTATTTCACACCGCAATGGTGCACTCT
CAGTACAATCTGCTCTGATGCCGCATAGTTAAGCCAGTATACACTCCGCTATCGCTACGTGACTGGGTCATGGCTGCGCCCCGACACCCGCCAACACCCGCTGACGC
GCCCTGACGGGCTTGTCTGCTCCCGGCATCCGCTTACAGACAAGCTGTGACCGTCTCCGGGAGCTGCATGTGTCAGAGGTTTTCACCGTCATCACCGAAACGCGCGA
GGCAGCTGCGGTAAAGCTCATCAGCGTGGTCGTGAAGCGATTCACAGATGTCTGCCTGTTCATCCGCGTCCAGCTCGTTGAGTTTCTCCAGAAGCGTTAATGTCTGG
CTTCTGATAAAGCGGGCCATGTTAAGGGCGGTTTTTTCCTGTTTGGTCACTGATGCCTCCGTGTAAGGGGGATTTCTGTTCATGGGGGTAATGATACCGATGAAACG
AGAGAGGATGCTCACGATACGGGTTACTGATGATGAACATGCCCGGTTACTGGAACGTTGTGAGGGTAAACAACTGGCGGTATGGATGCGGCGGGACCAGAGAAAAA
TCACTCAGGGTCAATGCCAGCGCTTCGTTAATACAGATGTAGGTGTTCCACAGGGTAGCCAGCAGCATCCTGCGATGCAGATCCGGAACATAATGGTGCAGGGCGCT
GACTTCCGCGTTTCCAGACTTTACGAAACACGGAAACCGAAGACCATTCATGTTGTTGCTCAGGTCGCAGACGTTTTGCAGCAGCAGTCGCTTCACGTTCGCTCGCG
TATCGGTGATTCATTCTGCTAACCAGTAAGGCAACCCCGCCAGCCTAGCCGGGTCCTCAACGACAGGAGCACGATCATGCGCACCCGTGGGGCCGCCATGCCGGCGA
TAATGGCCTGCTTCTCGCCGAAACGTTTGGTGGCGGGACCAGTGACGAAGGCTTGAGCGAGGGCGTGCAAGATTCCGAATACCGCAAGCGACAGGCCGATCATCGTC
GCGCTCCAGCGAAAGCGGTCCTCGCCGAAAATGACCCAGAGCGCTGCCGGCACCTGTCCTACGAGTTGCATGATAAAGAAGACAGTCATAAGTGCGGCGACGATAGT
CATGCCCCGCGCCCACCGGAAGGAGCTGACTGGGTTGAAGGCTCTCAAGGGCATCGGTCGAGATCCCGGTGCCTAATGAGTGAGCTAACTTACATTAATTGCGTTGC
GCTCACTGCCCGCTTTCCAGTCGGGAAACCTGTCGTGCCAGCTGCATTAATGAATCGGCCAACGCGCGGGGAGAGGCGGTTTGCGTATTGGGCGCCAGGGTGGTTTT
TCTTTTCACCAGTGAGACGGGCAACAGCTGATTGCCCTTCACCGCCTGGCCCTGAGAGAGTTGCAGCAAGCGGTCCACGCTGGTTTGCCCCAGCAGGCGAAAATCCT
GTTTGATGGTGGTTAACGGCGGGATATAACATGAGCTGTCTTCGGTATCGTCGTATCCCACTACCGAGATATCCGCACCAACGCGCAGCCCGGACTCGGTAATGGCG
CGCATTGCGCCCAGCGCCATCTGATCGTTGGCAACCAGCATCGCAGTGGGAACGATGCCCTCATTCAGCATTTGCATGGTTTGTTGAAAACCGGACATGGCACTCCA
GTCGCCTTCCCGTTCCGCTATCGGCTGAATTTGATTGCGAGTGAGATATTTATGCCAGCCAGCCAGACGCAGACGCGCCGAGACAGAACTTAATGGGCCC

His Tag
Avi Tag
hPADI4
D165A variant

hPADI4_C166F plasmid

GCTAACAGCGCGATTTGCTGGTGACCCAATGCGACCAGATGCTCCACGCCCAGTCGCGTACCGTCTTCATGGGAGAAAATAATACTGTTGATGGGTGTCTGGTCAGA
GACATCAAGAAATAACGCCGGAACATTAGTGCAGGCAGCTTCCACAGCAATGGCATCCTGGTCATCCAGCGGATAGTTAATGATCAGCCCACTGACGCGTTGCGCGA
GAAGATTGTGCACCGCCGCTTTACAGGCTTCGACGCCGCTTCGTTCTACCATCGACACCACCACGCTGGCACCCAGTTGATCGGCGCGAGATTTAATCGCCGCGACA
ATTTGCGACGGCGCGTGCAGGGCCAGACTGGAGGTGGCAACGCCAATCAGCAACGACTGTTTGCCCGCCAGTTGTTGTGCCACGCGGTTGGGAATGTAATTCAGCTC
CGCCATCGCCGCTTCCACTTTTTCCCGCGTTTTCGCAGAAACGTGGCTGGCCTGGTTCACCACGCGGGAAACGGTCTGATAAGAGACACCGGCATACTCTGCGACAT
CGTATAACGTTACTGGTTTCACATTCACCACCCTGAATTGACTCTCTTCCGGGCGCTATCATGCCATACCGCGAAAGGTTTTGCGCCATTCGATGGTGTCCGGGATC
TCGACGCTCTCCCTTATGCGACTCCTGCATTAGGAAGCAGCCCAGTAGTAGGTTGAGGCCGTTGAGCACCGCCGCCGCAAGGAATGGTGCATGCAAGGAGATGGCGC
CCAACAGTCCCCCGGCCACGGGGCCTGCCACCATACCCACGCCGAAACAAGCGCTCATGAGCCCGAAGTGGCGAGCCCGATCTTCCCCATCGGTGATGTCGGCGATA
TAGGCGCCAGCAACCGCACCTGTGGCGCCGGTGATGCCGGCCACGATGCGTCCGGCGTAGAGGATCGAGATCTCGATCCCGCGAAATTAATACGACTCACTATAGGG
GAATTGTGAGCGGATAACAATTCCCCTCTAGAAATAATTTTGTTTAACTTTAAGAAGGAGATATACCATGAGCGGCCTGAACGATATTTTTGAAGCGCAGAAAATTG
AATGGCATGAAGGCAGCGCTGGAGGTTCAGGTCATCACCACCATCATCACGGTAGTGGTGGAATGGCCCAGGGGACATTGATCCGTGTGACCCCAGAGCAGCCCACC
CATGCCGTGTGTGTGCTGGGCACCTTGACTCAGCTTGACATCTGCAGCTCTGCCCCTGAGGACTGCACGTCCTTCAGCATCAACGCCTCCCCAGGGGTGGTCGTGGA
TATTGCCCACAGCCCTCCAGCCAAGAAGAAATCCACAGGTTCCTCCACATGGCCCCTGGACCCTGGGGTAGAGGTGACCCTGACGATGAAAGCGGCCAGTGGTAGCA
CAGGCGACCAGAAGGTTCAGATTTCATACTACGGACCCAAGACTCCACCAGTCAAAGCTCTACTCTACCTCACCGCGGTGGAAATCTCCCTGTGCGCAGACATCACC
CGCACCGGCAAAGTGAAGCCAACCAGAGCTGTGAAAGATCAGAGGACCTGGACCTGGGGCCCTTGTGGACAGGGTGCCATCCTGCTGGTGAACTGTGACAGAGACAA
TCTCGAATCTTCTGCCATGGACTTCGAGGATGATGAAGTGCTTGACAGCGAAGACCTGCAGGACATGTCGCTGATGACCCTGAGCACGAAGACCCCCAAGGACTTCT
TCACAAACCATACACTGGTGCTCCACGTGGCCAGGTCTGAGATGGACAAAGTGAGGGTGTTTCAGGCCACACGGGGCAAACTGTCCTCCAAGTGCAGCGTAGTCTTG
GGTCCCAAGTGGCCCTCTCACTACCTGATGGTCCCCGGTGGAAAGCACAACATGGACTTCTACGTGGAGGCCCTCGCTTTCCCGGACACCGACTTCCCGGGGCTCAT
TACCCTCACCATCTCCCTGCTGGACACGTCCAACCTGGAGCTCCCCGAGGCTGTGGTGTTCCAAGACAGCGTGGTCTTCCGCGTGGCGCCCTGGATCATGACCCCCA
ACACCCAGCCCCCGCAGGAGGTGTACGCGTGCAGTATTTTTGAAAATGAGGACTTCCTGAAGTCAGTGACTACTCTGGCCATGAAAGCCAAGTGCAAGCTGACCATC
TGCCCTGAGGAGGAGAACATGGATGACCAGTGGATGCAGGATGAAATGGAGATtGGCTACATCCAAGCCCCACACAAAACGCTGCCCGTGGTCTTCGACTCTCCAAG
GAACAGAGGCCTGAAGGAGTTTCCCATCAAACGAGTGATGGGTCCAGATTTTGGCTATGTAACTCGAGGGCCCCAAACAGGGGGTATCAGTGGACTGGACTCCTTTG
GGAACCTGGAAGTGAGCCCCCCAGTCACAGTCAGGGGCAAGGAATACCCGCTGGGCAGGATTCTCTTCGGGGACAGCTGTTATCCCAGCAATGACAGCCGGCAGATG
CACCAGGCCCTACAGGACTTCCTCAGTGCCCAGCAGGTGCAGGCCCCTGTGAAGCTCTATTCTGACTGGCTGTCCGTGGGCCACGTGGACGAGTTCCTGAGCTTTGT
GCCAGCACCCGACAGGAAGGGCTTCCGGCTGCTCCTGGCCAGCCCCAGGTCCTGCTACAAACTGTTCCAGGAGCAGCAGAATGAGGGCCACGGGGAGGCCCTGCTGT
TCGAAGGGATCAAGAAAAAAAAACAGCAGAAAATAAAGAACATTCTGTCAAACAAGACATTGAGAGAACATAATTCATTTGTGGAGAGATGCATCGACTGGAACCGC
GAGCTGCTGAAGCGGGAGCTGGGCCTGGCCGAGAGTGACATCATTGACATCCCGCAGCTCTTCAAGCTCAAAGAGTTCTCTAAGGCGGAAGCTTTTTTCCCCAACAT
GGTGAACATGCTGGTGCTAGGGAAGCACCTGGGCATCCCCAAGCCCTTCGGGCCCGTCATCAACGGCCGCTGCTGCCTGGAGGAGAAGGTGTGTTCCCTGCTGGAGC
CACTGGGCCTCCAGTGCACCTTCATCAACGACTTCTTCACCTACCACATCAGGCATGGGGAGGTGCACTGCGGCACCAACGTGCGCAGAAAGCCCTTCTCCTTCAAG
TGGTGGAACATGGTGCCCTGAGGTGGAAGTGGTCATCACCACCATCATCACTGATGACGAAGCTTGCGGCCGCACTCGAGCACCACCACCACCACCACTGAGATCCG
GCTGCTAACAAAGCCCGAAAGGAAGCTGAGTTGGCTGCTGCCACCGCTGAGCAATAACTAGCATAACCCCTTGGGGCCTCTAAACGGGTCTTGAGGGGTTTTTTGCT
GAAAGGAGGAACTATATCCGGATTGGCGAATGGGACGCGCCCTGTAGCGGCGCATTAAGCGCGGCGGGTGTGGTGGTTACGCGCAGCGTGACCGCTACACTTGCCAG
CGCCCTAGCGCCCGCTCCTTTCGCTTTCTTCCCTTCCTTTCTCGCCACGTTCGCCGGCTTTCCCCGTCAAGCTCTAAATCGGGGGCTCCCTTTAGGGTTCCGATTTA
GTGCTTTACGGCACCTCGACCCCAAAAAACTTGATTAGGGTGATGGTTCACGTAGTGGGCCATCGCCCTGATAGACGGTTTTTCGCCCTTTGACGTTGGAGTCCACG
TTCTTTAATAGTGGACTCTTGTTCCAAACTGGAACAACACTCAACCCTATCTCGGTCTATTCTTTTGATTTATAAGGGATTTTGCCGATTTCGGCCTATTGGTTAAA
AAATGAGCTGATTTAACAAAAATTTAACGCGAATTTTAACAAAATATTAACGCTTACAATTTAGGTGGCACTTTTCGGGGAAATGTGCGCGGAACCCCTATTTGTTT
ATTTTTCTAAATACATTCAAATATGTATCCGCTCATGAATTAATTCTTAGAAAAACTCATCGAGCATCAAATGAAACTGCAATTTATTCATATCAGGATTATCAATA
CCATATTTTTGAAAAAGCCGTTTCTGTAATGAAGGAGAAAACTCACCGAGGCAGTTCCATAGGATGGCAAGATCCTGGTATCGGTCTGCGATTCCGACTCGTCCAAC
ATCAATACAACCTATTAATTTCCCCTCGTCAAAAATAAGGTTATCAAGTGAGAAATCACCATGAGTGACGACTGAATCCGGTGAGAATGGCAAAAGTTTATGCATTT
CTTTCCAGACTTGTTCAACAGGCCAGCCATTACGCTCGTCATCAAAATCACTCGCATCAACCAAACCGTTATTCATTCGTGATTGCGCCTGAGCGAGACGAAATACG
CGATCGCTGTTAAAAGGACAATTACAAACAGGAATCGAATGCAACCGGCGCAGGAACACTGCCAGCGCATCAACAATATTTTCACCTGAATCAGGATATTCTTCTAA
TACCTGGAATGCTGTTTTCCCGGGGATCGCAGTGGTGAGTAACCATGCATCATCAGGAGTACGGATAAAATGCTTGATGGTCGGAAGAGGCATAAATTCCGTCAGCC
AGTTTAGTCTGACCATCTCATCTGTAACATCATTGGCAACGCTACCTTTGCCATGTTTCAGAAACAACTCTGGCGCATCGGGCTTCCCATACAATCGATAGATTGTC
GCACCTGATTGCCCGACATTATCGCGAGCCCATTTATACCCATATAAATCAGCATCCATGTTGGAATTTAATCGCGGCCTAGAGCAAGACGTTTCCCGTTGAATATG
GCTCATAACACCCCTTGTATTACTGTTTATGTAAGCAGACAGTTTTATTGTTCATGACCAAAATCCCTTAACGTGAGTTTTCGTTCCACTGAGCGTCAGACCCCGTA
GAAAAGATCAAAGGATCTTCTTGAGATCCTTTTTTTCTGCGCGTAATCTGCTGCTTGCAAACAAAAAAACCACCGCTACCAGCGGTGGTTTGTTTGCCGGATCAAGA
GCTACCAACTCTTTTTCCGAAGGTAACTGGCTTCAGCAGAGCGCAGATACCAAATACTGTCCTTCTAGTGTAGCCGTAGTTAGGCCACCACTTCAAGAACTCTGTAG
CACCGCCTACATACCTCGCTCTGCTAATCCTGTTACCAGTGGCTGCTGCCAGTGGCGATAAGTCGTGTCTTACCGGGTTGGACTCAAGACGATAGTTACCGGATAAG
GCGCAGCGGTCGGGCTGAACGGGGGGTTCGTGCACACAGCCCAGCTTGGAGCGAACGACCTACACCGAACTGAGATACCTACAGCGTGAGCTATGAGAAAGCGCCAC
GCTTCCCGAAGGGAGAAAGGCGGACAGGTATCCGGTAAGCGGCAGGGTCGGAACAGGAGAGCGCACGAGGGAGCTTCCAGGGGGAAACGCCTGGTATCTTTATAGTC
CTGTCGGGTTTCGCCACCTCTGACTTGAGCGTCGATTTTTGTGATGCTCGTCAGGGGGGCGGAGCCTATGGAAAAACGCCAGCAACGCGGCCTTTTTACGGTTCCTG
GCCTTTTGCTGGCCTTTTGCTCACATGTTCTTTCCTGCGTTATCCCCTGATTCTGTGGATAACCGTATTACCGCCTTTGAGTGAGCTGATACCGCTCGCCGCAGCCG
AACGACCGAGCGCAGCGAGTCAGTGAGCGAGGAAGCGGAAGAGCGCCTGATGCGGTATTTTCTCCTTACGCATCTGTGCGGTATTTCACACCGCAATGGTGCACTCT
CAGTACAATCTGCTCTGATGCCGCATAGTTAAGCCAGTATACACTCCGCTATCGCTACGTGACTGGGTCATGGCTGCGCCCCGACACCCGCCAACACCCGCTGACGC
GCCCTGACGGGCTTGTCTGCTCCCGGCATCCGCTTACAGACAAGCTGTGACCGTCTCCGGGAGCTGCATGTGTCAGAGGTTTTCACCGTCATCACCGAAACGCGCGA
GGCAGCTGCGGTAAAGCTCATCAGCGTGGTCGTGAAGCGATTCACAGATGTCTGCCTGTTCATCCGCGTCCAGCTCGTTGAGTTTCTCCAGAAGCGTTAATGTCTGG
CTTCTGATAAAGCGGGCCATGTTAAGGGCGGTTTTTTCCTGTTTGGTCACTGATGCCTCCGTGTAAGGGGGATTTCTGTTCATGGGGGTAATGATACCGATGAAACG
AGAGAGGATGCTCACGATACGGGTTACTGATGATGAACATGCCCGGTTACTGGAACGTTGTGAGGGTAAACAACTGGCGGTATGGATGCGGCGGGACCAGAGAAAAA
TCACTCAGGGTCAATGCCAGCGCTTCGTTAATACAGATGTAGGTGTTCCACAGGGTAGCCAGCAGCATCCTGCGATGCAGATCCGGAACATAATGGTGCAGGGCGCT
GACTTCCGCGTTTCCAGACTTTACGAAACACGGAAACCGAAGACCATTCATGTTGTTGCTCAGGTCGCAGACGTTTTGCAGCAGCAGTCGCTTCACGTTCGCTCGCG
TATCGGTGATTCATTCTGCTAACCAGTAAGGCAACCCCGCCAGCCTAGCCGGGTCCTCAACGACAGGAGCACGATCATGCGCACCCGTGGGGCCGCCATGCCGGCGA
TAATGGCCTGCTTCTCGCCGAAACGTTTGGTGGCGGGACCAGTGACGAAGGCTTGAGCGAGGGCGTGCAAGATTCCGAATACCGCAAGCGACAGGCCGATCATCGTC
GCGCTCCAGCGAAAGCGGTCCTCGCCGAAAATGACCCAGAGCGCTGCCGGCACCTGTCCTACGAGTTGCATGATAAAGAAGACAGTCATAAGTGCGGCGACGATAGT
CATGCCCCGCGCCCACCGGAAGGAGCTGACTGGGTTGAAGGCTCTCAAGGGCATCGGTCGAGATCCCGGTGCCTAATGAGTGAGCTAACTTACATTAATTGCGTTGC
GCTCACTGCCCGCTTTCCAGTCGGGAAACCTGTCGTGCCAGCTGCATTAATGAATCGGCCAACGCGCGGGGAGAGGCGGTTTGCGTATTGGGCGCCAGGGTGGTTTT
TCTTTTCACCAGTGAGACGGGCAACAGCTGATTGCCCTTCACCGCCTGGCCCTGAGAGAGTTGCAGCAAGCGGTCCACGCTGGTTTGCCCCAGCAGGCGAAAATCCT
GTTTGATGGTGGTTAACGGCGGGATATAACATGAGCTGTCTTCGGTATCGTCGTATCCCACTACCGAGATATCCGCACCAACGCGCAGCCCGGACTCGGTAATGGCG
CGCATTGCGCCCAGCGCCATCTGATCGTTGGCAACCAGCATCGCAGTGGGAACGATGCCCTCATTCAGCATTTGCATGGTTTGTTGAAAACCGGACATGGCACTCCA
GTCGCCTTCCCGTTCCGCTATCGGCTGAATTTGATTGCGAGTGAGATATTTATGCCAGCCAGCCAGACGCAGACGCGCCGAGACAGAACTTAATGGGCCC

His Tag
Avi Tag
hPADI4
C166F variant

hPADI4_E167A plasmid

```
CATTCGTGATTGCGCCTGAGCGAGACGAAATACGCGATCGCTGTTAAAAGGACAATTACAAACAGGAATCGAATGCAACCGGCGCAGGAACACTGCCAGCGCATCAA
CAATATTTTCACCTGAATCAGGATATTCTTCTAATACCTGGAATGCTGTTTTCCCGGGGATCGCAGTGGTGAGTAACCATGCATCATCAGGAGTACGGATAAAATGC
TTGATGGTCGGAAGAGGCATAAATTCCGTCAGCCAGTTTAGTCTGACCATCTCATCTGTAACATCATTGGCAACGCTACCTTTGCCATGTTTCAGAAACAACTCTGG
CGCATCGGGCTTCCCATACAATCGATAGATTGTCGCACCTGATTGCCCGACATTATCGCGAGCCCATTTATACCCATATAAATCAGCATCCATGTTGGAATTTAATC
GCGGCCTAGAGCAAGACGTTTCCCGTTGAATATGGCTCATAACACCCCTTGTATTACTGTTTATGTAAGCAGACAGTTTTATTGTTCATGACCAAAATCCCTTAACG
TGAGTTTTCGTTCCACTGAGCGTCAGACCCCGTAGAAAAGATCAAAGGATCTTCTTGAGATCCTTTTTTTCTGCGCGTAATCTGCTGCTTGCAAACAAAAAAACCAC
CGCTACCAGCGGTGGTTTGTTTGCCGGATCAAGAGCTACCAACTCTTTTTCCGAAGGTAACTGGCTTCAGCAGAGCGCAGATACCAAATACTGTCCTTCTAGTGTAG
CCGTAGTTAGGCCACCACTTCAAGAACTCTGTAGCACCGCCTACATACCTCGCTCTGCTAATCCTGTTACCAGTGGCTGCTGCCAGTGGCGATAAGTCGTGTCTTAC
CGGGTTGGACTCAAGACGATAGTTACCGGATAAGGCGCAGCGGTCGGGCTGAACGGGGGGTTCGTGCACACAGCCCAGCTTGGAGCGAACGACCTACACCGAACTGA
GATACCTACAGCGTGAGCTATGAGAAAGCGCCACGCTTCCCGAAGGGAGAAAGGCGGACAGGTATCCGGTAAGCGGCAGGGTCGGAACAGGAGAGCGCACGAGGGAG
CTTCCAGGGGGAAACGCCTGGTATCTTTATAGTCCTGTCGGGTTTCGCCACCTCTGACTTGAGCGTCGATTTTTGTGATGCTCGTCAGGGGGGCGGAGCCTATGGAA
AAACGCCAGCAACGCGGCCTTTTTACGGTTCCTGGCCTTTTGCTGGCCTTTTGCTCACATGTTCTTTCCTGCGTTATCCCCTGATTCTGTGGATAACCGTATTACCG
CCTTTGAGTGAGCTGATACCGCTCGCCGCAGCCGAACGACCGAGCGCAGCGAGTCAGTGAGCGAGGAAGCGGAAGAGCGCCTGATGCGGTATTTTCTCCTTACGCAT
CTGTGCGGTATTTCACACCGCAATGGTGCACTCTCAGTACAATCTGCTCTGATGCCGCATAGTTAAGCCAGTATACACTCCGCTATCGCTACGTGACTGGGTCATGG
CTGCGCCCCGACACCCGCCAACACCCGCTGACGCGCCCTGACGGGCTTGTCTGCTCCCGGCATCCGCTTACAGACAAGCTGTGACCGTCTCCGGGAGCTGCATGTGT
CAGAGGTTTTCACCGTCATCACCGAAACGCGCGAGGCAGCTGCGGTAAAGCTCATCAGCGTGGTCGTGAAGCGATTCACAGATGTCTGCCTGTTCATCCGCGTCCAG
CTCGTTGAGTTTCTCCAGAAGCGTTAATGTCTGGCTTCTGATAAAGCGGGCCATGTTAAGGGCGGTTTTTTCCTGTTTGGTCACTGATGCCTCCGTGTAAGGGGGAT
TTCTGTTCATGGGGGTAATGATACCGATGAAACGAGAGAGGATGCTCACGATACGGGTTACTGATGATGAACATGCCCGGTTACTGGAACGTTGTGAGGGTAAACAA
CTGGCGGTATGGATGCGGCGGGACCAGAGAAAAATCACTCAGGGTCAATGCCAGCGCTTCGTTAATACAGATGTAGGTGTTCCACAGGGTAGCCAGCAGCATCCTGC
GATGCAGATCCGGAACATAATGGTGCAGGGCGCTGACTTCCGCGTTTCCAGACTTTACGAAACACGGAAACCGAAGACCATTCATGTTGTTGCTCAGGTCGCAGACG
TTTTGCAGCAGCAGTCGCTTCACGTTCGCTCGCGTATCGGTGATTCATTCTGCTAACCAGTAAGGCAACCCCGCCAGCCTAGCCGGGTCCTCAACGACAGGAGCACG
ATCATGCGCACCCGTGGGGCCGCCATGCCGGCGATAATGGCCTGCTTCTCGCCGAAACGTTTGGTGGCGGGACCAGTGACGAAGGCTTGAGCGAGGGCGTGCAAGAT
TCCGAATACCGCAAGCGACAGGCCGATCATCGTCGCGCTCCAGCGAAAGCGGTCCTCGCCGAAAATGACCCAGAGCGCTGCCGGCACCTGTCCTACGAGTTGCATGA
TAAAGAAGACAGTCATAAGTGCGGCGACGATAGTCATGCCCCGCGCCCACCGGAAGGAGCTGACTGGGTTGAAGGCTCTCAAGGGCATCGGTCGAGATCCCGGTGCC
TAATGAGTGAGCTAACTTACATTAATTGCGTTGCGCTCACTGCCCGCTTTCCAGTCGGGAAACCTGTCGTGCCAGCTGCATTAATGAATCGGCCAACGCGCGGGGAG
AGGCGGTTTGCGTATTGGGCGCCAGGGTGGTTTTTCTTTTCACCAGTGAGACGGGCAACAGCTGATTGCCCTTCACCGCCTGGCCCTGAGAGAGTTGCAGCAAGCGG
TCCACGCTGGTTTGCCCCAGCAGGCGAAAATCCTGTTTGATGGTGGTTAACGGCGGGATATAACATGAGCTGTCTTCGGTATCGTCGTATCCCACTACCGAGATATC
CGCACCAACGCGCAGCCCGGACTCGGTAATGGCGCGCATTGCGCCCAGCGCCATCTGATCGTTGGCAACCAGCATCGCAGTGGGAACGATGCCCTCATTCAGCATTT
GCATGGTTTGTTGAAAACCGGACATGGCACTCCAGTCGCCTTCCCGTTCCGCTATCGGCTGAATTTGATTGCGAGTGAGATATTTATGCCAGCCAGCCAGACGCAGA
CGCGCCGAGACAGAACTTAATGGGCCCGCTAACAGCGCGATTTGCTGGTGACCCAATGCGACCAGATGCTCCACGCCCAGTCGCGTACCGTCTTCATGGGAGAAAAT
AATACTGTTGATGGGTGTCTGGTCAGAGACATCAAGAAATAACGCCGGAACATTAGTGCAGGCAGCTTCCACAGCAATGGCATCCTGGTCATCCAGCGGATAGTTAA
TGATCAGCCCACTGACGCGTTGCGCGAGAAGATTGTGCACCGCCGCTTTACAGGCTTCGACGCCGCTTCGTTCTACCATCGACACCACCACGCTGGCACCCAGTTGA
TCGGCGCGAGATTTAATCGCCGCGACAATTTGCGACGGCGCGTGCAGGGCCAGACTGGAGGTGGCAACGCCAATCAGCAACGACTGTTTGCCCGCCAGTTGTTGTGC
CACGCGGTTGGGAATGTAATTCAGCTCCGCCATCGCCGCTTCCACTTTTTCCCGCGTTTTCGCAGAAACGTGGCTGGCCTGGTTCACCACGCGGGAAACGGTCTGAT
AAGAGACACCGGCATACTCTGCGACATCGTATAACGTTACTGGTTTCACATTCACCACCCTGAATTGACTCTCTTCCGGGCGCTATCATGCCATACCGCGAAAGGTT
TTGCGCCATTCGATGGTGTCCGGGATCTCGACGCTCTCCCTTATGCGACTCCTGCATTAGGAAGCAGCCCAGTAGTAGGTTGAGGCCGTTGAGCACCGCCGCCGCAA
GGAATGGTGCATGCAAGGAGATGGCGCCCAACAGTCCCCCGGCCACGGGGCCTGCCACCATACCCACGCCGAAACAAGCGCTCATGAGCCCGAAGTGGCGAGCCCGA
TCTTCCCCATCGGTGATGTCGGCGATATAGGCGCCAGCAACCGCACCTGTGGCGCCGGTGATGCCGGCCACGATGCGTCCGGCGTAGAGGATCGAGATCTCGATCCC
GCGAAATTAATACGACTCACTATAGGGGAATTGTGAGCGGATAACAATTCCCCTCTAGAAATAATTTTGTTTAACTTTAAGAAGGAGATATACCATGAGCGGCCTGA
ACGATATTTTTGAAGCGCAGAAAATTGAATGGCATGAAGGCAGCGCTGGAGGTTCAGGTCATCACCACCATCATCACGGTAGTGGTGGAATGGCCCAGGGGACATTG
ATCCGTGTGACCCCAGAGCAGCCCACCCATGCCGTGTGTGTGCTGGGCACCTTGACTCAGCTTGACATCTGCAGCTCTGCCCCTGAGGACTGCACGTCCTTCAGCAT
CAACGCCTCCCCAGGGGTGGTCGTGGATATTGCCCACAGCCCTCCAGCCAAGAAGAAATCCACAGGTTCCTCCACATGGCCCCTGGACCCTGGGGTAGAGGTGACCC
TGACGATGAAAGCGGCCAGTGGTAGCACAGGCGACCAGAAGGTTCAGATTTCATACTACGGACCCAAGACTCCACCAGTCAAAGCTCTACTCTACCTCACCGCGGTG
GAAATCTCCCTGTGCGCAGACATCACCCGCACCGGCAAAGTGAAGCCAACCAGAGCTGTGAAAGATCAGAGGACCTGGACCTGGGGCCCTTGTGGACAGGGTGCCAT
CCTGCTGGTGAACTGTGACAGAGACAATCTCGAATCTTCTGCCATGGACTGCGCGGATGATGAAGTGCTTGACAGCGAAGACCTGCAGGACATGTCGCTGATGACCC
TGAGCACGAAGACCCCCAAGGACTTCTTCACAAACCATACACTGGTGCTCCACGTGGCCAGGTCTGAGATGGACAAAGTGAGGGTGTTTCAGGCCACACGGGGCAAA
CTGTCCTCCAAGTGCAGCGTAGTCTTGGGTCCCAAGTGGCCCTCTCACTACCTGATGGTCCCCGGTGGAAAGCACAACATGGACTTCTACGTGGAGGCCCTCGCTTT
CCCGGACACCGACTTCCCGGGGCTCATTACCCTCACCATCTCCCTGCTGGACACGTCCAACCTGGAGCTCCCCGAGGCTGTGGTGTTCCAAGACAGCGTGGTCTTCC
GCGTGGCGCCCTGGATCATGACCCCCAACACCCAGCCCCCGCAGGAGGTGTACGCGTGCAGTATTTTTGAAAATGAGGACTTCCTGAAGTCAGTGACTACTCTGGCC
ATGAAAGCCAAGTGCAAGCTGACCATCTGCCCTGAGGAGGAGAACATGGATGACCAGTGGATGCAGGATGAAATGGAGATCGGCTACATCCAAGCCCCACACAAAAC
GCTGCCCGTGGTCTTCGACTCTCCAAGGAACAGAGGCCTGAAGGAGTTTCCCATCAAACGAGTGATGGGTCCAGATTTTGGCTATGTAACTCGAGGGCCCCAAACAG
GGGGTATCAGTGGACTGGACTCCTTTGGGAACCTGGAAGTGAGCCCCCCAGTCACAGTCAGGGGCAAGGAATACCCGCTGGGCAGGATTCTCTTCGGGGACAGCTGT
TATCCCAGCAATGACAGCCGGCAGATGCACCAGGCCCTACAGGACTTCCTCAGTGCCCAGCAGGTGCAGGCCCCTGTGAAGCTCTATTCTGACTGGCTGTCCGTGGG
CCACGTGGACGAGTTCCTGAGCTTTGTGCCAGCACCCGACAGGAAGGGCTTCCGGCTGCTCCTGGCCAGCCCCAGGTCCTGCTACAAACTGTTCCAGGAGCAGCAGA
ATGAGGGCCACGGGGAGGCCCTGCTGTTCGAAGGGATCAAGAAAAAAAAACAGCAGAAAATAAAGAACATTCTGTCAAACAAGACATTGAGAGAACATAATTCATTT
GTGGAGAGATGCATCGACTGGAACCGCGAGCTGCTGAAGCGGGAGCTGGGCCTGGCCGAGAGTGACATCATTGACATCCCGCAGCTCTTCAAGCTCAAAGAGTTCTC
TAAGGCGGAAGCTTTTTTCCCCAACATGGTGAACATGCTGGTGCTAGGGAAGCACCTGGGCATCCCCAAGCCCTTCGGGCCCGTCATCAACGGCCGCTGCTGCCTGG
AGGAGAAGGTGTGTTCCCTGCTGGAGCCACTGGGCCTCCAGTGCACCTTCATCAACGACTTCTTCACCTACCACATCAGGCATGGGGAGGTGCACTGCGGCACCAAC
GTGCGCAGAAAGCCCTTCTCCTTCAAGTGGTGGAACATGGTGCCCTGAGGTGGAAGTGGTCATCACCACCATCATCACTGATGACGAAGCTTGCGGCCGCACTCGAG
CACCACCACCACCACCACTGAGATCCGGCTGCTAACAAAGCCCGAAAGGAAGCTGAGTTGGCTGCTGCCACCGCTGAGCAATAACTAGCATAACCCCTTGGGGCCTC
TAAACGGGTCTTGAGGGGTTTTTTGCTGAAAGGAGGAACTATATCCGGATTGGCGAATGGGACGCGCCCTGTAGCGGCGCATTAAGCGCGGCGGGTGTGGTGGTTAC
GCGCAGCGTGACCGCTACACTTGCCAGCGCCCTAGCGCCCGCTCCTTTCGCTTTCTTCCCTTCCTTTCTCGCCACGTTCGCCGGCTTTCCCCGTCAAGCTCTAAATC
GGGGGCTCCCTTTAGGGTTCCGATTTAGTGCTTTACGGCACCTCGACCCCAAAAAACTTGATTAGGGTGATGGTTCACGTAGTGGGCCATCGCCCTGATAGACGGTT
TTTCGCCCTTTGACGTTGGAGTCCACGTTCTTTAATAGTGGACTCTTGTTCCAAACTGGAACAACACTCAACCCTATCTCGGTCTATTCTTTTGATTTATAAGGGAT
TTTGCCGATTTCGGCCTATTGGTTAAAAAATGAGCTGATTTAACAAAAATTTAACGCGAATTTTAACAAAATATTAACGCTTACAATTTAGGTGGCACTTTTCGGGG
AAATGTGCGCGGAACCCCTATTTGTTTATTTTTCTAAATACATTCAAATATGTATCCGCTCATGAATTAATTCTTAGAAAAACTCATCGAGCATCAAATGAAACTGC
AATTTATTCATATCAGGATTATCAATACCATATTTTTGAAAAAGCCGTTTCTGTAATGAAGGAGAAAACTCACCGAGGCAGTTCCATAGGATGGCAAGATCCTGGTA
TCGGTCTGCGATTCCGACTCGTCCAACATCAATACAACCTATTAATTTCCCCTCGTCAAAAATAAGGTTATCAAGTGAGAAATCACCATGAGTGACGACTGAATCCG
GTGAGAATGGCAAAAGTTTATGCATTTCTTTCCAGACTTGTTCAACAGGCCAGCCATTACGCTCGTCATCAAAATCACTCGCATCAACCAAACCGTTATT
```

His Tag
Avi Tag
hPADI4
E167A variant

hPADI4_D168A plasmid

```
TCCACTTTTTCCCGCGTTTTCGCAGAAACGTGGCTGGCCTGGTTCACCACGCGGGAAACGGTCTGATAAGAGACACCGGCATACTCTGCGACATCGTATAACGTTAC
TGGTTTCACATTCACCACCCTGAATTGACTCTCTTCCGGGCGCTATCATGCCATACCGCGAAAGGTTTTGCGCCATTCGATGGTGTCCGGGATCTCGACGCTCTCCC
TTATGCGACTCCTGCATTAGGAAGCAGCCCAGTAGTAGGTTGAGGCCGTTGAGCACCGCCGCCGCAAGGAATGGTGCATGCAAGGAGATGGCGCCCAACAGTCCCCC
GGCCACGGGGCCTGCCACCATACCCACGCCGAAACAAGCGCTCATGAGCCCGAAGTGGCGAGCCCGATCTTCCCCATCGGTGATGTCGGCGATATAGGCGCCAGCAA
CCGCACCTGTGGCGCCGGTGATGCCGGCCACGATGCGTCCGGCGTAGAGGATCGAGATCTCGATCCCGCGAAATTAATACGACTCACTATAGGGGAATTGTGAGCGG
ATAACAATTCCCCTCTAGAAATAATTTTGTTTAACTTTAAGAAGGAGATATACCATGAGCGGCCTGAACGATATTTTTGAAGCGCAGAAAATTGAATGGCATGAAGG
CAGCGCTGGAGGTTCAGGTCATCACCACCATCATCACGGTAGTGGTGGAATGGCCCAGGGGACATTGATCCGTGTGACCCCAGAGCAGCCCACCCATGCCGTGTGTG
TGCTGGGCACCTTGACTCAGCTTGACATCTGCAGCTCTGCCCCTGAGGACTGCACGTCCTTCAGCATCAACGCCTCCCCAGGGGTGGTCGTGGATATTGCCCACAGC
CCTCCAGCCAAGAAGAAATCCACAGGTTCCTCCACATGGCCCCTGGACCCTGGGGTAGAGGTGACCCTGACGATGAAAGCGGCCAGTGGTAGCACAGGCGACCAGAA
GGTTCAGATTTCATACTACGGACCCAAGACTCCACCAGTCAAAGCTCTACTCTACCTCACCGCGGTGGAAATCTCCCTGTGCGCAGACATCACCCGCACCGGCAAAG
TGAAGCCAACCAGAGCTGTGAAAGATCAGAGGACCTGGACCTGGGGCCCTTGTGGACAGGGTGCCATCCTGCTGGTGAACTGTGACAGAGACAATCTCGAATCTTCT
GCCATGGACTGCGAGGCTGATGAAGTGCTTGACAGCGAAGACCTGCAGGACATGTCGCTGATGACCCTGAGCACGAAGACCCCCAAGGACTTCTTCACAAACCATAC
ACTGGTGCTCCACGTGGCCAGGTCTGAGATGGACAAAGTGAGGGTGTTTCAGGCCACACGGGGCAAACTGTCCTCCAAGTGCAGCGTAGTCTTGGGTCCCAAGTGGC
CCTCTCACTACCTGATGGTCCCCGGTGGAAAGCACAACATGGACTTCTACGTGGAGGCCCTCGCTTTCCCGGACACCGACTTCCCGGGGCTCATTACCCTCACCATC
TCCCTGCTGGACACGTCCAACCTGGAGCTCCCCGAGGCTGTGGTGTTCCAAGACAGCGTGGTCTTCCGCGTGGCGCCCTGGATCATGACCCCCAACACCCAGCCCCC
GCAGGAGGTGTACGCGTGCAGTATTTTTGAAAATGAGGACTTCCTGAAGTCAGTGACTACTCTGGCCATGAAAGCCAAGTGCAAGCTGACCATCTGCCCTGAGGAGG
AGAACATGGATGACCAGTGGATGCAGGATGAAATGGAGATcGGCTACATCCAAGCCCCACACAAAACGCTGCCCGTGGTCTTCGACTCTCCAAGGAACAGAGGCCTG
AAGGAGTTTCCCATCAAACGAGTGATGGGTCCAGATTTTGGCTATGTAACTCGAGGGCCCCAAACAGGGGGTATCAGTGGACTGGACTCCTTTGGGAACCTGGAAGT
GAGCCCCCCAGTCACAGTCAGGGGCAAGGAATACCCGCTGGGCAGGATTCTCTTCGGGGACAGCTGTTATCCCAGCAATGACAGCCGGCAGATGCACCAGGCCCTAC
AGGACTTCCTCAGTGCCCAGCAGGTGCAGGCCCCTGTGAAGCTCTATTCTGACTGGCTGTCCGTGGGCCACGTGGACGAGTTCCTGAGCTTTGTGCCAGCACCCGAC
AGGAAGGGCTTCCGGCTGCTCCTGGCCAGCCCCAGGTCCTGCTACAAACTGTTCCAGGAGCAGCAGAATGAGGGCCACGGGGAGGCCCTGCTGTTCGAAGGGATCAA
GAAAAAAAAACAGCAGAAAATAAAGAACATTCTGTCAAACAAGACATTGAGAGAACATAATTCATTTGTGGAGAGATGCATCGACTGGAACCGCGAGCTGCTGAAGC
GGGAGCTGGGCCTGGCCGAGAGTGACATCATTGACATCCCGCAGCTCTTCAAGCTCAAAGAGTTCTCTAAGGCGGAAGCTTTTTTCCCCAACATGGTGAACATGCTG
GTGCTAGGGAAGCACCTGGGCATCCCCAAGCCCTTCGGGCCCGTCATCAACGGCCGCTGCTGCCTGGAGGAGAAGGTGTGTTCCCTGCTGGAGCCACTGGGCCTCCA
GTGCACCTTCATCAACGACTTCTTCACCTACCACATCAGGCATGGGGAGGTGCACTGCGGCACCAACGTGCGCAGAAAGCCCTTCTCCTTCAAGTGGTGGAACATGG
TGCCCTGAGGTGGAAGTGGTCATCACCACCATCATCACTGATGACGAAGCTTGCGGCCGCACTCGAGCACCACCACCACCACCACTGAGATCCGGCTGCTAACAAAG
CCCGAAAGGAAGCTGAGTTGGCTGCTGCCACCGCTGAGCAATAACTAGCATAACCCCTTGGGGCCTCTAAACGGGTCTTGAGGGGTTTTTTGCTGAAAGGAGGAACT
ATATCCGGATTGGCGAATGGGACGCGCCCTGTAGCGGCGCATTAAGCGCGGCGGGTGTGGTGGTTACGCGCAGCGTGACCGCTACACTTGCCAGCGCCCTAGCGCCC
GCTCCTTTCGCTTTCTTCCCTTCCTTTCTCGCCACGTTCGCCGGCTTTCCCCGTCAAGCTCTAAATCGGGGGCTCCCTTTAGGGTTCCGATTTAGTGCTTTACGGCA
CCTCGACCCCAAAAAACTTGATTAGGGTGATGGTTCACGTAGTGGGCCATCGCCCTGATAGACGGTTTTTCGCCCTTTGACGTTGGAGTCCACGTTCTTTAATAGTG
GACTCTTGTTCCAAACTGGAACAACACTCAACCCTATCTCGGTCTATTCTTTTGATTTATAAGGGATTTTGCCGATTTCGGCCTATTGGTTAAAAAATGAGCTGATT
TAACAAAAATTTAACGCGAATTTTAACAAAATATTAACGCTTACAATTTAGGTGGCACTTTTCGGGGAAATGTGCGCGGAACCCCTATTTGTTTATTTTTCTAAATA
CATTCAAATATGTATCCGCTCATGAATTAATTCTTAGAAAAACTCATCGAGCATCAAATGAAACTGCAATTTATTCATATCAGGATTATCAATACCATATTTTTGAA
AAAGCCGTTTCTGTAATGAAGGAGAAAACTCACCGAGGCAGTTCCATAGGATGGCAAGATCCTGGTATCGGTCTGCGATTCCGACTCGTCCAACATCAATACAACCT
ATTAATTTCCCCTCGTCAAAAATAAGGTTATCAAGTGAGAAATCACCATGAGTGACGACTGAATCCGGTGAGAATGGCAAAAGTTTATGCATTTCTTTCCAGACTTG
TTCAACAGGCCAGCCATTACGCTCGTCATCAAAATCACTCGCATCAACCAAACCGTTATTCATTCGTGATTGCGCCTGAGCGAGACGAAATACGCGATCGCTGTTAA
AAGGACAATTACAAACAGGAATCGAATGCAACCGGCGCAGGAACACTGCCAGCGCATCAACAATATTTTCACCTGAATCAGGATATTCTTCTAATACCTGGAATGCT
GTTTTCCCGGGGATCGCAGTGGTGAGTAACCATGCATCATCAGGAGTACGGATAAAATGCTTGATGGTCGGAAGAGGCATAAATTCCGTCAGCCAGTTTAGTCTGAC
CATCTCATCTGTAACATCATTGGCAACGCTACCTTTGCCATGTTTCAGAAACAACTCTGGCGCATCGGGCTTCCCATACAATCGATAGATTGTCGCACCTGATTGCC
CGACATTATCGCGAGCCCATTTATACCCATATAAATCAGCATCCATGTTGGAATTTAATCGCGGCCTAGAGCAAGACGTTTCCCGTTGAATATGGCTCATAACACCC
CTTGTATTACTGTTTATGTAAGCAGACAGTTTTATTGTTCATGACCAAAATCCCTTAACGTGAGTTTTCGTTCCACTGAGCGTCAGACCCCGTAGAAAAGATCAAAG
GATCTTCTTGAGATCCTTTTTTTCTGCGCGTAATCTGCTGCTTGCAAACAAAAAAACCACCGCTACCAGCGGTGGTTTGTTTGCCGGATCAAGAGCTACCAACTCTT
TTTCCGAAGGTAACTGGCTTCAGCAGAGCGCAGATACCAAATACTGTCCTTCTAGTGTAGCCGTAGTTAGGCCACCACTTCAAGAACTCTGTAGCACCGCCTACATA
CCTCGCTCTGCTAATCCTGTTACCAGTGGCTGCTGCCAGTGGCGATAAGTCGTGTCTTACCGGGTTGGACTCAAGACGATAGTTACCGGATAAGGCGCAGCGGTCGG
GCTGAACGGGGGGTTCGTGCACACAGCCCAGCTTGGAGCGAACGACCTACACCGAACTGAGATACCTACAGCGTGAGCTATGAGAAAGCGCCACGCTTCCCGAAGGG
AGAAAGGCGGACAGGTATCCGGTAAGCGGCAGGGTCGGAACAGGAGAGCGCACGAGGGAGCTTCCAGGGGGAAACGCCTGGTATCTTTATAGTCCTGTCGGGTTTCG
CCACCTCTGACTTGAGCGTCGATTTTTGTGATGCTCGTCAGGGGGGCGGAGCCTATGGAAAAACGCCAGCAACGCGGCCTTTTTACGGTTCCTGGCCTTTTGCTGGC
CTTTTGCTCACATGTTCTTTCCTGCGTTATCCCCTGATTCTGTGGATAACCGTATTACCGCCTTTGAGTGAGCTGATACCGCTCGCCGCAGCCGAACGACCGAGCGC
AGCGAGTCAGTGAGCGAGGAAGCGGAAGAGCGCCTGATGCGGTATTTTCTCCTTACGCATCTGTGCGGTATTTCACACCGCAATGGTGCACTCTCAGTACAATCTGC
TCTGATGCCGCATAGTTAAGCCAGTATACACTCCGCTATCGCTACGTGACTGGGTCATGGCTGCGCCCCGACACCCGCCAACACCCGCTGACGCGCCCTGACGGGCT
TGTCTGCTCCCGGCATCCGCTTACAGACAAGCTGTGACCGTCTCCGGGAGCTGCATGTGTCAGAGGTTTTCACCGTCATCACCGAAACGCGCGAGGCAGCTGCGGTA
AAGCTCATCAGCGTGGTCGTGAAGCGATTCACAGATGTCTGCCTGTTCATCCGCGTCCAGCTCGTTGAGTTTCTCCAGAAGCGTTAATGTCTGGCTTCTGATAAAGC
GGGCCATGTTAAGGGCGGTTTTTTCCTGTTTGGTCACTGATGCCTCCGTGTAAGGGGGATTTCTGTTCATGGGGGTAATGATACCGATGAAACGAGAGAGGATGCTC
ACGATACGGGTTACTGATGATGAACATGCCCGGTTACTGGAACGTTGTGAGGGTAAACAACTGGCGGTATGGATGCGGCGGGACCAGAGAAAAATCACTCAGGGTCA
ATGCCAGCGCTTCGTTAATACAGATGTAGGTGTTCCACAGGGTAGCCAGCAGCATCCTGCGATGCAGATCCGGAACATAATGGTGCAGGGCGCTGACTTCCGCGTTT
CCAGACTTTACGAAACACGGAAACCGAAGACCATTCATGTTGTTGCTCAGGTCGCAGACGTTTTGCAGCAGCAGTCGCTTCACGTTCGCTCGCGTATCGGTGATTCA
TTCTGCTAACCAGTAAGGCAACCCCGCCAGCCTAGCCGGGTCCTCAACGACAGGAGCACGATCATGCGCACCCGTGGGGCCGCCATGCCGGCGATAATGGCCTGCTT
CTCGCCGAAACGTTTGGTGGCGGGACCAGTGACGAAGGCTTGAGCGAGGGCGTGCAAGATTCCGAATACCGCAAGCGACAGGCCGATCATCGTCGCGCTCCAGCGAA
AGCGGTCCTCGCCGAAAATGACCCAGAGCGCTGCCGGCACCTGTCCTACGAGTTGCATGATAAAGAAGACAGTCATAAGTGCGGCGACGATAGTCATGCCCCGCGCC
CACCGGAAGGAGCTGACTGGGTTGAAGGCTCTCAAGGGCATCGGTCGAGATCCCGGTGCCTAATGAGTGAGCTAACTTACATTAATTGCGTTGCGCTCACTGCCCGC
TTTCCAGTCGGGAAACCTGTCGTGCCAGCTGCATTAATGAATCGGCCAACGCGCGGGGAGAGGCGGTTTGCGTATTGGGCGCCAGGGTGGTTTTTCTTTTCACCAGT
GAGACGGGCAACAGCTGATTGCCCTTCACCGCCTGGCCCTGAGAGAGTTGCAGCAAGCGGTCCACGCTGGTTTGCCCCAGCAGGCGAAAATCCTGTTTGATGGTGGT
TAACGGCGGGATATAACATGAGCTGTCTTCGGTATCGTCGTATCCCACTACCGAGATATCCGCACCAACGCGCAGCCCGGACTCGGTAATGGCGCGCATTGCGCCCA
GCGCCATCTGATCGTTGGCAACCAGCATCGCAGTGGGAACGATGCCCTCATTCAGCATTTGCATGGTTTGTTGAAAACCGGACATGGCACTCCAGTCGCCTTCCCGT
TCCGCTATCGGCTGAATTTGATTGCGAGTGAGATATTTATGCCAGCCAGCCAGACGCAGACGCGCCGAGACAGAACTTAATGGGCCCGCTAACAGCGCGATTTGCTG
GTGACCCAATGCGACCAGATGCTCCACGCCCAGTCGCGTACCGTCTTCATGGGAGAAAATAATACTGTTGATGGGTGTCTGGTCAGAGACATCAAGAAATAACGCCG
GAACATTAGTGCAGGCAGCTTCCACAGCAATGGCATCCTGGTCATCCAGCGGATAGTTAATGATCAGCCCACTGACGCGTTGCGCGAGAAGATTGTGCACCGCCGCT
TTACAGGCTTCGACGCCGCTTCGTTCTACCATCGACACCACCACGCTGGCACCCAGTTGATCGGCGCGAGATTTAATCGCCGCGACAATTTGCGACGGCGCGTGCAG
GGCCAGACTGGAGGTGGCAACGCCAATCAGCAACGACTGTTTGCCCGCCAGTTGTTGTGCCACGCGGTTGGGAATGTAATTCAGCTCCGCCATCGCCGCT
```

His Tag
Avi Tag
hPADI4
D168A variant

hPADI4_N373A plasmid

TTTTCCCGCGTTTTCGCAGAAACGTGGCTGGCCTGGTTCACCACGCGGGAAACGGTCTGATAAGAGACACCGGCATACTCTGCGACATCGTATAACGTTACTGGTTT
CACATTCACCACCCTGAATTGACTCTCTTCCGGGCGCTATCATGCCATACCGCGAAAGGTTTTGCGCCATTCGATGGTGTCCGGGATCTCGACGCTCTCCCTTATGC
GACTCCTGCATTAGGAAGCAGCCCAGTAGTAGGTTGAGGCCGTTGAGCACCGCCGCCGCAAGGAATGGTGCATGCAAGGAGATGGCGCCCAACAGTCCCCCGGCCAC
GGGGCCTGCCACCATACCCACGCCGAAACAAGCGCTCATGAGCCCGAAGTGGCGAGCCCGATCTTCCCCATCGGTGATGTCGGCGATATAGGCGCCAGCAACCGCAC
CTGTGGCGCCGGTGATGCCGGCCACGATGCGTCCGGCGTAGAGGATCGAGATCTCGATCCCGCGAAATTAATACGACTCACTATAGGGGAATTGTGAGCGGATAACA
ATTCCCCTCTAGAAATAATTTTGTTTAACTTTAAGAAGGAGATATACCATGAGCGGCCTGAACGATATTTTTGAAGCGCAGAAAATTGAATGGCATGAAGGCAGCGC
TGGAGGTTCAGGTCATCACCACCATCATCACGGTAGTGGTGGAATGGCCCAGGGGACATTGATCCGTGTGACCCCAGAGCAGCCCACCCATGCCGTGTGTGTGCTGG
GCACCTTGACTCAGCTTGACATCTGCAGCTCTGCCCCTGAGGACTGCACGTCCTTCAGCATCAACGCCTCCCCAGGGGTGGTCGTGGATATTGCCCACAGCCCTCCA
GCCAAGAAGAAATCCACAGGTTCCTCCACATGGCCCCTGGACCCTGGGGTAGAGGTGACCCTGACGATGAAAGCGGCCAGTGGTAGCACAGGCGACCAGAAGGTTCA
GATTTCATACTACGGACCCAAGACTCCACCAGTCAAAGCTCTACTCTACCTCACCGCGGTGGAAATCTCCCTGTGCGCAGACATCACCCGCACCGGCAAAGTGAAGC
CAACCAGAGCTGTGAAAGATCAGAGGACCTGGACCTGGGGCCCTTGTGGACAGGGTGCCATCCTGCTGGTGAACTGTGACAGAGACAATCTCGAATCTTCTGCCATG
GACTGCGAGGATGATGAAGTGCTTGACAGCGAAGACCTGCAGGACATGTCGCTGATGACCCTGAGCACGAAGACCCCCAAGGACTTCTTCACAAACCATACACTGGT
GCTCCACGTGGCCAGGTCTGAGATGGACAAAGTGAGGGTGTTTCAGGCCACACGGGGCAAACTGTCCTCCAAGTGCAGCGTAGTCTTGGGTCCCAAGTGGCCCTCTC
ACTACCTGATGGTCCCCGGTGGAAAGCACAACATGGACTTCTACGTGGAGGCCCTCGCTTTCCCGGACACCGACTTCCCGGGGCTCATTACCCTCACCATCTCCCTG
CTGGACACGTCCAACCTGGAGCTCCCCGAGGCTGTGGTGTTCCAAGACAGCGTGGTCTTCCGCGTGGCGCCCTGGATcATGACCCCCAACACCCAGCCCCCGCAGGA
GGTGTACGCGTGCAGTATTTTTGAAAATGAGGACTTCCTGAAGTCAGTGACTACTCTGGCCATGAAAGCCAAGTGCAAGCTGACCATCTGCCCTGAGGAGGAGAACA
TGGATGACCAGTGGATGCAGGATGAAATGGAGATcGGCTACATCCAAGCCCCACACAAAACGCTGCCCGTGGTCTTCGACTCTCCAAGGGCCAGAGGCCTGAAGGAG
TTTCCCATCAAACGAGTGATGGGTCCAGATTTTGGCTATGTAACTCGAGGGCCCCAAACAGGGGGTATCAGTGGACTGGACTCCTTTGGGAACCTGGAAGTGAGCCC
CCCAGTCACAGTCAGGGGCAAGGAATACCCGCTGGGCAGGATTCTCTTCGGGGACAGCTGTTATCCCAGCAATGACAGCCGGCAGATGCACCAGGCCCTACAGGACT
TCCTCAGTGCCCAGCAGGTGCAGGCCCCTGTGAAGCTCTATTCTGACTGGCTGTCCGTGGGCCACGTGGACGAGTTCCTGAGCTTTGTGCCAGCACCCGACAGGAAG
GGCTTCCGGCTGCTCCTGGCCAGCCCCAGGTCCTGCTACAAACTGTTCCAGGAGCAGCAGAATGAGGGCCACGGGGAGGCCCTGCTGTTCGAAGGGATCAAGAAAAA
AAAACAGCAGAAAATAAAGAACATTCTGTCAAACAAGACATTGAGAGAACATAATTCATTTGTGGAGAGATGCATCGACTGGAACCGCGAGCTGCTGAAGCGGGAGC
TGGGCCTGGCCGAGAGTGACATCATTGACATCCCGCAGCTCTTCAAGCTCAAAGAGTTCTCTAAGGCGGAAGCTTTTTTCCCCAACATGGTGAACATGCTGGTGCTA
GGGAAGCACCTGGGCATCCCCAAGCCCTTCGGGCCCGTCATCAACGGCCGCTGCTGCCTGGAGGAGAAGGTGTGTTCCCTGCTGGAGCCACTGGGCCTCCAGTGCAC
CTTCATCAACGACTTCTTCACCTACCACATCAGGCATGGGGAGGTGCACTGCGGCACCAACGTGCGCAGAAAGCCCTTCTCCTTCAAGTGGTGGAACATGGTGCCCT
GAGGTGGAAGTGGTCATCACCACCATCATCACTGATGACGAAGCTTGCGGCCGCACTCGAGCACCACCACCACCACCACTGAGATCCGGCTGCTAACAAAGCCCGAA
AGGAAGCTGAGTTGGCTGCTGCCACCGCTGAGCAATAACTAGCATAACCCCTTGGGGCCTCTAAACGGGTCTTGAGGGGTTTTTTGCTGAAAGGAGGAACTATATCC
GGATTGGCGAATGGGACGCGCCCTGTAGCGGCGCATTAAGCGCGGCGGGTGTGGTGGTTACGCGCAGCGTGACCGCTACACTTGCCAGCGCCCTAGCGCCCGCTCCT
TTCGCTTTCTTCCCTTCCTTTCTCGCCACGTTCGCCGGCTTTCCCCGTCAAGCTCTAAATCGGGGGCTCCCTTTAGGGTTCCGATTTAGTGCTTTACGGCACCTCGA
CCCCAAAAAACTTGATTAGGGTGATGGTTCACGTAGTGGGCCATCGCCCTGATAGACGGTTTTTCGCCCTTTGACGTTGGAGTCCACGTTCTTTAATAGTGGACTCT
TGTTCCAAACTGGAACAACACTCAACCCTATCTCGGTCTATTCTTTTGATTTATAAGGGATTTTGCCGATTTCGGCCTATTGGTTAAAAAATGAGCTGATTTAACAA
AAATTTAACGCGAATTTTAACAAAATATTAACGCTTACAATTTAGGTGGCACTTTTCGGGGAAATGTGCGCGGAACCCCTATTTGTTTATTTTTCTAAATACATTCA
AATATGTATCCGCTCATGAATTAATTCTTAGAAAAACTCATCGAGCATCAAATGAAACTGCAATTTATTCATATCAGGATTATCAATACCATATTTTTGAAAAAGCC
GTTTCTGTAATGAAGGAGAAAACTCACCGAGGCAGTTCCATAGGATGGCAAGATCCTGGTATCGGTCTGCGATTCCGACTCGTCCAACATCAATACAACCTATTAAT
TTCCCCTCGTCAAAAATAAGGTTATCAAGTGAGAAATCACCATGAGTGACGACTGAATCCGGTGAGAATGGCAAAAGTTTATGCATTTCTTTCCAGACTTGTTCAAC
AGGCCAGCCATTACGCTCGTCATCAAAATCACTCGCATCAACCAAACCGTTATTCATTCGTGATTGCGCCTGAGCGAGACGAAATACGCGATCGCTGTTAAAAGGAC
AATTACAAACAGGAATCGAATGCAACCGGCGCAGGAACACTGCCAGCGCATCAACAATATTTTCACCTGAATCAGGATATTCTTCTAATACCTGGAATGCTGTTTTC
CCGGGGATCGCAGTGGTGAGTAACCATGCATCATCAGGAGTACGGATAAAATGCTTGATGGTCGGAAGAGGCATAAATTCCGTCAGCCAGTTTAGTCTGACCATCTC
ATCTGTAACATCATTGGCAACGCTACCTTTGCCATGTTTCAGAAACAACTCTGGCGCATCGGGCTTCCCATACAATCGATAGATTGTCGCACCTGATTGCCCGACAT
TATCGCGAGCCCATTTATACCCATATAAATCAGCATCCATGTTGGAATTTAATCGCGGCCTAGAGCAAGACGTTTCCCGTTGAATATGGCTCATAACACCCCTTGTA
TTACTGTTTATGTAAGCAGACAGTTTTATTGTTCATGACCAAAATCCCTTAACGTGAGTTTTCGTTCCACTGAGCGTCAGACCCCGTAGAAAAGATCAAAGGATCTT
CTTGAGATCCTTTTTTTCTGCGCGTAATCTGCTGCTTGCAAACAAAAAAACCACCGCTACCAGCGGTGGTTTGTTTGCCGGATCAAGAGCTACCAACTCTTTTTCCG
AAGGTAACTGGCTTCAGCAGAGCGCAGATACCAAATACTGTCCTTCTAGTGTAGCCGTAGTTAGGCCACCACTTCAAGAACTCTGTAGCACCGCCTACATACCTCGC
TCTGCTAATCCTGTTACCAGTGGCTGCTGCCAGTGGCGATAAGTCGTGTCTTACCGGGTTGGACTCAAGACGATAGTTACCGGATAAGGCGCAGCGGTCGGGCTGAA
CGGGGGGTTCGTGCACACAGCCCAGCTTGGAGCGAACGACCTACACCGAACTGAGATACCTACAGCGTGAGCTATGAGAAAGCGCCACGCTTCCCGAAGGGAGAAAG
GCGGACAGGTATCCGGTAAGCGGCAGGGTCGGAACAGGAGAGCGCACGAGGGAGCTTCCAGGGGGAAACGCCTGGTATCTTTATAGTCCTGTCGGGTTTCGCCACCT
CTGACTTGAGCGTCGATTTTTGTGATGCTCGTCAGGGGGGCGGAGCCTATGGAAAAACGCCAGCAACGCGGCCTTTTTACGGTTCCTGGCCTTTTGCTGGCCTTTTG
CTCACATGTTCTTTCCTGCGTTATCCCCTGATTCTGTGGATAACCGTATTACCGCCTTTGAGTGAGCTGATACCGCTCGCCGCAGCCGAACGACCGAGCGCAGCGAG
TCAGTGAGCGAGGAAGCGGAAGAGCGCCTGATGCGGTATTTTCTCCTTACGCATCTGTGCGGTATTTCACACCGCAATGGTGCACTCTCAGTACAATCTGCTCTGAT
GCCGCATAGTTAAGCCAGTATACACTCCGCTATCGCTACGTGACTGGGTCATGGCTGCGCCCCGACACCCGCCAACACCCGCTGACGCGCCCTGACGGGCTTGTCTG
CTCCCGGCATCCGCTTACAGACAAGCTGTGACCGTCTCCGGGAGCTGCATGTGTCAGAGGTTTTCACCGTCATCACCGAAACGCGCGAGGCAGCTGCGGTAAAGCTC
ATCAGCGTGGTCGTGAAGCGATTCACAGATGTCTGCCTGTTCATCCGCGTCCAGCTCGTTGAGTTTCTCCAGAAGCGTTAATGTCTGGCTTCTGATAAAGCGGGCCA
TGTTAAGGGCGGTTTTTTCCTGTTTGGTCACTGATGCCTCCGTGTAAGGGGGATTTCTGTTCATGGGGGTAATGATACCGATGAAACGAGAGAGGATGCTCACGATA
CGGGTTACTGATGATGAACATGCCCGGTTACTGGAACGTTGTGAGGGTAAACAACTGGCGGTATGGATGCGGCGGGACCAGAGAAAAATCACTCAGGGTCAATGCCA
GCGCTTCGTTAATACAGATGTAGGTGTTCCACAGGGTAGCCAGCAGCATCCTGCGATGCAGATCCGGAACATAATGGTGCAGGGCGCTGACTTCCGCGTTTCCAGAC
TTTACGAAACACGGAAACCGAAGACCATTCATGTTGTTGCTCAGGTCGCAGACGTTTTGCAGCAGCAGTCGCTTCACGTTCGCTCGCGTATCGGTGATTCATTCTGC
TAACCAGTAAGGCAACCCCGCCAGCCTAGCCGGGTCCTCAACGACAGGAGCACGATCATGCGCACCCGTGGGGCCGCCATGCCGGCGATAATGGCCTGCTTCTCGCC
GAAACGTTTGGTGGCGGGACCAGTGACGAAGGCTTGAGCGAGGGCGTGCAAGATTCCGAATACCGCAAGCGACAGGCCGATCATCGTCGCGCTCCAGCGAAAGCGGT
CCTCGCCGAAAATGACCCAGAGCGCTGCCGGCACCTGTCCTACGAGTTGCATGATAAAGAAGACAGTCATAAGTGCGGCGACGATAGTCATGCCCCGCGCCCACCGG
AAGGAGCTGACTGGGTTGAAGGCTCTCAAGGGCATCGGTCGAGATCCCGGTGCCTAATGAGTGAGCTAACTTACATTAATTGCGTTGCGCTCACTGCCCGCTTTCCA
GTCGGGAAACCTGTCGTGCCAGCTGCATTAATGAATCGGCCAACGCGCGGGGAGAGGCGGTTTGCGTATTGGGCGCCAGGGTGGTTTTTCTTTTCACCAGTGAGACG
GGCAACAGCTGATTGCCCTTCACCGCCTGGCCCTGAGAGAGTTGCAGCAAGCGGTCCACGCTGGTTTGCCCCAGCAGGCGAAAATCCTGTTTGATGGTGGTTAACGG
CGGGATATAACATGAGCTGTCTTCGGTATCGTCGTATCCCACTACCGAGATATCCGCACCAACGCGCAGCCCGGACTCGGTAATGGCGCGCATTGCGCCCAGCGCCA
TCTGATCGTTGGCAACCAGCATCGCAGTGGGAACGATGCCCTCATTCAGCATTTGCATGGTTTGTTGAAAACCGGACATGGCACTCCAGTCGCCTTCCCGTTCCGCT
ATCGGCTGAATTTGATTGCGAGTGAGATATTTATGCCAGCCAGCCAGACGCAGACGCGCCGAGACAGAACTTAATGGGCCCGCTAACAGCGCGATTTGCTGGTGACC
CAATGCGACCAGATGCTCCACGCCCAGTCGCGTACCGTCTTCATGGGAGAAAATAATACTGTTGATGGGTGTCTGGTCAGAGACATCAAGAAATAACGCCGGAACAT
TAGTGCAGGCAGCTTCCACAGCAATGGCATCCTGGTCATCCAGCGGATAGTTAATGATCAGCCCACTGACGCGTTGCGCGAGAAGATTGTGCACCGCCGCTTTACAG
GCTTCGACGCCGCTTCGTTCTACCATCGACACCACCACGCTGGCACCCAGTTGATCGGCGCGAGATTTAATCGCCGCGACAATTTGCGACGGCGCGTGCAGGGCCAG
ACTGGAGGTGGCAACGCCAATCAGCAACGACTGTTTGCCCGCCAGTTGTTGTGCCACGCGGTTGGGAATGTAATTCAGCTCCGCCATCGCCGCTTCCACT

His Tag
Avi Tag
hPADI4
N373A variant

hPADI4_R394A plasmid

```
TCCCGTTGAATATGGCTCATAACACCCCTTGTATTACTGTTTATGTAAGCAGACAGTTTTATTGTTCATGACCAAAATCCCTTAACGTGAGTTTTCGTTCCACTGAG
CGTCAGACCCCGTAGAAAAGATCAAAGGATCTTCTTGAGATCCTTTTTTTCTGCGCGTAATCTGCTGCTTGCAAACAAAAAAACCACCGCTACCAGCGGTGGTTTGT
TTGCCGGATCAAGAGCTACCAACTCTTTTTCCGAAGGTAACTGGCTTCAGCAGAGCGCAGATACCAAATACTGTCCTTCTAGTGTAGCCGTAGTTAGGCCACCACTT
CAAGAACTCTGTAGCACCGCCTACATACCTCGCTCTGCTAATCCTGTTACCAGTGGCTGCTGCCAGTGGCGATAAGTCGTGTCTTACCGGGTTGGACTCAAGACGAT
AGTTACCGGATAAGGCGCAGCGGTCGGGCTGAACGGGGGGTTCGTGCACACAGCCCAGCTTGGAGCGAACGACCTACACCGAACTGAGATACCTACAGCGTGAGCTA
TGAGAAAGCGCCACGCTTCCCGAAGGGAGAAAGGCGGACAGGTATCCGGTAAGCGGCAGGGTCGGAACAGGAGAGCGCACGAGGGAGCTTCCAGGGGGAAACGCCTG
GTATCTTTATAGTCCTGTCGGGTTTCGCCACCTCTGACTTGAGCGTCGATTTTTGTGATGCTCGTCAGGGGGGCGGAGCCTATGGAAAAACGCCAGCAACGCGGCCT
TTTTACGGTTCCTGGCCTTTTGCTGGCCTTTTGCTCACATGTTCTTTCCTGCGTTATCCCCTGATTCTGTGGATAACCGTATTACCGCCTTTGAGTGAGCTGATACC
GCTCGCCGCAGCCGAACGACCGAGCGCAGCGAGTCAGTGAGCGAGGAAGCGGAAGAGCGCCTGATGCGGTATTTTCTCCTTACGCATCTGTGCGGTATTTCACACCG
CAATGGTGCACTCTCAGTACAATCTGCTCTGATGCCGCATAGTTAAGCCAGTATACACTCCGCTATCGCTACGTGACTGGGTCATGGCTGCGCCCCGACACCCGCCA
ACACCCGCTGACGCGCCCTGACGGGCTTGTCTGCTCCCGGCATCCGCTTACAGACAAGCTGTGACCGTCTCCGGGAGCTGCATGTGTCAGAGGTTTTCACCGTCATC
ACCGAAACGCGCGAGGCAGCTGCGGTAAAGCTCATCAGCGTGGTCGTGAAGCGATTCACAGATGTCTGCCTGTTCATCCGCGTCCAGCTCGTTGAGTTTCTCCAGAA
GCGTTAATGTCTGGCTTCTGATAAAGCGGGCCATGTTAAGGGCGGTTTTTTCCTGTTTGGTCACTGATGCCTCCGTGTAAGGGGGATTTCTGTTCATGGGGGTAATG
ATACCGATGAAACGAGAGAGGATGCTCACGATACGGGTTACTGATGATGAACATGCCCGGTTACTGGAACGTTGTGAGGGTAAACAACTGGCGGTATGGATGCGGCG
GGACCAGAGAAAAATCACTCAGGGTCAATGCCAGCGCTTCGTTAATACAGATGTAGGTGTTCCACAGGGTAGCCAGCAGCATCCTGCGATGCAGATCCGGAACATAA
TGGTGCAGGGCGCTGACTTCCGCGTTTCCAGACTTTACGAAACACGGAAACCGAAGACCATTCATGTTGTTGCTCAGGTCGCAGACGTTTTGCAGCAGCAGTCGCTT
CACGTTCGCTCGCGTATCGGTGATTCATTCTGCTAACCAGTAAGGCAACCCCGCCAGCCTAGCCGGGTCCTCAACGACAGGAGCACGATCATGCGCACCCGTGGGGC
CGCCATGCCGGCGATAATGGCCTGCTTCTCGCCGAAACGTTTGGTGGCGGGACCAGTGACGAAGGCTTGAGCGAGGGCGTGCAAGATTCCGAATACCGCAAGCGACA
GGCCGATCATCGTCGCGCTCCAGCGAAAGCGGTCCTCGCCGAAAATGACCCAGAGCGCTGCCGGCACCTGTCCTACGAGTTGCATGATAAAGAAGACAGTCATAAGT
GCGGCGACGATAGTCATGCCCCGCGCCCACCGGAAGGAGCTGACTGGGTTGAAGGCTCTCAAGGGCATCGGTCGAGATCCCGGTGCCTAATGAGTGAGCTAACTTAC
ATTAATTGCGTTGCGCTCACTGCCCGCTTTCCAGTCGGGAAACCTGTCGTGCCAGCTGCATTAATGAATCGGCCAACGCGCGGGGAGAGGCGGTTTGCGTATTGGGC
GCCAGGGTGGTTTTTCTTTTCACCAGTGAGACGGGCAACAGCTGATTGCCCTTCACCGCCTGGCCCTGAGAGAGTTGCAGCAAGCGGTCCACGCTGGTTTGCCCCAG
CAGGCGAAAATCCTGTTTGATGGTGGTTAACGGCGGGATATAACATGAGCTGTCTTCGGTATCGTCGTATCCCACTACCGAGATATCCGCACCAACGCGCAGCCCGG
ACTCGGTAATGGCGCGCATTGCGCCCAGCGCCATCTGATCGTTGGCAACCAGCATCGCAGTGGGAACGATGCCCTCATTCAGCATTTGCATGGTTTGTTGAAAACCG
GACATGGCACTCCAGTCGCCTTCCCGTTCCGCTATCGGCTGAATTTGATTGCGAGTGAGATATTTATGCCAGCCAGCCAGACGCAGACGCGCCGAGACAGAACTTAA
TGGGCCCGCTAACAGCGCGATTTGCTGGTGACCCAATGCGACCAGATGCTCCACGCCCAGTCGCGTACCGTCTTCATGGGAGAAAATAATACTGTTGATGGGTGTCT
GGTCAGAGACATCAAGAAATAACGCCGGAACATTAGTGCAGGCAGCTTCCACAGCAATGGCATCCTGGTCATCCAGCGGATAGTTAATGATCAGCCCACTGACGCGT
TGCGCGAGAAGATTGTGCACCGCCGCTTTACAGGCTTCGACGCCGCTTCGTTCTACCATCGACACCACCACGCTGGCACCCAGTTGATCGGCGCGAGATTTAATCGC
CGCGACAATTTGCGACGGCGCGTGCAGGGCCAGACTGGAGGTGGCAACGCCAATCAGCAACGACTGTTTGCCCGCCAGTTGTTGTGCCACGCGGTTGGGAATGTAAT
TCAGCTCCGCCATCGCCGCTTCCACTTTTTCCCGCGTTTTCGCAGAAACGTGGCTGGCCTGGTTCACCACGCGGGAAACGGTCTGATAAGAGACACCGGCATACTCT
GCGACATCGTATAACGTTACTGGTTTCACATTCACCACCCTGAATTGACTCTCTTCCGGGCGCTATCATGCCATACCGCGAAAGGTTTTGCGCCATTCGATGGTGTC
CGGGATCTCGACGCTCTCCCTTATGCGACTCCTGCATTAGGAAGCAGCCCAGTAGTAGGTTGAGGCCGTTGAGCACCGCCGCCGCAAGGAATGGTGCATGCAAGGAG
ATGGCGCCCAACAGTCCCCCGGCCACGGGGCCTGCCACCATACCCACGCCGAAACAAGCGCTCATGAGCCCGAAGTGGCGAGCCCGATCTTCCCCATCGGTGATGTC
GGCGATATAGGCGCCAGCAACCGCACCTGTGGCGCCGGTGATGCCGGCCACGATGCGTCCGGCGTAGAGGATCGAGATCTCGATCCCGCGAAATTAATACGACTCAC
TATAGGGGAATTGTGAGCGGATAACAATTCCCCTCTAGAAATAATTTTGTTTAACTTTAAGAAGGAGATATACCATGAGCGGCCTGAACGATATTTTTGAAGCGCAG
AAAATTGAATGGCATGAAGGCAGCGCTGGAGGTTCAGGTCATCACCACCATCATCACGGTAGTGGTGGAATGGCCCAGGGGACATTGATCCGTGTGACCCCAGAGCA
GCCCACCCATGCCGTGTGTGTGCTGGGCACCTTGACTCAGCTTGACATCTGCAGCTCTGCCCCTGAGGACTGCACGTCCTTCAGCATCAACGCCTCCCCAGGGGTGG
TCGTGGATATTGCCCACAGCCCTCCAGCCAAGAAGAAATCCACAGGTTCCTCCACATGGCCCCTGGACCCTGGGGTAGAGGTGACCCTGACGATGAAAGCGGCCAGT
GGTAGCACAGGCGACCAGAAGGTTCAGATTTCATACTACGGACCCAAGACTCCACCAGTCAAAGCTCTACTCTACCTCACCGCGGTGGAAATCTCCCTGTGCGCAGA
CATCACCCGCACCGGCAAAGTGAAGCCAACCAGAGCTGTGAAAGATCAGAGGACCTGGACCTGGGGCCCTTGTGGACAGGGTGCCATCCTGCTGGTGAACTGTGACA
GAGACAATCTCGAATCTTCTGCCATGGACTGCGAGGATGATGAAGTGCTTGACAGCGAAGACCTGCAGGACATGTCGCTGATGACCCTGAGCACGAAGACCCCCAAG
GACTTCTTCACAAACCATACACTGGTGCTCCACGTGGCCAGGTCTGAGATGGACAAAGTGAGGGTGTTTCAGGCCACACGGGGCAAACTGTCCTCCAAGTGCAGCGT
AGTCTTGGGTCCCAAGTGGCCCTCTCACTACCTGATGGTCCCCGGTGGAAAGCACAACATGGACTTCTACGTGGAGGCCCTCGCTTTCCCGGACACCGACTTCCCGG
GGCTCATTACCCTCACCATCTCCCTGCTGGACACGTCCAACCTGGAGCTCCCCGAGGCTGTGGTGTTCCAAGACAGCGTGGTCTTCCGCGTGGCGCCCTgGATCATG
ACCCCCAACACCCAGCCCCCGCAGGAGGTGTACGCGTGCAGTATTTTTGAAAATGAGGACTTCCTGAAGTCAGTGACTACTCTGGCCATGAAAGCCAAGTGCAAGCT
GACCATCTGCCCTGAGGAGGAGAACATGGATGACCAGTGGATGCAGGATGAAATGGAGATCGGCTACATCCAAGCCCCACACAAAACGCTGCCCGTGGTCTTCGACT
CTCCAAGGAACAGAGGCCTGAAGGAGTTTCCCATCAAACGAGTGATGGGTCCAGATTTTGGCTATGTAACTGCAGGGCCCCAAACAGGGGGTATCAGTGGACTGGAC
TCCTTTGGGAACCTGGAAGTGAGCCCCCCAGTCACAGTCAGGGGCAAGGAATACCCGCTGGGCAGGATTCTCTTCGGGGACAGCTGTTATCCCAGCAATGACAGCCG
GCAGATGCACCAGGCCCTACAGGACTTCCTCAGTGCCCAGCAGGTGCAGGCCCCTGTGAAGCTCTATTCTGACTGGCTGTCCGTGGGCCACGTGGACGAGTTCCTGA
GCTTTGTGCCAGCACCCGACAGGAAGGGCTTCCGGCTGCTCCTGGCCAGCCCCAGGTCCTGCTACAAACTGTTCCAGGAGCAGCAGAATGAGGGCCACGGGGAGGCC
CTGCTGTTCGAAGGGATCAAGAAAAAAAAACAGCAGAAAATAAAGAACATTCTGTCAAACAAGACATTGAGAGAACATAATTCATTTGTGGAGAGATGCATCGACTG
GAACCGCGAGCTGCTGAAGCGGGAGCTGGGCCTGGCCGAGAGTGACATCATTGACATCCCGCAGCTCTTCAAGCTCAAAGAGTTCTCTAAGGCGGAAGCTTTTTTCC
CCAACATGGTGAACATGCTGGTGCTAGGGAAGCACCTGGGCATCCCCAAGCCCTTCGGGCCCGTCATCAACGGCCGCTGCTGCCTGGAGGAGAAGGTGTGTTCCCTG
CTGGAGCCACTGGGCCTCCAGTGCACCTTCATCAACGACTTCTTCACCTACCACATCAGGCATGGGGAGGTGCACTGCGGCACCAACGTGCGCAGAAAGCCCTTCTC
CTTCAAGTGGTGGAACATGGTGCCCTGAGGTGGAAGTGGTCATCACCACCATCATCACTGATGACGAAGCTTGCGGCCGCACTCGAGCACCACCACCACCACCACTG
AGATCCGGCTGCTAACAAAGCCCGAAAGGAAGCTGAGTTGGCTGCTGCCACCGCTGAGCAATAACTAGCATAACCCCTTGGGGCCTCTAAACGGGTCTTGAGGGGTT
TTTTGCTGAAAGGAGGAACTATATCCGGATTGGCGAATGGGACGCGCCCTGTAGCGGCGCATTAAGCGCGGCGGGTGTGGTGGTTACGCGCAGCGTGACCGCTACAC
TTGCCAGCGCCCTAGCGCCCGCTCCTTTCGCTTTCTTCCCTTCCTTTCTCGCCACGTTCGCCGGCTTTCCCCGTCAAGCTCTAAATCGGGGGCTCCCTTTAGGGTTC
CGATTTAGTGCTTTACGGCACCTCGACCCCAAAAAACTTGATTAGGGTGATGGTTCACGTAGTGGGCCATCGCCCTGATAGACGGTTTTTCGCCCTTTGACGTTGGA
GTCCACGTTCTTTAATAGTGGACTCTTGTTCCAAACTGGAACAACACTCAACCCTATCTCGGTCTATTCTTTTGATTTATAAGGGATTTTGCCGATTTCGGCCTATT
GGTTAAAAAATGAGCTGATTTAACAAAAATTTAACGCGAATTTTAACAAAATATTAACGCTTACAATTTAGGTGGCACTTTTCGGGGAAATGTGCGCGGAACCCCTA
TTTGTTTATTTTTCTAAATACATTCAAATATGTATCCGCTCATGAATTAATTCTTAGAAAAACTCATCGAGCATCAAATGAAACTGCAATTTATTCATATCAGGATT
ATCAATACCATATTTTTGAAAAAGCCGTTTCTGTAATGAAGGAGAAAACTCACCGAGGCAGTTCCATAGGATGGCAAGATCCTGGTATCGGTCTGCGATTCCGACTC
GTCCAACATCAATACAACCTATTAATTTCCCCTCGTCAAAAATAAGGTTATCAAGTGAGAAATCACCATGAGTGACGACTGAATCCGGTGAGAATGGCAAAAGTTTA
TGCATTTCTTTCCAGACTTGTTCAACAGGCCAGCCATTACGCTCGTCATCAAAATCACTCGCATCAACCAAACCGTTATTCATTCGTGATTGCGCCTGAGCGAGACG
AAATACGCGATCGCTGTTAAAAGGACAATTACAAACAGGAATCGAATGCAACCGGCGCAGGAACACTGCCAGCGCATCAACAATATTTTCACCTGAATCAGGATATT
CTTCTAATACCTGGAATGCTGTTTTCCCGGGGATCGCAGTGGTGAGTAACCATGCATCATCAGGAGTACGGATAAAATGCTTGATGGTCGGAAGAGGCATAAATTCC
GTCAGCCAGTTTAGTCTGACCATCTCATCTGTAACATCATTGGCAACGCTACCTTTGCCATGTTTCAGAAACAACTCTGGCGCATCGGGCTTCCCATACAATCGATA
GATTGTCGCACCTGATTGCCCGACATTATCGCGAGCCCATTTATACCCATATAAATCAGCATCCATGTTGGAATTTAATCGCGGCCTAGAGCAAGACGTT
```

His Tag
Avi Tag
hPADI4
N394A variant
