# Supplementary material for: A cyclic peptide toolkit reveals mechanistic principles of peptidylarginine deiminase IV regulation
Source: Nat Commun. 2024 Nov 11;15:9746. doi: 10.1038/s41467-024-53554-1 (PMC11555231; doi:10.1038/s41467-024-53554-1)
Supplement: Supplementary file 2 — Description of Additional Supplementary Files [file 41467_2024_53554_MOESM2_ESM.pdf]

Description of Additional Supplementary Files

File Name: Supplementary Data 1

Description: PADI4 RaPID selections Next Generation Sequencing Data

File Name: Supplementary Data 2

Description: Proteomic dataset obtained by Mass Spectrometric analysis of bio-bio-PADI4_7 and bio-PADI4_7scr pull-downs from PADI4-stable mES cells in Serum or KSR conditions.
