# Supplementary material for: A cyclic peptide toolkit reveals mechanistic principles of peptidylarginine deiminase IV regulation
Source: Nat Commun. 2024 Nov 11;15:9746. doi: 10.1038/s41467-024-53554-1 (PMC11555231; doi:10.1038/s41467-024-53554-1)
Supplement: Supplementary file 5 — Reporting Summary [file 41467_2024_53554_MOESM5_ESM.pdf]

nature portfolio

Corresponding author(s): Christophorou M. A., Walport L. J.

Last updated by author(s): Sep 9, 2024

Reporting Summary

Nature Portfolio wishes to improve the reproducibility of the work that we publish. This form provides structure for consistency and transparency in reporting. For further information on Nature Portfolio policies, see our Editorial Policies and the Editorial Policy Checklist.

Statistics

For all statistical analyses, confirm that the following items are present in the figure legend, table legend, main text, or Methods section.

n/a	Confirmed	
☐	☒	The exact sample size (*n*) for each experimental group/condition, given as a discrete number and unit of measurement
☐	☒	A statement on whether measurements were taken from distinct samples or whether the same sample was measured repeatedly
☐	☒	The statistical test(s) used AND whether they are one- or two-sided<br>*Only common tests should be described solely by name; describe more complex techniques in the Methods section.*
☒	☐	A description of all covariates tested
☒	☐	A description of any assumptions or corrections, such as tests of normality and adjustment for multiple comparisons
☐	☒	A full description of the statistical parameters including central tendency (e.g. means) or other basic estimates (e.g. regression coefficient) AND variation (e.g. standard deviation) or associated estimates of uncertainty (e.g. confidence intervals)
☐	☒	For null hypothesis testing, the test statistic (e.g. *F*, *t*, *r*) with confidence intervals, effect sizes, degrees of freedom and *P* value noted<br>*Give P values as exact values whenever suitable.*
☒	☐	For Bayesian analysis, information on the choice of priors and Markov chain Monte Carlo settings
☐	☒	For hierarchical and complex designs, identification of the appropriate level for tests and full reporting of outcomes
☒	☐	Estimates of effect sizes (e.g. Cohen's *d*, Pearson's *r*), indicating how they were calculated

Our web collection on statistics for biologists contains articles on many of the points above.

Software and code

Policy information about availability of computer code

Data collection

Protein Purification was performed using an AKTA Pure (Cytivs)
Recovered library DNA was sequenced on the Illumina HiSeq 4000 or NovaSeq platform with single-ended 100 bp read.
Peptide synthesis was performed using a Liberty Blue Peptide Synthesis System (CEM), a SYRO I (Biotage) or a Activotec P-11 peptide synthesiser.
Peptides were purified by HPLC (Shimadzu) using a Merck Chromolith column (200 X 25 mm)
COLDER assay absorbance was measured using a ClarioSTAR Plate Reader
SPR experiments were conducted on a Biacore S200 or T200.
Isothermal Titration Calorimetry was performed on a MicroCal PEAQ-ITC calorimeter (Malvern Panalytical).
CryoEM data wasa collected using EPU software on a Thermo Scientific Titan Krios microscope operating at 300 kV
Flow cytometry data from CAPA assays was obtained using MACSQuant® VYB Flow cytometer Milteny Biotec
High content microsocpy images were obtained using an InCell6000 (GE/Cytiva) or an ImageXpress Confocal HT.ai (Molecular Devices) high content imaging system through a Nikon PlanApochromat 10x/0.45NA objective lens.
Calcium image experiments were done using a Nikon Wide-field microscope.
Cytotoxiciy data was obtained using a Fortessa flow cytometer and liver imaging was performed using an ® imaging system (Sartorius), using Incucyte® Cytotox Green Dye.
Time-lapse imaging and NET quantification was performed using an an inverted Nikon wide-field microscope system at 37oC and CO2 (5%).
NETosis confocal microscopy was perfomed using a Leica TCS SP8 inverted confocal microscope using sequential scan in between frames mode with a 20x objective.
Western blot membranes were imaged using a Bio-Rad Imager.
Mass spectrometry data was obtained using LC-MS/MS on Q ExactiveTM Plus Orbitrap™ mass spectrometer equipped with an UltiMate 3000

	RSLCNano LC system (both from ThermoFisher).
Data analysis	Data analysis was performed using Prism9 (GraphPad).<br>Image analysis was performed with FIJI or CellPRofiler<br>SPR data were analysed using the Biacore Insight Evaluation Software.<br>ITC data were analysed using MicroCal PEAQ-ITC analysis software.<br>CryoEM data was analysed using RELION, cryoSPARC, Coot and Phenix and Cryo EM figures where generated using UCSF (ChimeraX (version 1.6.1.)<br>Flow cytometry data was analysed using FlowJo (LCC) software.<br>MS data were analysed using Proteome Discoverer 2.5 (Thermo Fisher) using the Sequest HT search engine. Peptides were validated by Percolator with q-value set at 0.01 for the decoy database search, and only high confident PSMs (Peptide Spectrum Matches) were considered.Protein FDR was set at 0.01. Further MS analysis was perfmormed in Perseus.

For manuscripts utilizing custom algorithms or software that are central to the research but not yet described in published literature, software must be made available to editors and reviewers. We strongly encourage code deposition in a community repository (e.g. GitHub). See the Nature Portfolio guidelines for submitting code & software for further information.

Data

Policy information about availability of data

All manuscripts must include a data availability statement. This statement should provide the following information, where applicable:

- Accession codes, unique identifiers, or web links for publicly available datasets
- A description of any restrictions on data availability
- For clinical datasets or third party data, please ensure that the statement adheres to our policy

Unless otherwise noted, all data supporting the results of this work are available in the article, supplementary information, and source data files, or accessible via the public repositories listed herein. CryoEM maps generated in this study have been deposited in the Electron Microscopy Data Bank (EMDB) under the accession codes EMD-19011 (https://www.ebi.ac.uk/emdb/EMD-19011) (PADI4:PADI4_3) and EMD-19012 (https://www.ebi.ac.uk/emdb/EMD-19012) (PADI4:PADI4_11). The atomic coordinates have been deposited in the Protein Data Bank (PDB) under the accession codes 8R8U (https://www.rcsb.org/structure/8R8U) (PADI4:PADI4_3) and 8R8V (https://www.rcsb.org/structure/8R8V) (PADI4:PADI4_11). Mass spectrometry data was deposited to PRIDE depository under the accession code PXD048807 (https://www.ebi.ac.uk/pride/archive/projects/PXD048807). NGS Sequencing data has been uploaded in the NIH Short Read Archive (SRA) under the accession number PRJNA1167468 (https://www.ncbi.nlm.nih.gov/bioproject/1167468).

Research involving human participants, their data, or biological material

Policy information about studies with human participants or human data. See also policy information about sex, gender (identity/presentation), and sexual orientation and race, ethnicity and racism.

Reporting on sex and gender	Information associated with the blood donor is not collected
Reporting on race, ethnicity, or other socially relevant groupings	Information associated with the blood donor is not collected
Population characteristics	Information associated with the blood donor is not collected
Recruitment	Information associated with the blood donor is not collected
Ethics oversight	Peripheral venous blood was isolated from consenting healthy adult volunteers according to approved protocols of the ethics board of the Francis Crick Institute and the Human Tissue act. Written informed consent was obtained from participants. The study was performed in accordance with the ethical standards laid down in the 1964 Declaration of Helsinki and its later amendments.

Note that full information on the approval of the study protocol must also be provided in the manuscript.

Field-specific reporting

Please select the one below that is the best fit for your research. If you are not sure, read the appropriate sections before making your selection.

[x] Life sciences [ ] Behavioural & social sciences [ ] Ecological, evolutionary & environmental sciences

For a reference copy of the document with all sections, see nature.com/documents/nr-reporting-summary-flat.pdf

Life sciences study design

All studies must disclose on these points even when the disclosure is negative.

Sample size	The sample size of each experiment is indicated in the corresponding figure legend and was determined according to our previous experience. The data graphs show the replicates and the mean and standard deviation was calculated.
Data exclusions	In high content microscopy experiments, outliers that were identified using the ROUT function in Prism Graphpad. These outliers were excluded from the experiment.
Replication	For in vitro activation assays 3 independent replicates with 3 technical replicates were performed.<br>For CAPA assays, at least 2 biological replicates were performed. Each point in each biological replicate consists of at least 2 technical replicates.<br>Semi-quantitative biochemical experiments, i.e. coimmunoprecipitation experiments, were similarly repeated to ensure reproducibility. A representative example is shown.<br>All the rest of experiments were repeated at least twice.<br>SPR data was repeated three times.<br>MS. Three replicates of each point were performed<br>All figure legends contain the number of repeats performed for each experiment.
Randomization	No randomization was performed for in vitro experiments.<br>For cell based assays, all experiments were performed with well established cell lines. Pools of cells were randomly distributed in plates and treated with the different stimuli and peptides.
Blinding	Blinding was not used

Reporting for specific materials, systems and methods

We require information from authors about some types of materials, experimental systems and methods used in many studies. Here, indicate whether each material, system or method listed is relevant to your study. If you are not sure if a list item applies to your research, read the appropriate section before selecting a response.

Materials & experimental systems

n/a	Involved in the study	
☐	☒	Antibodies
☐	☒	Eukaryotic cell lines
☒	☐	Palaeontology and archaeology
☒	☐	Animals and other organisms
☒	☐	Clinical data
☒	☐	Dual use research of concern
☒	☐	Plants

Methods

n/a	Involved in the study	
☒	☐	ChIP-seq
☐	☒	Flow cytometry
☒	☐	MRI-based neuroimaging

Antibodies

Antibodies used	anti-H3Cit2 antibody (Abcam ab176843; 1:150 in blocking buffer for high content microscopy, 1:2000 for WB)<br>anti-hPADI4 (Abcam; ab50332; 1:1000)<br>anti histone H3 (Millipore;07-690) (1:0000)<br>anti MPO (DAKO; A0398) (1:2500)<br>anti-histone H3 citrulline R2+R8+R17 (Abcam; ab5103) (WB a:1000 and IF 1:500)<br>donkey anti-rabbit alexa488 (Invitrogen; A21206)<br>Alexa488-conjugated cross-absorbed goat anti-rabbit<br>anti-Bcr (Cell Signalling #3902S; 1:1000 in 3% milk/TBS-T)<br>anti-Impdh2 (Cell Signalling #57068S; 1:1000 in 3% milk/TBS-T).
Validation	All the antibodies used in this study have been reported in provious publications and were commercially available.<br>anti-H3Cit2 antibody (Abcam ab176843)<br>anti-hPADI4 (Abcam; ab50332)<br>anti histone H3 (Millipore;07-690)<br>anti MPO (DAKO; A0398)<br>anti-histone H3 citrulline R2+R8+R17 (Abcam; ab5103)<br>donkey anti-rabbit alexa488 (Invitrogen; A21206)<br>anti-Bcr (Cell Signalling #3902S)

anti-Impdh2 (Cell Signalling #57068S).

Eukaryotic cell lines

Policy information about cell lines and Sex and Gender in Research

Cell line source(s)	mESCells. were obtained from Cambridge Stem Cell Institute UK<br>HaLo-GFP-mito HeLa cell line was profiled by Short Tandem Repeat Analysis (STR) and tested negative for mycoplasma by the Cell Services Technology Platform at the Francis Crick Institute
Authentication	HaLo-GFP-mito HeLa cell line was profiled by Short Tandem Repeat Analysis (STR)
Mycoplasma contamination	mEScells were tested for mycoplasma using the LookOut Mycoplasma PCR Detection Kit (SIGMA)<br><br>Halo-GFP-mito Hela cells tested negative for mycoplama by the Cell Services Technology Platform at the Francis Crick Insitute
Commonly misidentified lines<br>(See ICLAC register)	No commonly misidentified lines were used in this study.

Plants

Seed stocks	N/A
Novel plant genotypes	N/A
Authentication	N/A

Flow Cytometry

Plots

Confirm that:

- [ ] The axis labels state the marker and fluorochrome used (e.g. CD4-FITC).
- [ ] The axis scales are clearly visible. Include numbers along axes only for bottom left plot of group (a 'group' is an analysis of identical markers).
- [ ] All plots are contour plots with outliers or pseudocolor plots.
- [x] A numerical value for number of cells or percentage (with statistics) is provided.

Methodology

Sample preparation	CAPA assays were performed following the protocol published by Peraro et. al51.
Instrument	MACSQuant® VYB Flow cytometer Milteny Biotec
Software	FlowJo
Cell population abundance	No cell sorting used in this publication.
Gating strategy	For each well, single cells were selected with the gate FSC-A/FSC-H. Then, 10000 GFP positive single cells were analysed. TMR mean fluorescence of these 10000 cells was normalised against a positive control (cells treated with Halo-Tag TMR ligand but no peptide) and a negative control (non-treated cells). Gating strategy shown in Supplementary Figure 18.

- [x] Tick this box to confirm that a figure exemplifying the gating strategy is provided in the Supplementary Information.
